# Bioactive Hydroperoxyl Cembranoids from the Red Sea Soft Coral *Sarcophyton glaucum*

**DOI:** 10.3390/md10010209

**Published:** 2012-01-18

**Authors:** Mohamed-Elamir F. Hegazy, Amira M. Gamal Eldeen, Abdelaaty A. Shahat, Fathy F. Abdel-Latif, Tarik A. Mohamed, Bruce R. Whittlesey, Paul W. Paré

**Affiliations:** 1 Chemistry of Medicinal Plants Department, and Center of Excellence for Advanced Sciences, National Research Centre, El-Tahrir Street, Dokki, Giza 12622, Egypt; Email: elamir77@live.com (M.-E.F.H.); aashahat@hotmail.com (A.A.S.); tarik_abdelhalim@yahoo.com (T.A.M.); 2 Cancer Biology Lab, Center of Excellence for Advanced Sciences, and Biochemistry Department, National Research Center, Dokki Cairo 12622, Egypt; Email: aeldeen7@yahoo.com; 3 Medicinal, Aromatic and Poisonous Plants Research Center, College of Pharmacy, King Saudi University, PO Box 2457, Riyadh 11451, Saudi Arabia; 4 Department of Chemistry, Faculty of Science, Minia University, El-Minia 61519, Egypt; Email: drfathyfahim@yahoo.com; 5 Department of Chemistry and Biochemistry, Texas Tech University, Lubbock, TX 79409, USA; Email: bruce.whittlesey@ttu.edu

**Keywords:** *Sarcophyton glaucum*, soft coral, diterpenes, cancer chemo-preventive activity

## Abstract

A chemical investigation of an ethyl acetate extract of the Red Sea soft coral *Sarcophyton glaucum* has led to the isolation of two peroxide diterpenes, 11(*S*) hydroperoxylsarcoph-12(20)-ene (**1**), and 12(*S*)-hydroperoxylsarcoph-10-ene (**2**), as well as 8-*epi*-sarcophinone (**3**). In addition to these three new compounds, two known structures were identified including: *ent*-sarcophine (**4**) and sarcophine (**5**). Structures were elucidated by spectroscopic analysis, with the relative configuration of **1** and **2** confirmed by X-ray diffraction. Isolated compounds were found to be inhibitors of cytochrome P_450_ 1A activity as well as inducers of glutathione *S*-transferases (GST), quinone reductase (QR), and epoxide hydrolase (mEH) establishing chemo-preventive and tumor anti-initiating activity for these characterized metabolites.

## 1. Introduction

Marine natural products are diverse in terms of chemical structures as well as biological activities. The Red Sea serves as an epicenter for marine bio-diversity with a high endemic biota. Indeed of the 180 soft corals species identified world-wide, approximately 40% are native to the Red Sea [1]. Soft corals are marine invertebrates possessing a vast range of terpenoid metabolites. These terpenes, mostly cembranoids, represent the main chemical defense for coral against natural predators [2]. Soft corals of the genus *Sarcophyton* (family Alcyoniidae) are particularly rich in cembrane terpenes [3]. Cembranoids contain a 14-membered macro cyclic skeleton and exhibit a wide range of biological activities including anti-tumor, neuro-protective, antimicrobial, calcium-antagonistic, and anti-inflammatory activity [4,5,6,7]. The cembranoid diterpene sarcophine has been investigated since 1998 for its potential as a chemo-preventive agent [8], cytotoxic agent, anti-microbial agent [9], competitive cholinesterase inhibitor [10], noncompetitive phosphofructokinase inhibitor [11], and a Na^+^, K^+^-ATPase inhibitor [12]. Recent studies focusing on the treatment of human diseases have shown that sarcophine and sarcophine derivatives (e.g., hydroxylated sarcophine) are potent cancer chemo-preventive agents [8,9,13,14,15]. 

Cancer chemoprevention is based on chemical constituents that block, inhibit, or reverse the development of cancer in normal or pre-neoplastic tissue [16]. During the past 20 years, thousands of novel marine metabolites have been identified and assayed for anticancer activity [17]. Most of these drug leads are identified by high-throughput *in vitro* screening via a cost-effective testing of cancer cell lines derived from human and rodent sources. Indeed several marine-derived drug leads have reached phase II human clinical trials based on promising anticancer results, although toxicity testing has mostly screened out such candidate drugs. Sarcophine anti-tumor potency appears to at least in part involve inhibition of cell transformation that can be induced *in vitro* by 12-*O*-tetradecanoyl phorbol-13-acetate (TPA) with irreversible acquisition of tumorigenicity [7,13]. In many cases, carcinogenesis is initiated by pro-carcinogens in combination with phase I enzymes such as cytochrome P_450_ 1A and oxidative stress leading to DNA damage. This process can be mitigated at least in part by phase II detoxification enzymes such as glutathione *S*-transferases (GSTs), quinone reductase (QR), and epoxide hydrolase (mEH). 

**Chart 1:**
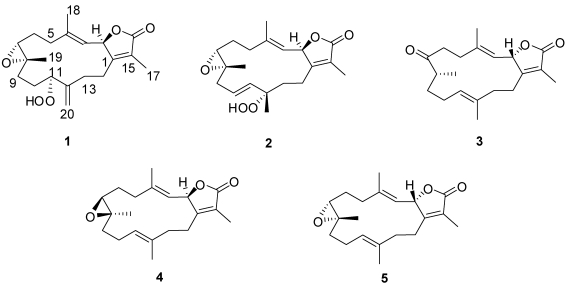
Structures of metabolites **1**–**5**.

Herein, we report the isolation of three new and two known cembranolides (Chart 1) from an ethyl acetate extraction of the Red Sea soft coral *Sarcophyton glaucum*. Structures of these isolated metabolites were elucidated by 1D and 2D spectroscopic techniques, while the absolute configuration of **1** and **2** were confirmed by X-ray diffraction and circular dichroism (CD) analyses. Compounds **2** and **3** were found to be promising inhibitors of cytochrome P_450_ 1A activity as well as inducers of GST and QR activity in *in vitro* assays.

## 2. Results and Discussion

Freshly collected specimens of *S. glaucum* were immediately frozen in dry ice and kept at −20 °C until ready for organic-solvent extraction. The EtOAc-soluble fraction was subjected to normal and reverse phase chromatography to afford new hydroperoxyl cembranolides (**1** and **2**), a cembrene derivative 8-*epi*-sarcophinone (**3**) along with two known cembranolides, *ent*-sarcophine (**4**) [18,19], and sarcophine (**5**) [18,19,20,21]. 

Preliminary ^1^H NMR analysis established that all fractions shared a common carbon skeleton, differing either in the degree of oxidation or the configuration of one or more chiral centers. Precedent from soft coral literature led to the assumption of a cembranoid-skeleton backbone [19]. Compound **1** was obtained as colorless crystals, 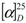 +12.6 (*c* 0.09, CHCl_3_). The HR-FAB-MS exhibited a [M + Na]^+^ ion at *m/z* 371.18281, indicating a molecular formula of C_20_H_28_O_5_Na and seven degrees of unsaturation that was supported by NMR data. An IR spectrum indicated the presence of an α,β-unsaturated-γ-lactone (1750 and 1686 cm^−1^), a carbonyl (1707 cm^−1^), an olefin (1669 cm^−1^), an epoxide (1256 cm^−1^) and a broad absorption band for OH stretching (3000–3353 cm^−1^). The ^13^C NMR and DEPT spectrum (Table 1) exhibited 20 carbon signals establishing: three methyls, seven methylenes, four methines, and six quaternary carbons. The spectrum also revealed the presence of an exomethylene functionality at *δ*_C_ 113.4/144.5, two oxymethine carbons at *δ*_C_ 60.8 and 86.5, one oxygenated quaternary carbon at *δ*_C_ 61.1, and two olefinic carbons at *δ*_C_ 119.6 and 146.0.

**Figure 1:**
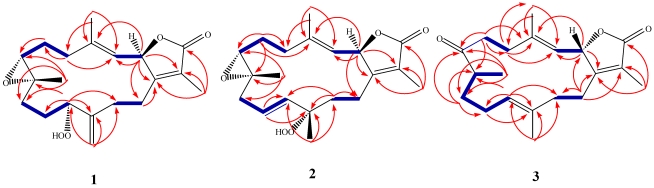
Selected ^1^H-^1^H COSY (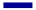) and HMBC (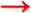) correlations of **1**–**3**.

The low field oxymethine carbon at *δ*_C_ 86.5 (C-11) suggested the presence of a peroxide functionality that is consistent with the presence of a broad singlet at *δ*_H_ 8.25 in the ^1^H NMR spectrum [18]. ^13^C NMR analysis indicated that two oxygens contribute to an α,β-unsaturated-γ-lactone with appropriate signals at *δ*_C_ 174.5 and 78.9 for the carbonyl and oxymethine carbons, respectively. The olefinic methyl group at *δ*_H_ 1.85 (H_3_-17) exhibited an HMBC correlation with a low-filed ^13^C NMR resonance for a keto group in association with the α,β-unsaturated-γ-lactone ring at *δ*_C_ 174.5 (C-16). Carbon signals at *δ*_C_ 124.2 (C-15) and 162.2 (C-1) were consistent with α and β olefinic carbons of the α,β-unsaturated-γ-lactone system. The carbon signal at *δ*_C_ 78.9 (C-2) is consistent with an oxymethine carbon while the oxymethine proton at *δ*_H_ 5.50 (d, *J* = 15.0 Hz; H-2) exhibited a strong correlation with a one-proton doublet at *δ*_H_ 5.09 (*J* = 15.0 Hz; H-3) in the ^1^H-^1^H COSY spectrum (Figure 1). The olefinic methyl group at *δ*_H_ 1.94 (H-18) also shows an HMBC correlation with an olefinic methine at *δ*_C_ 119.6 (C-3). The methyl signal at *δ*_H_ 1.27 (H-19) indicates a proximal oxygen functionality identified from ^13^C NMR to be an epoxide. The location of the epoxide ring at C7/C-8 was detected from HMBC correlations (Figure 1), as there are clear correlations between C-7 (*δ*_C_ 60.8) and H-6 (2.59, td, *J* = 5.0, 13.5 Hz; 2.39, m), H-5 (2.20, m; 2.39, m), H_3_-19, and H-9 (1.30, m; 1.79, m); and between C-8 (*δ*_C_ 61.1) and H-7 (2.50, dd, *J* = 4.5, 8.5 Hz), H-9, H-10, and H-6. A triplet-like signal at *δ*_H_ 4.35 (*J* = 5.0 Hz; H-11) revealed the presence of a peroxide at *δ*_C_ 86.5 that showed a strong correlation with methylene signals at *δ*_H_ 1.50 (m) and 1.70 (m) (H-10) in the ^1^H-^1^H COSY spectrum (Figure 1). The position of the peroxide was established through HMBC correlation between H-11 and C-9 (32.1, t), C-10 (26.7, t), C-12 (144.5, s), and C-13 (30.1, t). Exomethylene protons at *δ*_H_ 5.12 (s) and 5.16 (s) (H_2_-20) showed strong correlations with carbon signals at *δ*_C_ 113.4 (C-20) and *δ*_C_ 144.5 (C-12) in HMQC and HMBC analyses, respectively.

**Table 1:** ^1^H and ^13^C NMR spectral data of **1**–**3**.

Position	1		2		3	
	*δ*_H_ (*J* in Hz)	*δ*_C_	*δ*_H_ (*J* in Hz)	*δ*_C_	*δ*_H_ (*J* in Hz)	*δ*_C_
1	--	162.2	--	162.5	--	163.3
2	5.50 (d, 15.0)	78.9	5.44 (d, 16.0)	79.2	5.47 (dd, 1.5, 10.0)	78.9
3	5.09 (d, 15.0)	119.6	4.98 (d, 16.0)	118.9	5.12 (brd, 10.5)	122.1
4	--	146.0	--	146.7	--	141.9
5	2.20 (m)	35.9	2.02 (m)	37.1	2.66 (m)	37.9
	2.39 (m)		2.38 (dt, 4.5, 13.5)		2.76 (m) *	
6	2.59 (td, 5, 13.5)	25.4	1.77 (m)	25.0	2.06 (m)	32.8
	2.39 (m)				2.73 (m) *	
7	2.50 (d, 4.5, 8.5)	60.8	2.53 (dd, 5.0, 6.0)	59.0	--	212.1
8	--	61.1	--	59.2	2.44 (m)	46.6
9	1.30 (m)	32.1	2.25 (m)	39.0	1.56 (m)	32.4
	1.79 (m)		2.46 (m)		1.95 (m)	
10	1.50 (m)	26.7	5.42 (ddd, 16.0, 10.5, 7.5)	124.6	1.88 (brd, 11.0)	26.5
	1.70 (m)				2.26 (m)	
11	4.35 (t like, 5)	86.5	5.56 (d, 16.0)	136.1	4.78 (td, 7.5, 1)	124.1
12	--	144.5	--	84.0	--	134.9
13	2.07 (m)	30.1	1.41 (dd, 4) *	37.6	1.92 (m)	36.1
	2.20 (m)		2.07 (td, 13.0, 4.5)		2.00 (m)	
14	2.07 (m)	24.8	2.42 (m) *	21.2	2.16 (brt, 12.0)	26.1
	1.50 (m)		2.50 (m) *		2.60 (m)	
15	--	124.2	--	123.8	--	122.3
16	--	174.5	--	174.9	--	175.0
17	1.85 (s)	8.9	1.87 (brs)	9.1	1.82 (t, 1.5)	8.9
18	1.94 (s)	16.0	1.89 (s)	16.2	1.84 (s)	16.2
19	1.27 (s)	16.7	1.30 (s)	18.2	1.06 (d, 7.5)	18.8
20	5.12 (s)	113.4	1.43 (s)	22.8	1.60 (s)	15.7
	5.16 (s)					
-OOH	8.25 (brs)	--	7.70 (brs)	--	--	--

*^a^* Recorded in CDCl_3_ and obtained at 500 and 125 MHz for ^1^H and ^13^C NMR, respectively. * Overlapping signals.

**Figure 2:**
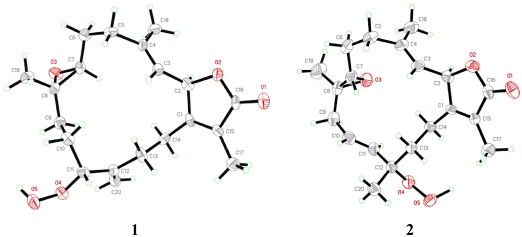
ORTEP depiction for X-ray crystal structures of **1**–**2**.

**Figure 3:**
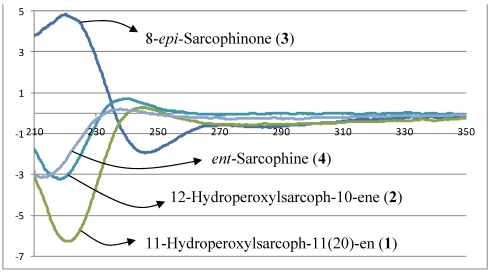
Circular dichroism (CD) spectra of **1**–**4**.

Comparison of the above data with those structural relatives isolated from the same species [22,23], strongly indicated a cembranoid molecular framework containing the rare 11-peroxid-12(20)-exomethylene as confirmed by X-ray analysis (Figure 2). The relative configuration of **1** was determined on the basis of coupling constants and NOESY experiments. The vicinal coupling constant of 15.0 Hz between H-2 and H-3 as well as a NOESY correlation of H-2 with H_3_-18 established a trans configuration between the γ-lactone (H-2) and the olefinic proton (H-3). In order to confirm the position of the peroxyl group, as well as the relative stereochemistry, X-ray structure analysis was performed. The absolute stereochemistry of **1** at C-2 was determined via circular dichroism (CD) analysis (Figure 3). The observed positive Cotton effect {[θ]_248_ +0.7} followed by a negative value {[θ]_225_ −3.23} observed in the CD spectrum for the electronic transitions of the 2(5*H*)-furanone moiety, indicated a left hand (*M*) helix configuration for the five-membered α,β-unsaturated-γ-lactone ring [24]. Supporting CD data for **1**, CD spectral comparison between **1** and *ent*-sarcophine (**4**) indicated the same *R* absolute configuration for the two compounds at C-2 [18,19,21,22]. Therefore, **1** was assigned as 11(*S*)-hydroperoxylsarcoph-12(20)-ene. 

Compound **2** was obtained as color-less crystals, 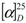 −20.1 (*c* 0.1, CHCl_3_) with much of the spectral data identical to **1** (Table 1). The HR-FAB-MS showed an [M + Na]^+^ ion at *m/z* 371.18293 indicating a molecular formula C_20_H_8_O_5_Na and seven degrees of unsaturation that was supported by NMR data. The analysis of ^1^H, ^13^C NMR and DEPT spectra revealed the presence of four methyls, five methylenes, five methines (two of them oxygenated, *δ*_C_ 59.0, and 79.2) , and six quaternary carbons (two of them oxygenated, *δ*_C_ 59.2, and 84.0). NMR spectra also revealed the presence of four olefinic functionalities at *δ*_C_ 118.9, 124.6, 136.1 and 146.7. The presence of an α,β-unsaturated-γ-lactone functionality was assigned based on NMR parallels with **1**. From HMBC (Figure 1), a methyl unit (1.43, s; H_3_-20) was observed proximal to C-12 determined from correlations between C-12 (*δ*_C_ 84.0) and H_3_-20 (1.43, s), H-11 (5.56, d, *J* = 16.0 Hz), H-10 (5.42, ddd, *J* = 16.0, 10.5, 7.5 Hz), H-13 (2.07, td, *J* = 13.0, 4.5 Hz; 1.41, dd, *J* = 4 Hz, overlapped with H_3_-18). HMBC correlations (Figure 1) were also observed between C-7 (*δ*_C_ 59.0) and H-6 (1.77, m, 2H), H-5 (2.02, m; 2.38, dt, *J* = 4.5, 13.5 Hz), H_3_-19 (1.30, s), and H_2_-9 (2.25, m; 2.46, m), and C-8 (*δ*_C_ 59.2) and H-7 (2.53, dd, *J* = 5.0, 6.0 Hz), H_2_-9 (2.25, m; 2.46, m), H-10 (5.42), and H_2_-6 (1.77, m, 2H) indicating the same epoxide location as in **1** bridging C-7 and C-8. The olefinic proton signal at *δ*_H_ 5.56 (H-11, d, *J* = 16.0 Hz) showed an HMBC correlation with an oxygenated carbon at *δ*_C_ 84.0 (C-12), a methyl signal at *δ*_C_ 22.8 (C-20), and an olefinic carbon at *δ*_C_ 124.6 (C-10) establishing that the peroxyl and double bond functionalities are located at C-12 and C-10/C-11, respectively.

The combined spectral data indicated a cembranoid molecular framework containing a rare 12-peroxid-10-ene. This chemical configuration was confirmed by X-ray analysis (Figure 2) and HMBC correlations (Figure 1). The relative configuration of **2** was determined on the basis of coupling constants and NOESY experiments. The germinal coupling between H-2 and H-3 (16.0 Hz) and a NOESY correlation between H-2 and H_3_-18 indicated a trans configuration between the γ-lactone (H-2) and olefinic protons (H-3). The absolute stereochemistry of **2** was determined via CD analysis with the CD spectra (Figure 3) of **2** nearly equivalent with **1** and **4** establishing the same (*R*) configuration at C-2 [18,19,21,22]. Therefore, compound **2** was assigned to be 12-hydroperoxylsarcoph-10-ene (**2**).

Compound **3** was obtained as a color-less oil, 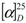 +19.2 (*c* 0.1, CHCl_3_). The HR-FAB-MS showed an [M + Na]^+^ ion at *m/z* 339.19313 suggesting a molecular formula of C_20_H_28_O_3_Na that was supported by NMR data. Spectral data suggested that **3** was similar to the sarcophinone previously isolated from the soft coral *Sarchophyton molle* Tix [23], except for an up-field shift for H_3_-19 (*δ*_H_ 1.06) and an increase in its coupling constant (7.5 Hz) in comparison with sarcophinone H_3_-19 (*δ*_H_ 1.13, *J* = 6.4 Hz). This up-field shift for such a methyl attached to a methine carbon can be explained by an alternative stereochemistry since the β-configuration methyl group is down-field relative to the α-stereochemistry [23,24]. 

The location of the ketone carbonyl group at C-8 was determined from HMBC data that established clear correlations with H-8 (*δ*_H_ 2.44, m)/H_3_-19 (*δ*_H_ 1.06, d, *J* = 7.5), H-9 (*δ*_H_ 1.56 and 1.95, m, 2H), H-6 (*δ*_H_ 2.06 and 2.73, m, 2H), and H-5 (*δ*_H_ 2.66 and 2.76, m, 2H) (Figure 1). The absolute stereochemistry at C-2 was determined by CD analysis in which the spectrum was dominated by negative and positive Cotton effects {[θ]_245_ −1.9, [θ]_220_ +4.8} (Figure 3) due to the electronic transitions of the 2(5*H*)-furanone moiety [15]. These Cotton effects indicated a right-handed (*P*) helix for the five-membered α,β-unsaturated-γ-lactone ring. Similar CD spectra for **3** and sarcophine (**5**) show a common *S* configuration at C-2 [11,12,14,15]. Compound **3** was therefore identified as 8-*epi*-sarcophinone. There are two reports that have the same structure as **3** and are referred to as *iso*-sarcophinone [25,26]; however with an absence of spectral data, direct comparisons cannot be made. In a more comprehensive study of *iso*-sarcophinone by Su *et al.* [23] full ^1^H and ^13^C NMR data is provided and the reported compound is an epimer of **3** with the opposite stereochemistry at C-8; this epimer of **3** has also been named as *iso*-sarcophinone. Since only spectral data comparisons are possible for the Su *et al.* study [23] and the NMR data for compound **3** reported here are not consistent with *iso*-sarcophinone, we propose that *iso*-sarcophinone has not been isolated in the present study but instead a new 8-*epi*-sarcophinone as shown in **3**. Whether 8-*epi*-sarcophinone was isolated and not appropriately named or *iso*-sarcophinone was isolated but incorrectly identified by Czarkie *et al*. [25] is uncertain with an absence of key spectral data. With this study, spectral data is now available for both *iso*-sarcophinone [23] and 8-*epi*-sarcophinone. 

**Figure 4:**
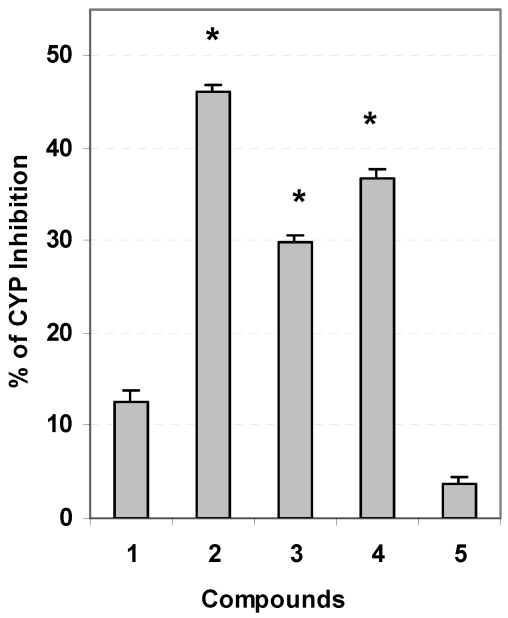
Enzyme regulation of cancer metabolism by extracted soft coral components. Cytochrome P_450_ 1A inhibition (assayed concentration 1 µg/mL, mean ± SD, *n* = 4). Asterisk (*) indicates significant different *p* < 0.05.

To examine the anti-cancer activity of characterized *S. glaucum* metabolites, individual components were assayed for inhibition of the phase I enzyme cytochrome P_450_ 1A since the enzyme in combination with pro-carcinogens and/or oxidative stress can lead to DNA damage. Compounds **2**, **3**, and **4** were identified as inhibitors of Cyp1A activity (*p* < 0.01) with IC_50_ values of 2.7, 3.7 and 3.4 nM respectively (Figure 4), compared with the initial activity of β-naphthoflavone-stimulated cells. Assayed compounds **1** and **5** exhibited insignificant inhibition of Cyp1A activity (*p* > 0.05).

**Figure 5:**
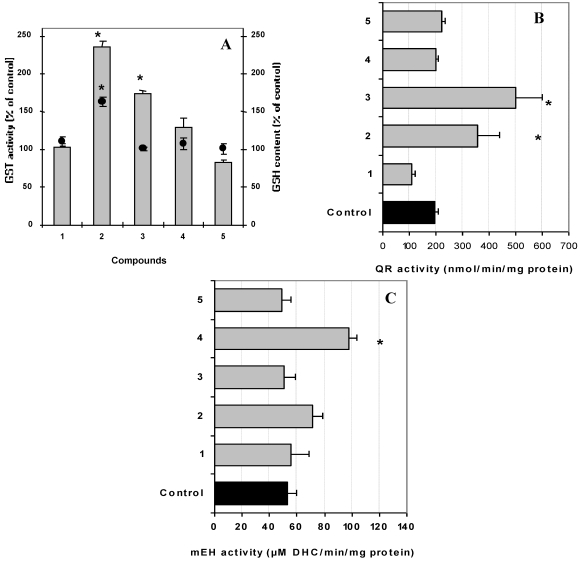
Anti-initiating activity through the modulation of carcinogen metabolism. Effect of treatment with 10 µg/mL of each sample for 48 h on glutathione-*S*-transferase activity (bars) and non-enzymatic antioxidant activity GSH (circles) (**A**), quinone reductase (QR) (**B**) and epoxide hydrolase mEH (**C**) activities in Hepa1c1c7 cells. Data expressed as mean ± SD (*n* = 4).

To examine induction of protective enzymes of oxidative stress by characterized *S. glaucum* metabolites, individual components were assayed for induction of glutathione-*S*-transferase activity, quinone reductase (QR) and epoxide hydrolase (mEH). GSTs are responsible for the detoxification of a wide range of substrates including xenobiotics as well as occupational and environmental carcinogens such as pesticides and polycyclic aromatic hydrocarbons [27]. Total GST activity was investigated in cultured Hepa1c1c7 cells. Forty eight hours after murine hepatoma cell culture incubation with 10 µg/mL of each metabolite, total GSTs activity was significantly induced by **2**–**3** (*p* < 0.01 and *p* < 0.05, respectively) (Figure 5A). While free thiols that serve as non-enzymatic antioxidants assisting in counteracting the deleterious effect of ROS were significantly elevated in cell cultures only when treated with **2** (10 µg/mL) (*p* < 0.05) (Figure 5A). 

QR that is induced coordinately with other Phase II enzymes such as GSTs and contributes to quinone detoxification was investigated in murine hepatoma cell culture. After 48 h incubation, **2**–**3** resulted in a significant induction of QR activity (*p* < 0.01 and *p* < 0.05, respectively) (Figure 5B). In contrast, epoxide hydrolase mEH, an important metabolic enzyme that catalyzes the addition of water to alkene epoxides and arene oxides [28] was significantly elevated in cell cultures only when treated with **4** (10 µg/mL) (*p* < 0.05) (Figure 5C). 

## 3. Experimental Section

### 3.1. General Experimental Procedures

^1^H and ^13^C NMR spectra were recorded in CDC_l3_ on a Varian MercuryPlus 300 MHz and Varian Unity INOVA 500 spectrometer (300/500 MHz for ^1^H and 75/125 MHz for ^13^C, respectively). All chemical shifts (*δ*) are given in ppm units with reference to TMS as an internal standard and the coupling constants (*J*) are given in Hz. FAB-MS was performed on a Finnigan LCQ ion trap mass spectrometer and HR-FAB-MS experiments were performed on Fourier transform ion cyclotron mass spectrometer (Ion Spec, Varian). The spectra were recorded by infusion into the ESI (electrospray ionization) source using chloroform as the solvent. High performance liquid chromatography (HPLC) was performed on an Agilent pump equipped with an Agilent-G1314 variable wavelength UV detector at 254 nm and a semi-preparative reverse-phase column (Econosphere™, RP-C18, 5 μm, 250 × 4.6 mm). Optical rotation was determined at 589 nm (sodium D line) using a Perkin–Elmer-341 MC digital polarimeter; [α]_D_-values are given in the unit of 10 deg^−1^·cm^2^·g^−1^. CD was measured with an OLIS, DSM-10 UV/Vis CD. 

Silica gel 60 (Merck, 230–400 mesh) and Sephadex LH-20 (Sigma) were used for column chromatography. Pre-coated silica gel plates (Merck, Kieselgel 60 F_254_, 0.25 mm) were used for TLC analyses. Spots were visualized by heating after spraying with 10% H_2_SO_4_.

### 3.2. Animal Material

Soft coral *S. glaucum* was collected from the Egyptian Red Sea coast of Hurghada in June, 2009. A voucher specimen (03RS24) was deposited in the National Institute of Oceanography and Fisheries, Marine Biological Station, Hurghada, Egypt. 

### 3.3. Extraction and Separation

The frozen soft coral was chopped into small pieces (4 kg, wet weight) and extracted with ethyl acetate at room temperature (4 L × 5). The combined ethyl acetate extracts were concentrated to a brown gum. The dried EtOAc-soluble material (20.0 g) was subjected to gravity chromatography on silica gel column (6 × 120 cm) using *n*-hexane–EtOAc (gradient separation) into 8 fractions. Fraction 3 (2.2 g) eluted with *n*-hexane–EtOAc (8:1) was subjected to silica gel column separation to afford **5** (50 mg). The remaining samples of this fraction were collected and purified by Sephadex LH-20 using hexane–CHCl_3_–MeOH (7:4:0.5) followed by reverse phase HPLC using acetonitrile H_2_O (1:1) to afford **1** (35 mg), **2** (23 mg) and **3** (14 mg). Fraction 4 eluted with *n*-hexane–EtOAc (6:1) was re-purified on reverse phase HPLC using acetonitrile/H_2_O (50–100% H_2_O) **4** (9 mg). 

11-hydroperoxylsarcoph-11(20)-ene (**1**): colorless crystal; 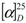 = +12.6 (*c* 0.09, CHCl_3_); IR (KBr) ν_max_ 3353, 3000, 1750, 1707, 1686, 1669, 1256 cm^−1^; ^1^H NMR and ^13^C NMR data, see Table 1; HR-FAB-MS [M + Na]^+^ *m/z* 371.18281 (41%) (calc. 371.18284, C_20_H_28_O_5_Na).

#### 3.3.1. Single-Crystal X-ray Crystallography of **1**

X-ray intensity data were measured on a Bruker Smart Apex II automated X-ray diffractometer equipped with a CCD detector. The frames were integrated with the Bruker SAINT Software package (Version 6) using a narrow-frame algorithm. Integration of the data using a monoclinic unit cell yielded a total of 4644 reflections to a maximum θ angle of 18.45° (1.12 Å resolution), of which 1364 were independent (average redundancy 3.405, completeness = 99.9%, *R*_int_ = 3.57%, *R*_sig_ = 3.55%) and 1257 (92.16%) had intensities greater than 2σ(F2). The final cell constants are based upon the refinement of the XYZ-centroids of 56 reflections with intensities greater than 20 σ(I) and 2θ values in the range 6.72° < 2θ < 23.48°. The calculated minimum and maximum transmission coefficients (based on crystal size) are 0.9274 and 0.9982, respectively.

The structure was solved and refined using the Bruker SHELXTL Software Package in the space group P21 (No. 4 in the International Tables for X-ray Crystallography [29]), with Z = 2 for the formula C_20_H_28_O_5_. The final anisotropic full-matrix least-squares refinement on F2 with 339 variables converged at R1 = 2.54% for the observed data (intensities greater than 4σ(F2)) and wR2 = 5.13% for all data, with a goodness-of-fit value of 1.075. The largest peak in the final difference electron density synthesis was 0.071 e^−^/Å3, and the largest hole was −0.079 e^−^/Å3, with an RMS deviation of 0.020 e^−^/Å3.

12-Hydroperoxylsarcoph-10-ene (**2**): colorless crystal; 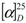 = −20.1 (*c* 0.1, CHCl_3_); IR (KBr) ν_max_ 3353, 3000, 1750, 1707, 1686, 1669, 1256 cm^−1^; ^1^H NMR and ^13^C NMR data, see Table 1; HR-FAB-MS [M + Na]^+^ *m/z* 371.18293 (33%) (calc. 371.18290, C_20_H_28_O_5_Na).

#### 3.3.2. Single-Crystal X-ray Crystallography of **2**

X-ray intensity data were measured on a Bruker Smart Apex II automated X-ray diffractometer equipped with a CCD detector. The frames were integrated with the Bruker SAINT Software package (Version 6) using a narrow-frame algorithm. Integration of the data using an orthorhombic unit cell yielded a total of 9176 reflections to a maximum θ angle of 17.97° (1.15 Å resolution), of which 1332 were independent (average redundancy 6.889, completeness = 100%, *R*_int_ = 4.16%, *R*_sig_ = 2.43%) and 1255 (94.22%) had intensities greater than 2σ(F2). The final cell constants are based upon the refinement of the XYZ-centroids of 105 reflections with intensities greater than 20 σ(I) and 2θ values in the range 4.15° < 2θ < 36.10°. Data were corrected for absorption effects using the multi-scan method (SADABS). The ratio of minimum to maximum apparent transmission was 0.899. The structure was solved and refined using the Bruker SHELXTL Software Package in the space group P212121 (No. 19 in the International Tables for X-ray Crystallography [29]), with Z = 4 for the formula C_20_H_28_O_5_. The final anisotropic full-matrix least-squares refinement on F2 with 235 variables converged at R1 = 2.15% for the observed data (intensities greater than 4σ(F2)) and wR2 = 4.66% for all data, with a goodness-of-fit value of 1.042 and a data-to-parameter ratio of 5.7. The largest peak in the final difference electron density synthesis was 0.069 e^−^/Å3, and the largest hole was −0.079 e^−^/Å3, with an RMS deviation of 0.017 e^−^/Å3.

8-*epi-*Sarcophinone (**3**): colorless crystal; 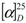 = +19.2 (*c* 0.1, CHCl_3_); IR (KBr) ν_max_ 1730, 1700, 1410, 1230, 960 cm^−1^; ^1^H NMR and ^13^C NMR data, see Table 1; HR-FAB-MS [M + Na]^+^ *m/z* 339.19313 (100%) (calc. 339.19317, C_20_H_28_O_5_Na).

*ent*-Sarcophine (**4**): 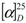 = −20.0 (*c* 0.03, CHCl_3_); lit. 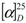 = −80.0 (*c* 0.3, CHCl_3_) [19].

(+)-Sarcophine (**5**): 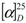 = +95.0 (*c* 0.5, CHCl_3_); lit. 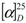 = +92 (*c* 1.0, CHCl_3_) [20].

### 3.4. Cell Culture

Murine hepatoma cells (Hepa1c1c7) was purchased from the American Type Culture Collection. Cells were cultured on Dulbeco’s Modified Eagle’s medium (DMEM). Media were supplemented with 10% fetal bovine serum (FBS), 2 mM L-glutamine, containing 100 U/mL penicillin G sodium, 100 U/mL streptomycin sulfate, and 250 ng/mL amphotericin B. Cells were maintained in humidified air containing 5% CO_2_ at 37 °C. The monolayer cells were harvested using trypsin/EDTA. All experiments were repeated four times, unless mentioned, and the data were represented as mean ± SD. The extract, fractions and compounds were dissolved in DMSO (99.9%) and diluted 1000 fold for each assay. In all cellular experiments, results were compared with DMSO-treated cells. All cell culture material was obtained from Cambrex, BioScience (Copenhagen, Denmark). 

### 3.5. Evaluation of Carcinogen Metabolizing Enzymes

Cytochrome P450 1A (Cyp1A) activity was determined by the rate of dealkylation of 3-cyano-7-ethoxycoumarin (CEC) to the fluorescent 3-cyano-7-hydroxycoumarin based on Crespi *et al.* [30], and modified by Gerhäuser *et al.* [31]. Homogenates from cultured Hepa1c1c7 cells, induced with β-naphthoflavone (1 µg/mL final concentration) were used as a source of Cyp1A. The rate of CEC conversion was measured kinetically at excitation 408/20 nm and emission 460/40 nm by a microplate fluorescence reader (FluoStarOptima, BMG lab technologies, Durham, NC, USA). Inhibition of Cyp1A activity was calculated in comparison with the initial fluorescence of a complete reaction mixture with cell homogenate and buffer instead of the assay compound.

Hepa1c1c7 cells (1 × 10^6^) were incubated with the compounds (10 µg/mL) for 48 h. Glutathione-*S*-transferase (GST) activity was measured in the cell lysate according to Habig *et al.* [32] and based on GST-catalyzed reaction between GSH and 1-chloro-2,4-dinitrobenzene that acts as an electrophilic substrate for GST. In the kinetic analysis, the absorbance was assessed at 340 nm. GSTs were normalized to the protein content as measured by bicinchoninic acid assay [33]. Quinone reductase (QR) activity was determined by measuring the reduction of 2,6-dichloroindophenol [34]. The specific QR activity was expressed as nmol of 2,6-dichloroindophenol reduced by 1 mg of protein within 1 min. Enzyme activity for mEH was assessed by the production rate of 7-(29,39-dihydroxy) propoxycoumarin (DHC) from 7-glycidoxycoumarin (GOC), as described by Inoue *et al.* [35]. The fluorescence intensity was measured at excitation 325 nm and emission 391 nm. DHC in methanol was used as a standard. The enzyme activity was expressed as µM DHC/min/mg protein.

### 3.6. Statistical Analysis

Data were analyzed by a one-way ANOVA followed by a *post hoc* Turkey test; *p* < 0.05 indicated statistical significance.

## 4. Conclusions

Three new (**1**–**3**) and two known cembranolides (**4** and **5**) were isolated and chemically characterized from the Red Sea soft coral *Sarcophyton glaucum*. The absolute configuration of **1** and **2** were confirmed by X-ray diffraction and circular dichroism (CD) analyses. Compounds **2** and **3** were found to be promising inhibitors of cytochrome P_450_ 1A activity as well as inducers of GST and QR activity in *in vitro* assays.

## Supplementary Files

Supplementary File 1: PDF-Document (PDF, 13611 KB)
