# Supplementary material for: Bioactive Hydroperoxyl Cembranoids from the Red Sea Soft Coral Sarcophyton glaucum
Source: Mar Drugs. 2012 Jan 18;10(1):209–22. doi: 10.3390/md10010209 (PMC3280536; doi:10.3390/md10010209)
Supplement: Supplementary File 1: — PDF-Document (PDF, 13611 KB) [file marinedrugs-10-00209-s001.pdf]

Re-3-5-55
in CDCl3
SW Probe
mkh031810.1

Pulse Sequence: s2pul

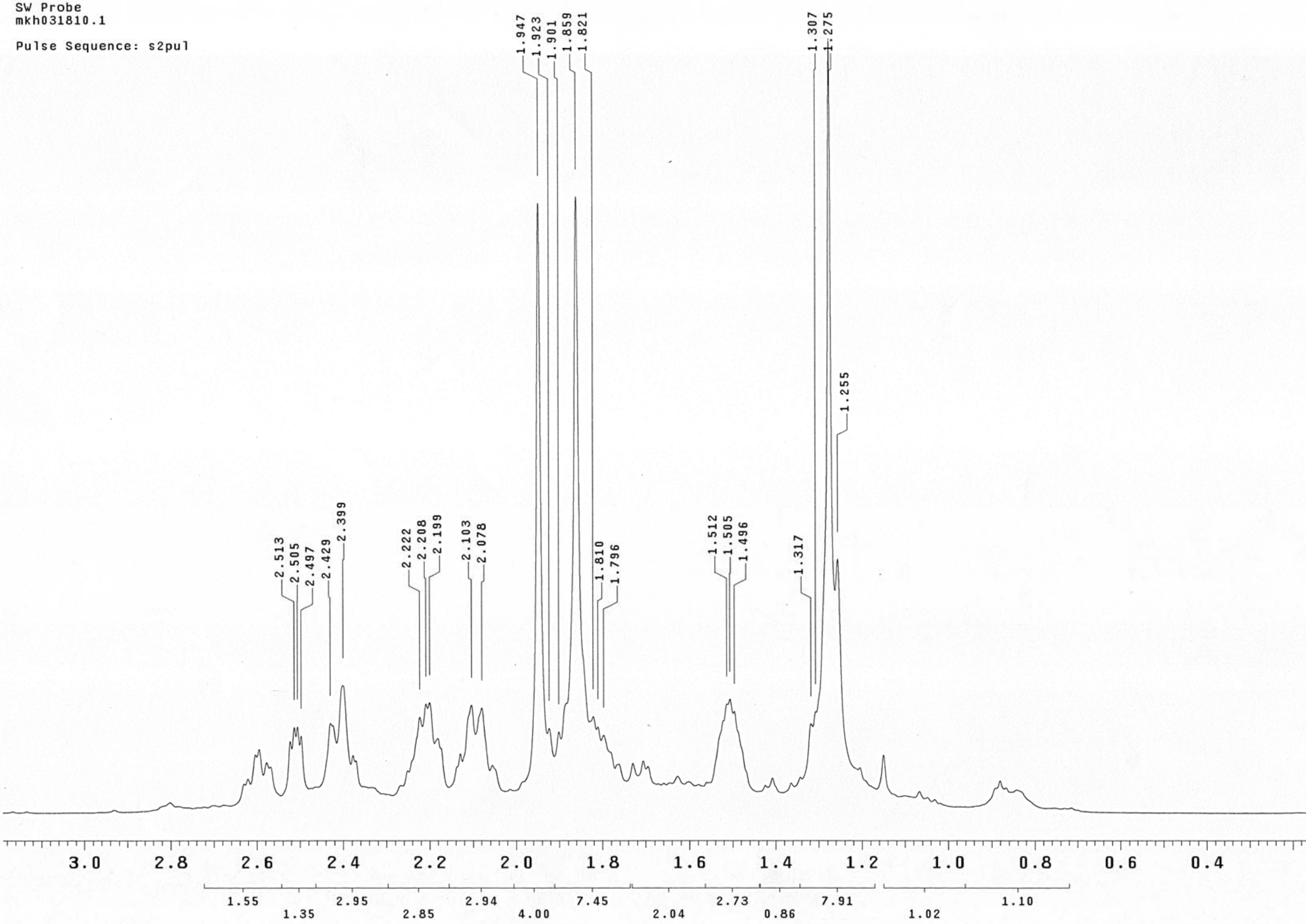

Re-3-5-55
in CDCl3
SW Probe
mkh031810.1

Pulse Sequence: s2pul

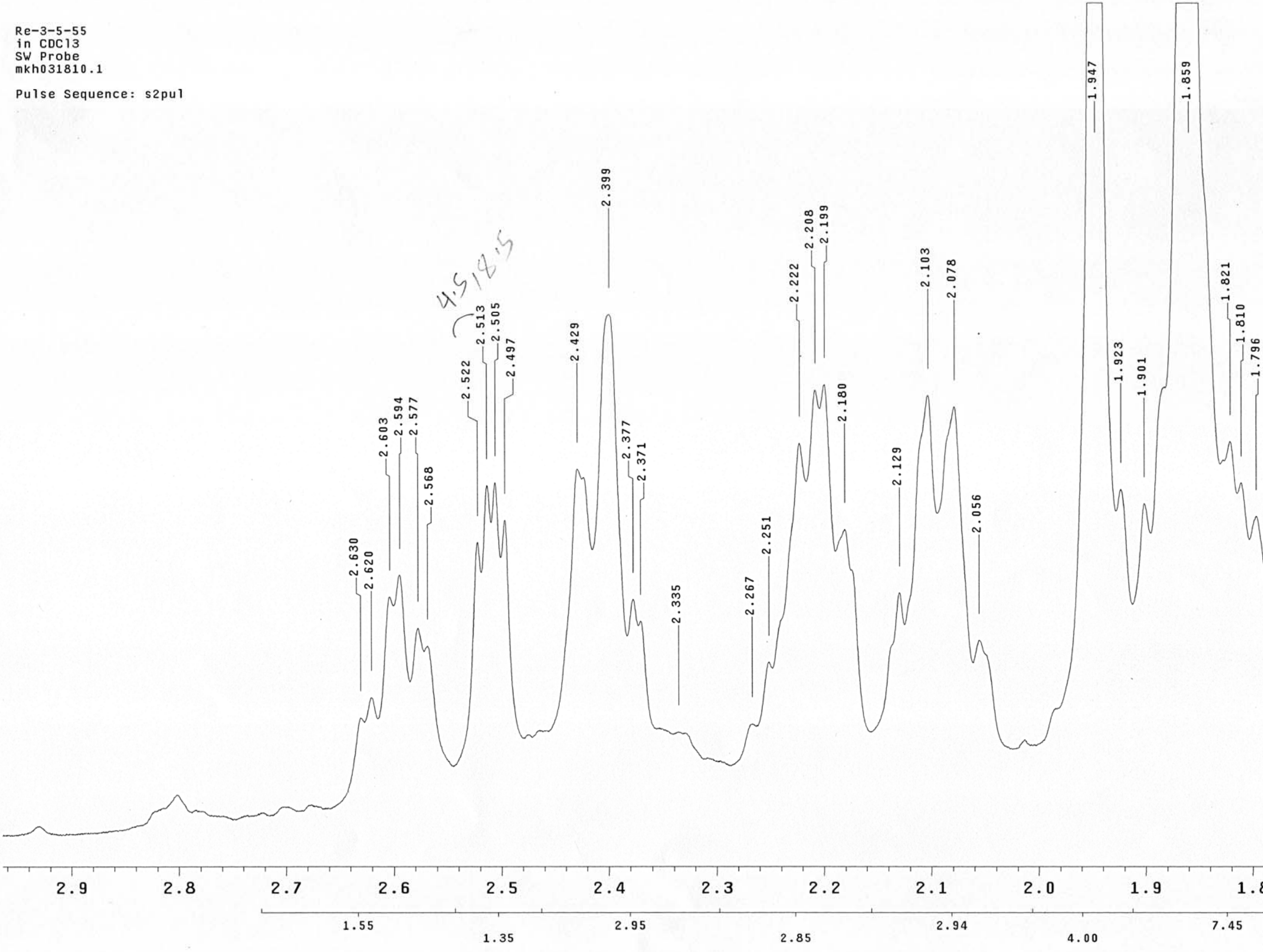

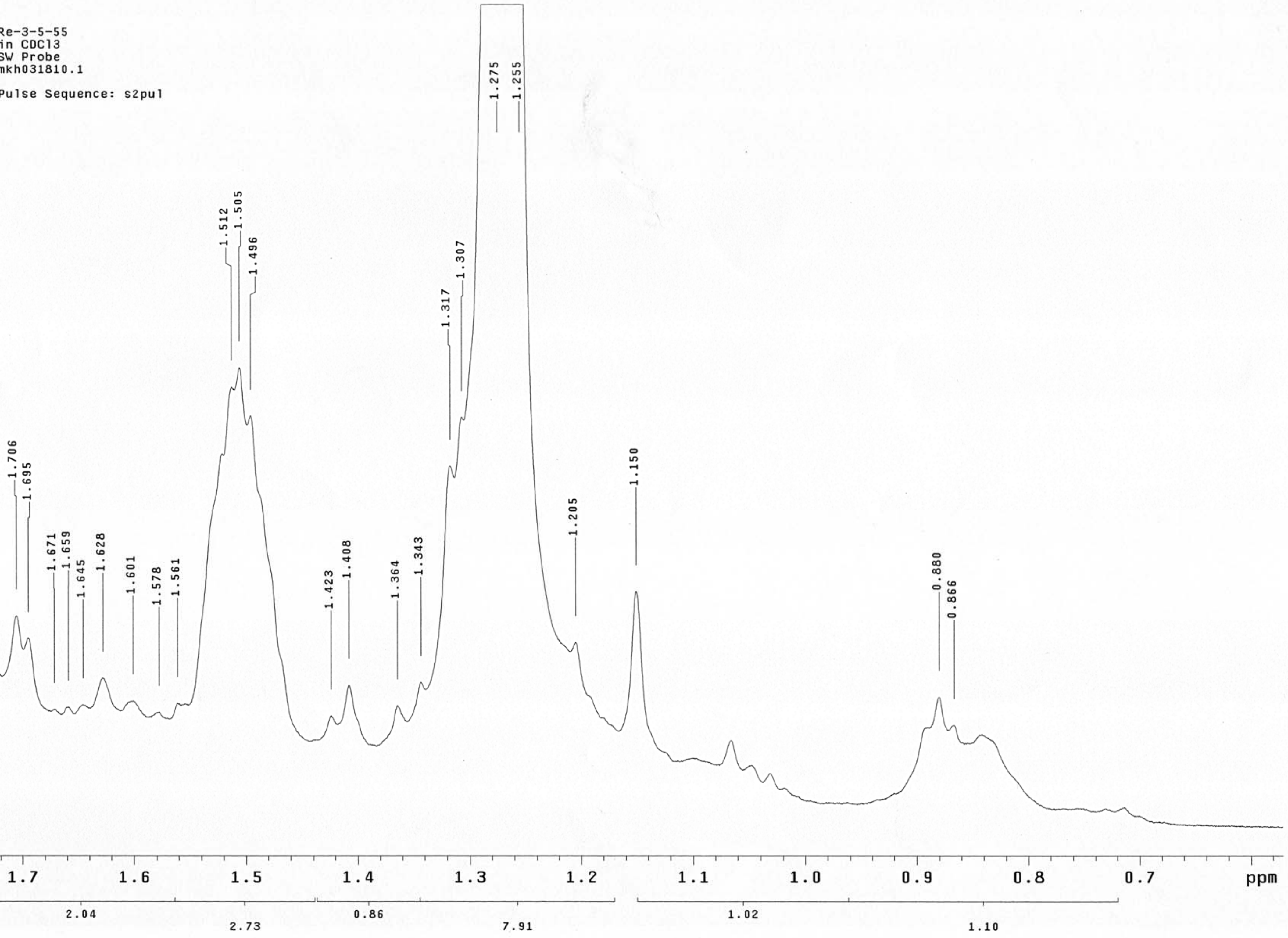
Re-3-5-55
in CDCl3
SW Probe
mkh031810.1
Pulse Sequence: s2pul
1.706
1.695
1.671
1.659
1.645
1.628
1.601
1.578
1.561
1.512
1.505
1.496
1.423
1.408
1.364
1.343
1.317
1.307
1.275
1.255
1.205
1.150
0.880
0.866
1.7
1.6
1.5
1.4
1.3
1.2
1.1
1.0
0.9
0.8
0.7
ppm
2.04
2.73
0.86
7.91
1.02
1.10

Re-3-5-55
in CDCl3
SW Probe
mkc031810.1

Pulse Sequence: s2pul

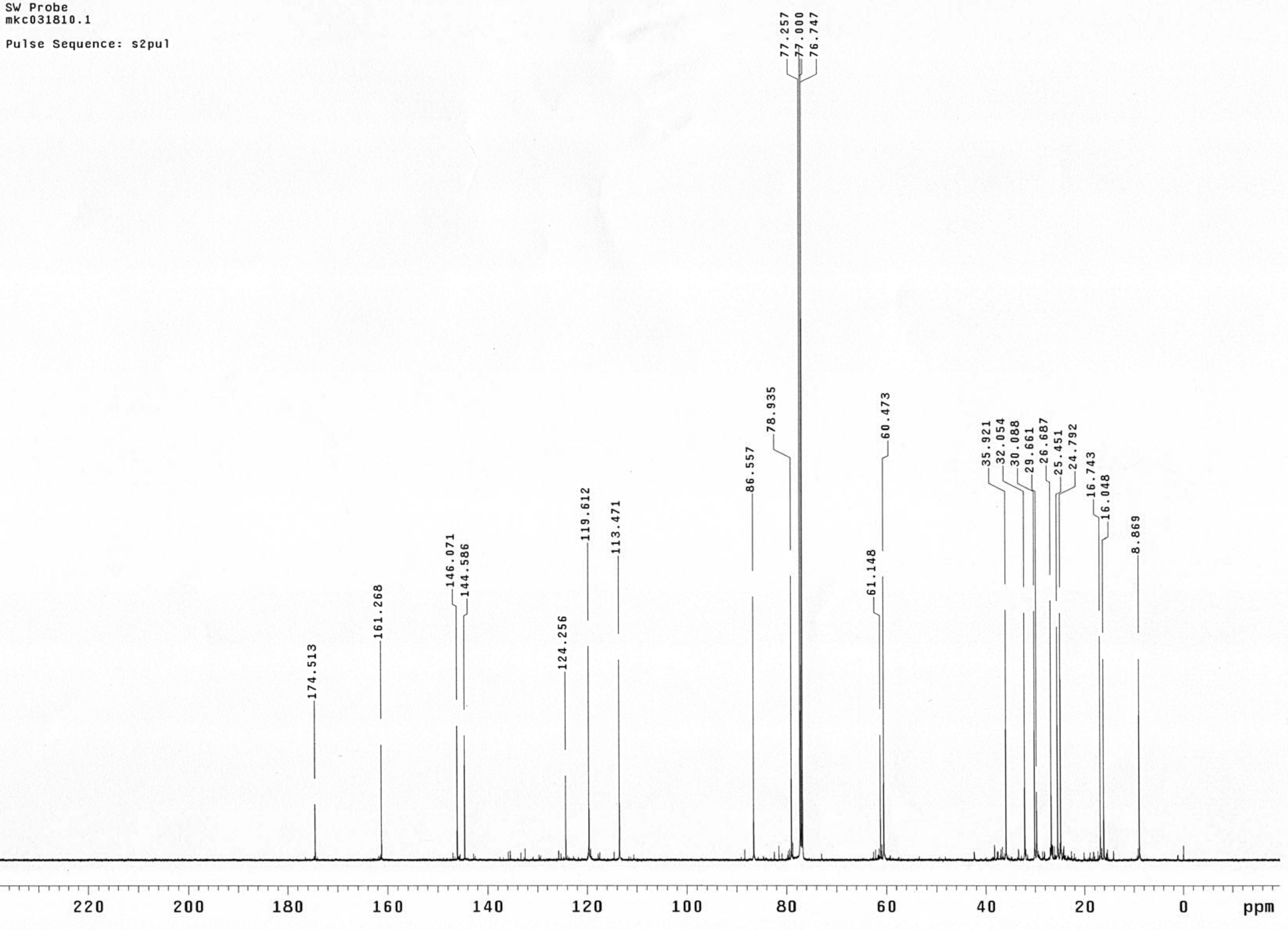

Re-3-5-55
in CDCl3
SW Probe
mkc031810.1

Pulse Sequence: s2pul

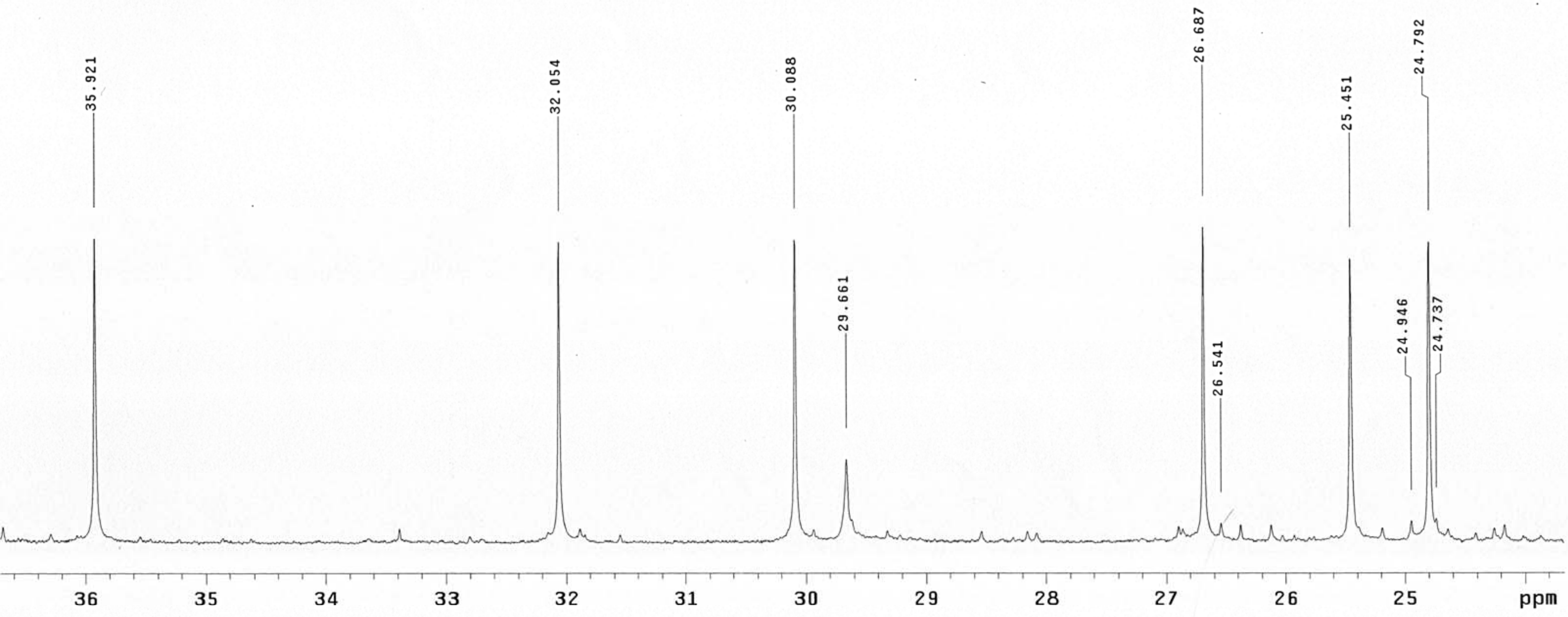

Re-3-5-55
Gradient COSY
in CDCl3
SW Probe
mkgcosy031810.1

Pulse Sequence: gCOSY

Solvent: CDCl3
Temp. 23.0 C / 296.1 K
INOVA-500 "inova500a"

Relax. delay 1.000 sec
Acq. time 0.745 sec
Width 5497.5 Hz
2D Width 5497.5 Hz
2 repetitions
256 increments
OBSERVE H1, 499.7081714 MHz
DATA PROCESSING
Sq. sine bell 0.093 sec
F1 DATA PROCESSING
Sq. sine bell 0.023 sec
FT size 8192 x 8192
Total time 15 min, 40 sec

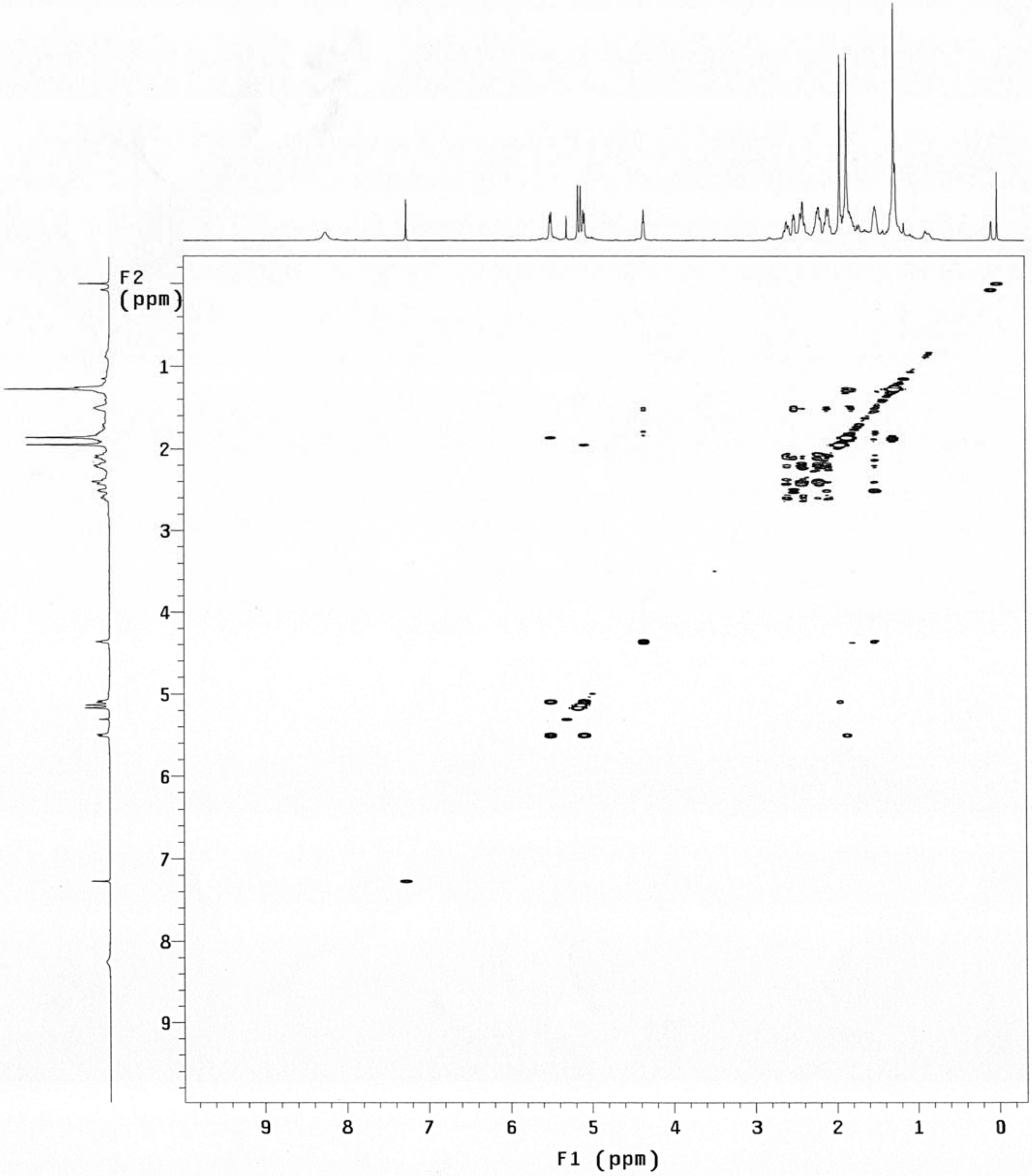

Re-3-5-55
Gradient HSQC
all peaks
in CDCl3
SW Probe
mkghsqc031810.1

Pulse Sequence: gHSQC

Solvent: CDCl3
Temp. 23.0 C / 296.1 K
User: 1-14-87
INOVA-500 "inova500a"

Relax. delay 1.000 sec
Acq. time 0.186 sec
Width 5497.5 Hz
2D Width 21361.8 Hz
24 repetitions
2 x 128 increments
OBSERVE H1, 499.7081714 MHz
DECOUPLE C13, 125.6611136 MHz
Power 43 dB
on during acquisition
off during delay
GARP-1 modulated
DATA PROCESSING
Gauss apodization 0.086 sec
F1 DATA PROCESSING
Gauss apodization 0.011 sec
FT size 2048 x 2048
Total time 2 hr, 9 min, 27 sec

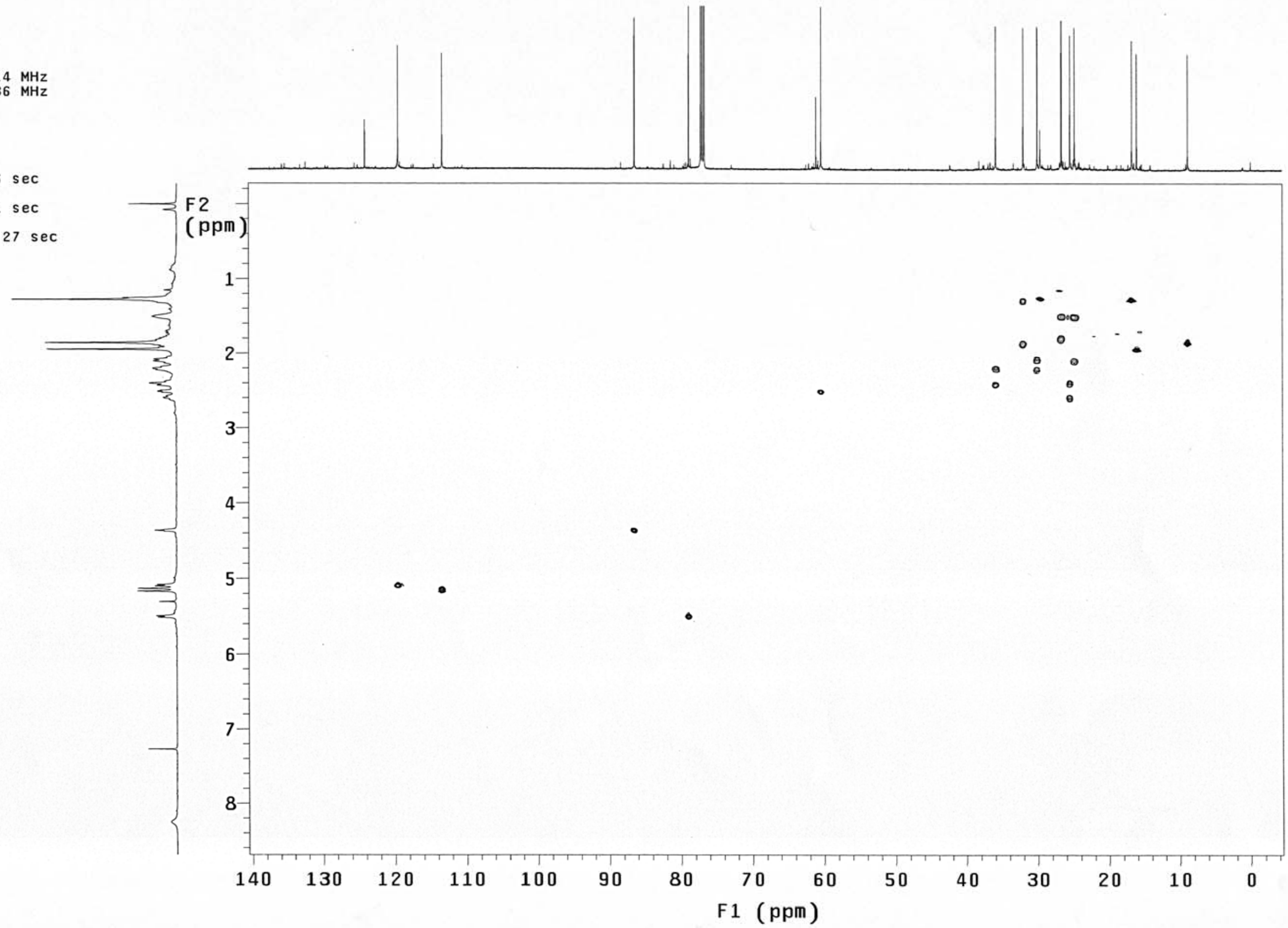

Re-3-5-55
Gradient HMBC
in CDCl3
SW Probe
mkghmbc031810.1

Pulse Sequence: gHMBC

Solvent: CDCl3
Temp. 23.0 C / 296.1 K
User: 1-14-87
INOVA-500 "inova500a"

Relax. delay 1.000 sec
Acq. time 0.186 sec
Width 5497.5 Hz
2D Width 30154.5 Hz
96 repetitions
400 increments
OBSERVE H1, 499.7081714 MHz
DATA PROCESSING
Sine bell 0.093 sec
F1 DATA PROCESSING
Sine bell 0.007 sec
FT size 2048 x 2048
Total time 13 hr, 36 min, 26 sec

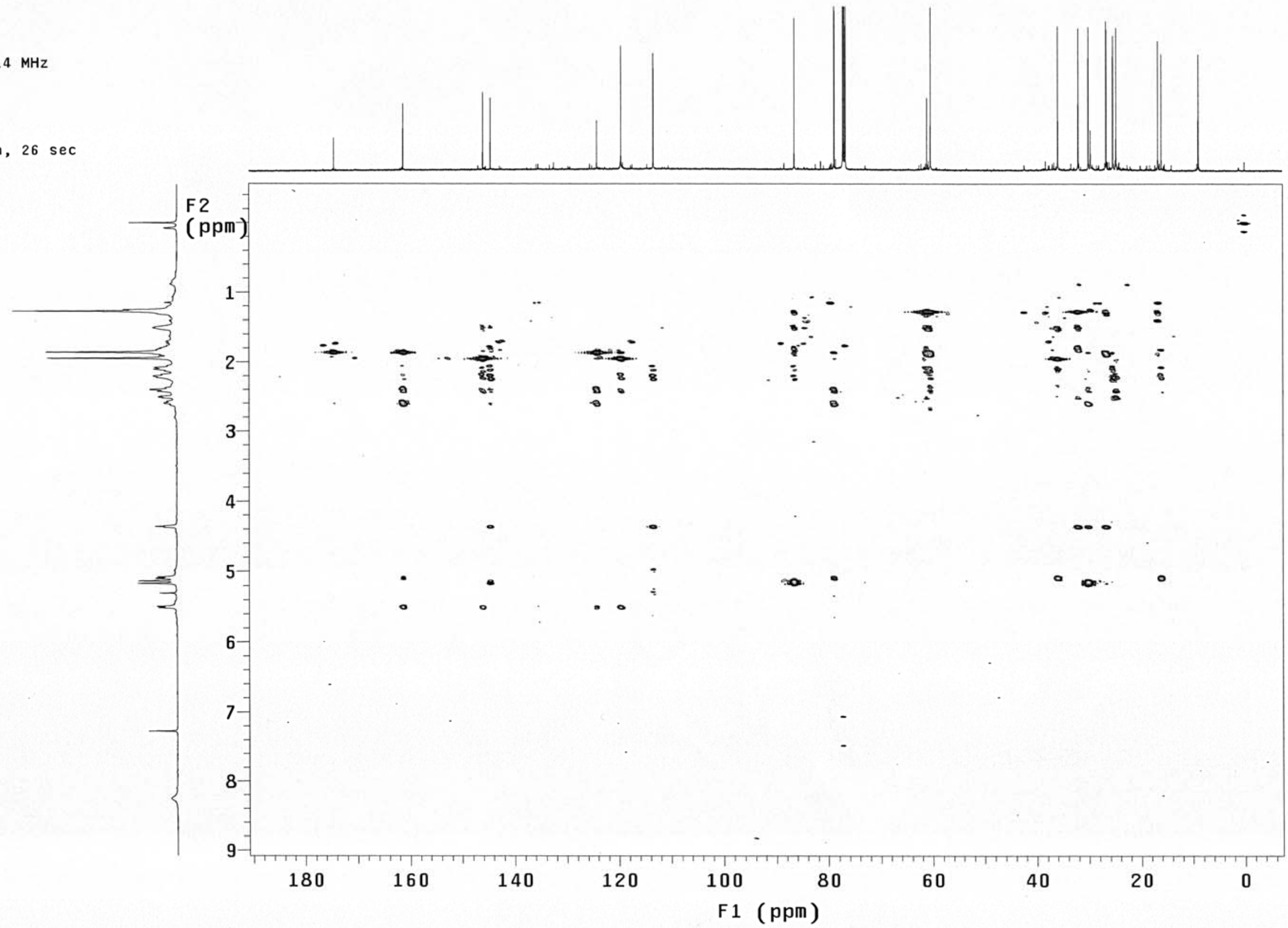

Re-3-5-55
Gradient HMBC
in CDCl3
SW Probe
mkghmbc031810.1

Pulse Sequence: gHMBC

Solvent: CDCl3
Temp. 23.0 C / 296.1 K
User: 1-14-87
INOVA-500 "inova500a"

Relax. delay 1.000 sec
Acq. time 0.186 sec
Width 5497.5 Hz
2D Width 30154.5 Hz
96 repetitions
400 increments
OBSERVE H1, 499.7081714 MHz
DATA PROCESSING
Sine bell 0.093 sec
F1 DATA PROCESSING
Sine bell 0.007 sec
FT size 2048 x 2048
Total time 13 hr, 36 min, 26 sec

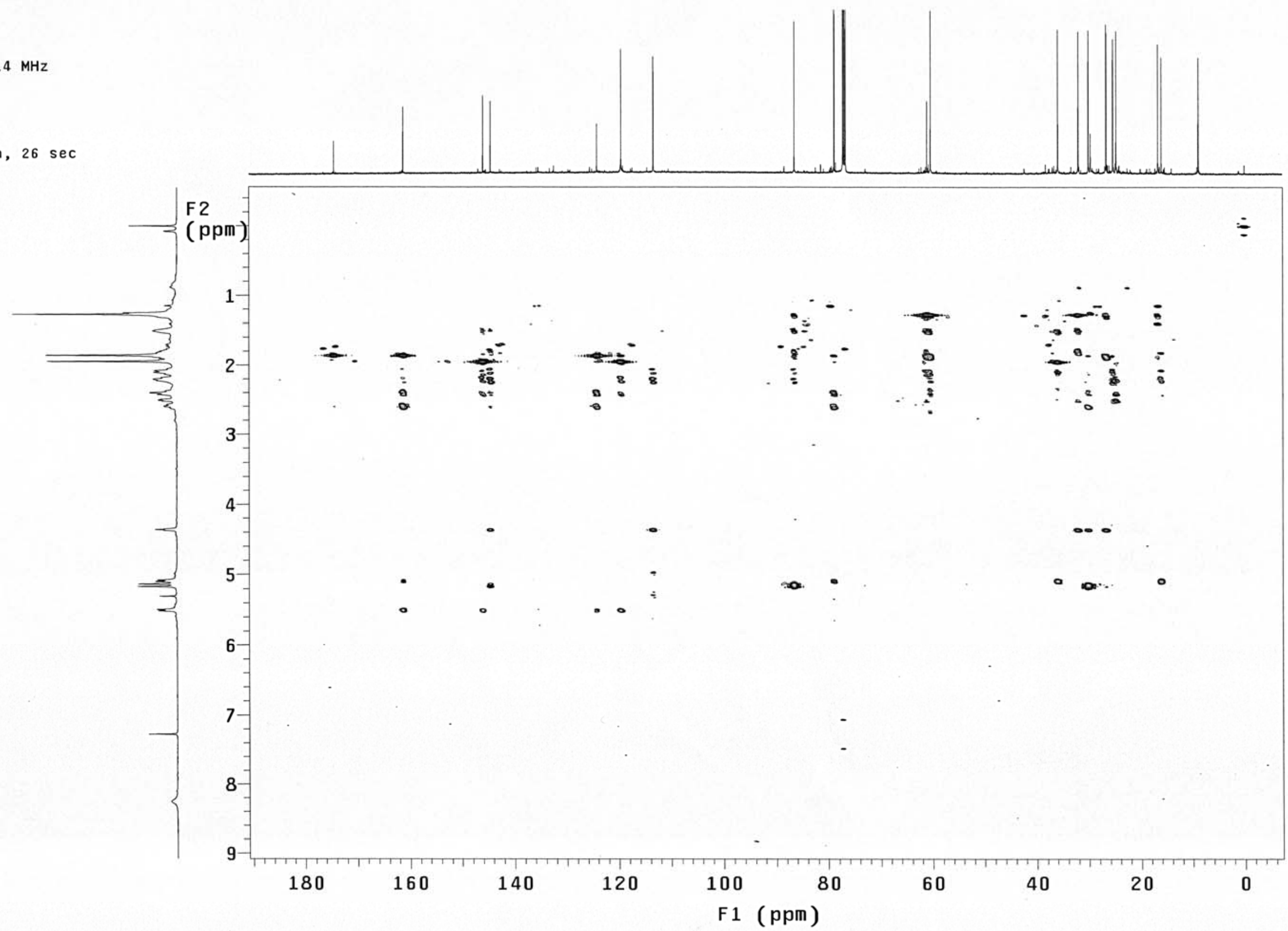

Re-3-5-55
NOESY
all peaks
d1=1 mix=1 sec
in CDCl3
SW Probe
mknoesy031810.1

Pulse Sequence: NOESY

Solvent: CDCl3
Temp. 23.0 C / 296.1 K
INOVA-500 "inova500a"

Relax. delay 3.000 sec
Mixing 1.000 sec
Acq. time 0.745 sec
Width 5497.5 Hz
2D Width 5497.5 Hz
32 repetitions
2 x 256 increments
OBSERVE H1, 499.7081714 MHz
DATA PROCESSING
Gauss apodization 0.086 sec
F1 DATA PROCESSING
Gauss apodization 0.034 sec
FT size 8192 x 8192
Total time 21 hr, 48 min, 20 sec

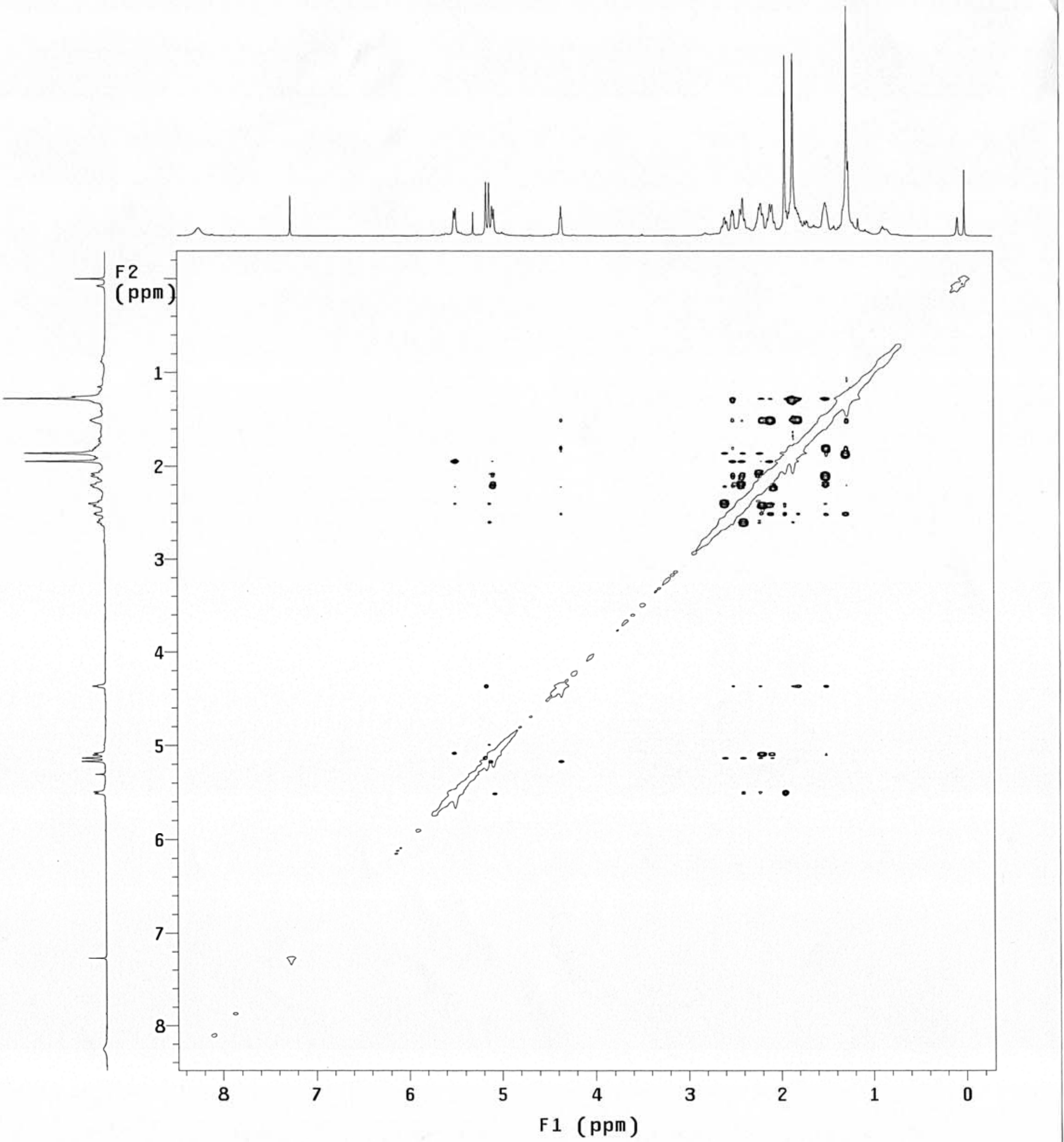

Re-3-5-55
NOESY
all peaks
d1=1 mix=1 sec
in CDCl3
SW Probe
mknoesy031810.1

Pulse Sequence: NOESY

Solvent: CDCl3
Temp. 23.0 C / 296.1 K
INOVA-500 "inova500a"

Relax. delay 3.000 sec
Mixing 1.000 sec
Acq. time 0.745 sec
Width 5497.5 Hz
2D Width 5497.5 Hz
32 repetitions
2 x 256 increments
OBSERVE H1, 499.7081714 MHz
DATA PROCESSING
Gauss apodization 0.086 sec
F1 DATA PROCESSING
Gauss apodization 0.034 sec
FT size 8192 x 8192
Total time 21 hr, 48 min, 20 sec

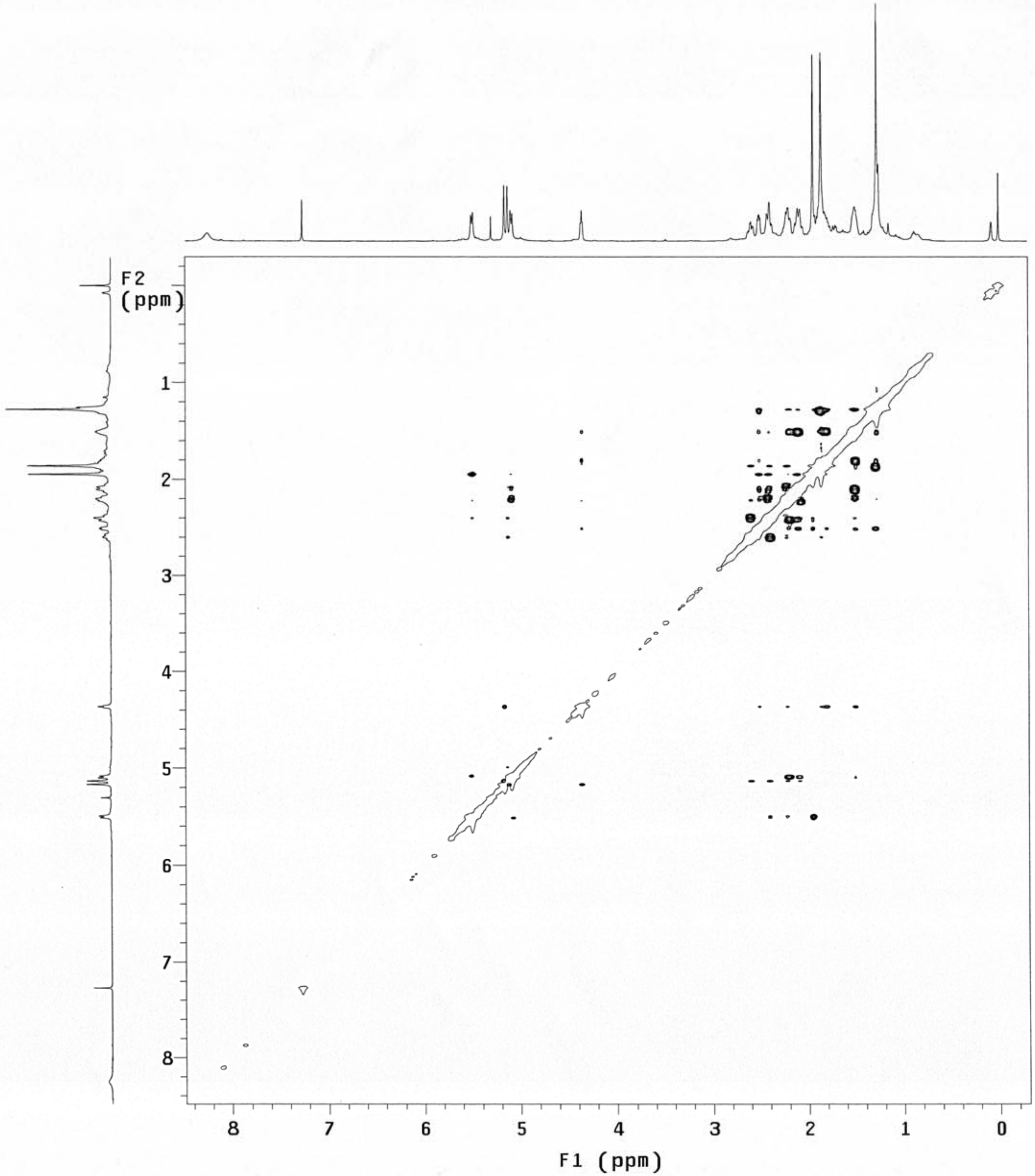

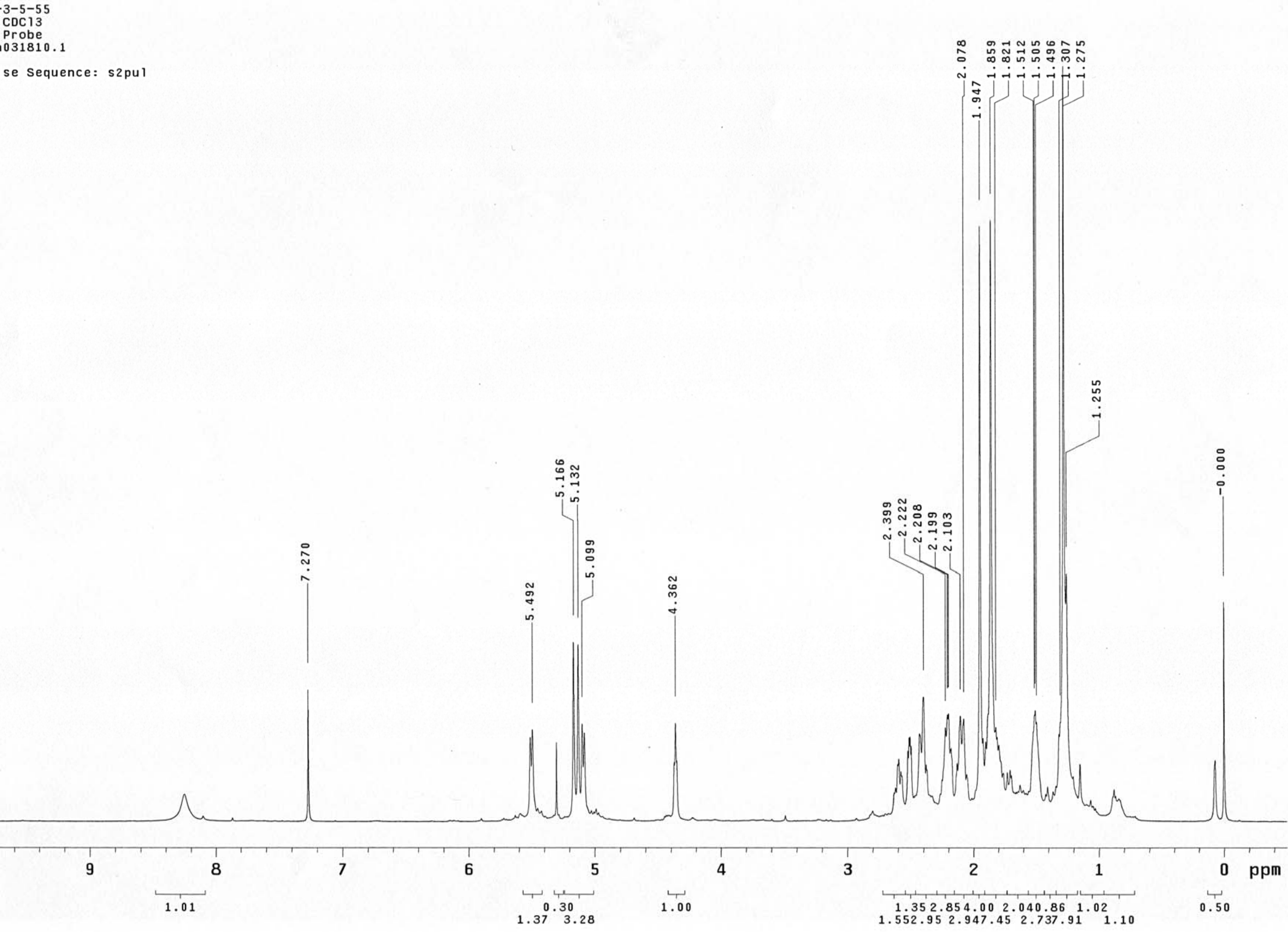

-3-5-55
CDCl3
Probe
031810.1
se Sequence: s2pul
2.078
1.947
1.859
1.821
1.512
1.505
1.496
1.307
1.275
1.255
-0.000
7.270
5.492
5.166
5.132
5.099
4.362
2.399
2.222
2.208
2.199
2.103
9
8
7
6
5
4
3
2
1
0
ppm
1.01
1.37
0.30
3.28
1.00
1.55
1.35
2.95
2.85
2.94
4.00
7.45
2.04
2.73
0.86
7.91
1.02
1.10
0.50

Re-3-55-8
Gradient HMBC
in CDCl3
SW Probe
mkghmbc040710.1

Pulse Sequence: gHMBC

Solvent: CDCl3
Temp. 23.0 C / 296.1 K
User: 1-14-87
INOVA-500 "inova500a"

Relax. delay 1.000 sec
Acq. time 0.205 sec
Width 5006.3 Hz
2D Width 30154.5 Hz
32 repetitions
400 increments
OBSERVE H1, 499.7081720 MHz
DATA PROCESSING
Sine bell 0.102 sec
F1 DATA PROCESSING
Sine bell 0.007 sec
FT size 2048 x 2048
Total time 4 hr, 36 min, 30 sec

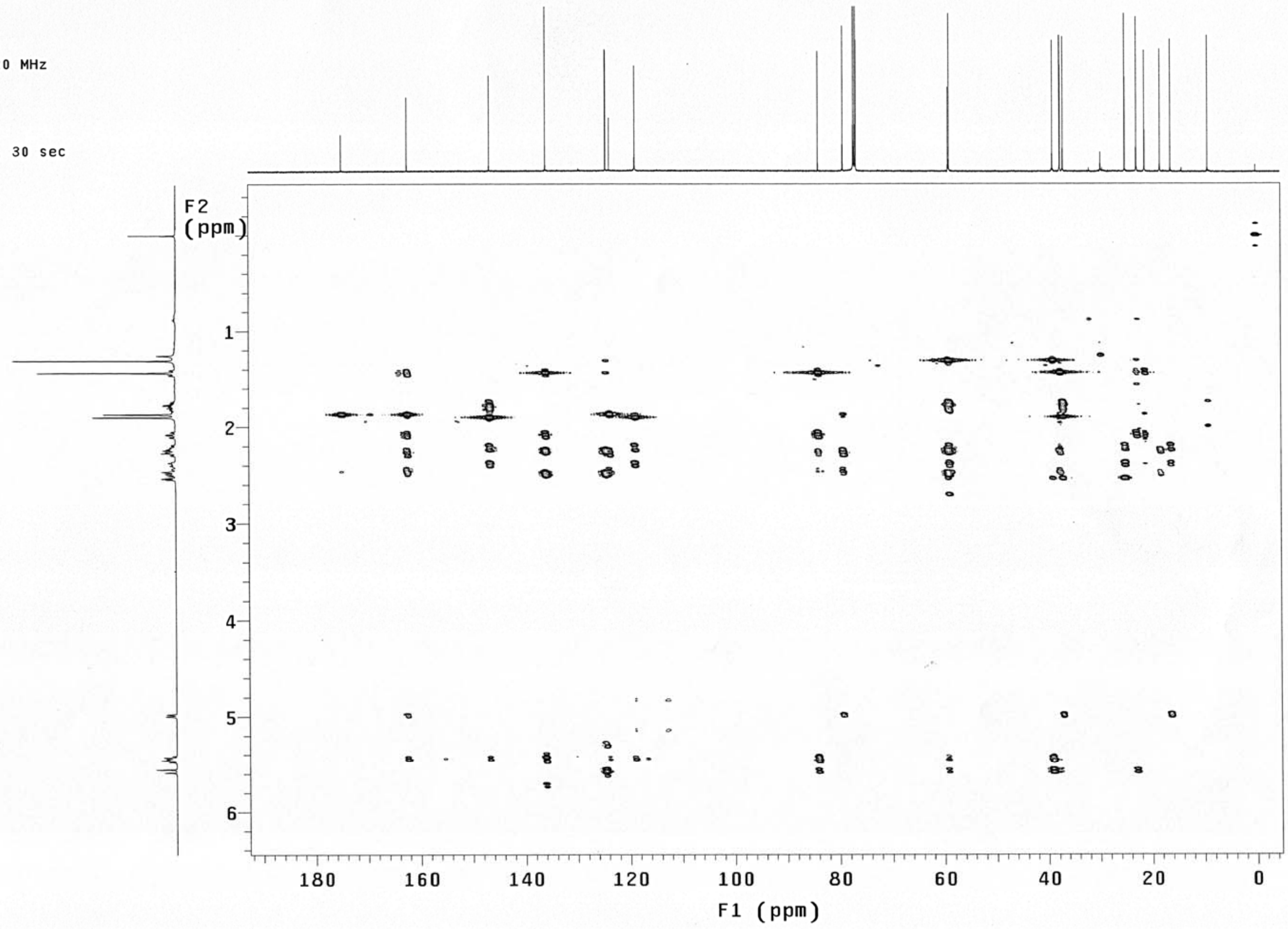

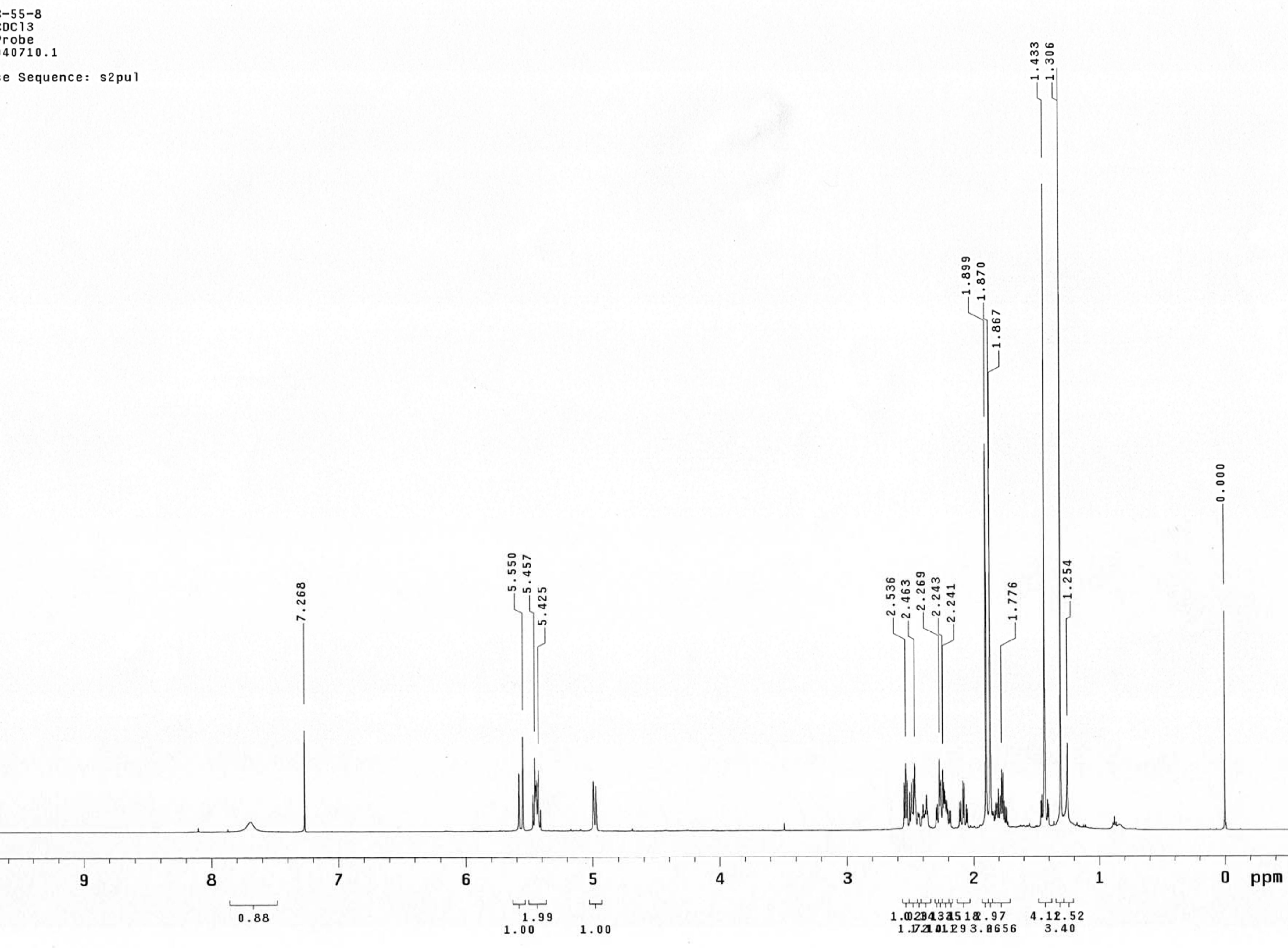

3-55-8
CDCl3
Probe
040710.1
se Sequence: s2pul
1.433
1.306
1.899
1.870
1.867
0.000
7.268
5.550
5.457
5.425
2.536
2.463
2.269
2.243
2.241
1.776
1.254
9
8
7
6
5
4
3
2
1
0
ppm
0.88
1.00
1.99
1.00
2.97
4.12
2.52
3.40

Re-3-55-8
in CDCl3
SW Probe
mkh040710.1

Pulse Sequence: s2pul

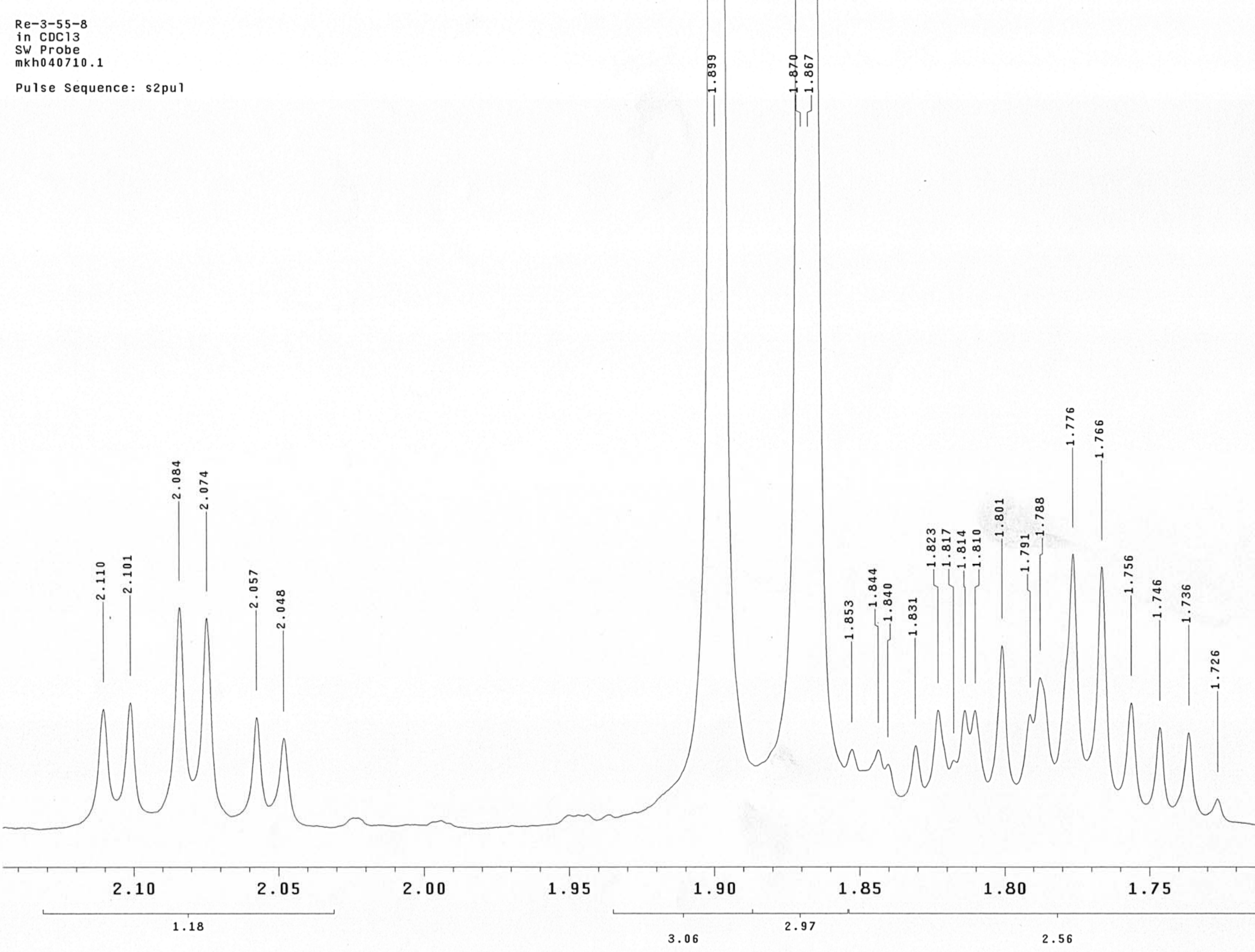

Re-3-55-8
in CDCl3
SW Probe
mkh040710.1

Pulse Sequence: s2pul

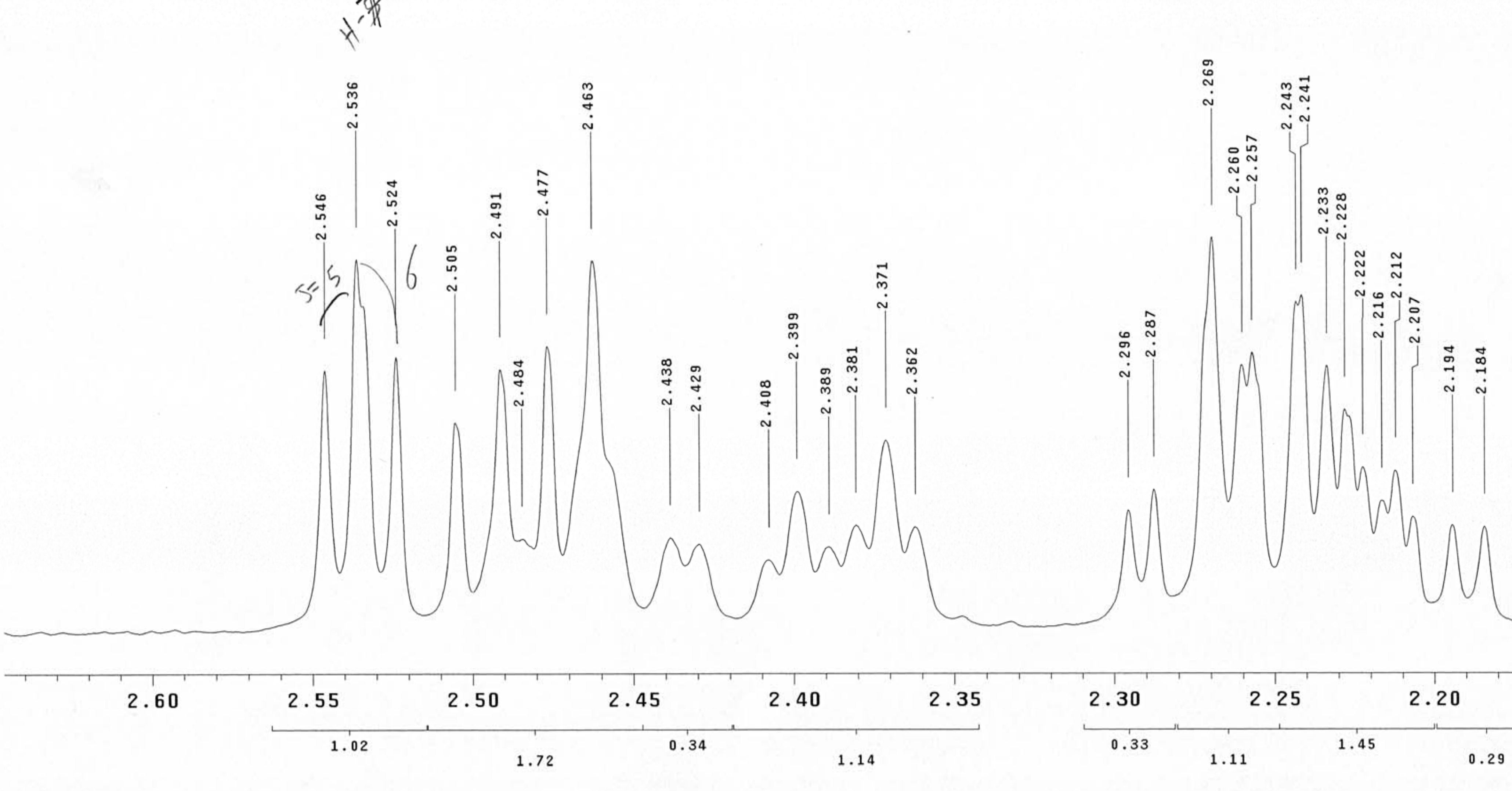

-3-55-8
CDCl3
Probe
h040710.1

lse Sequence: s2pul

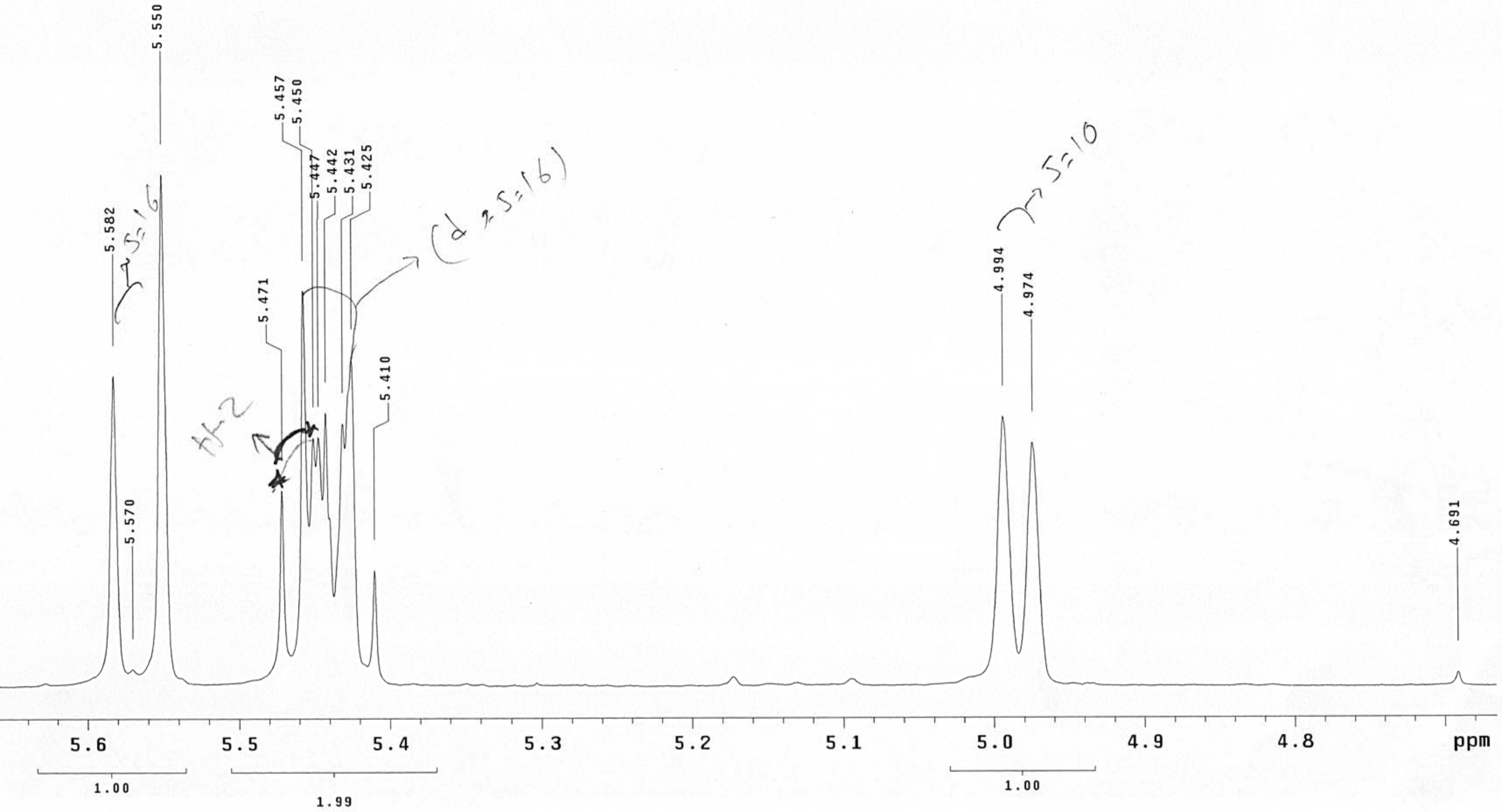

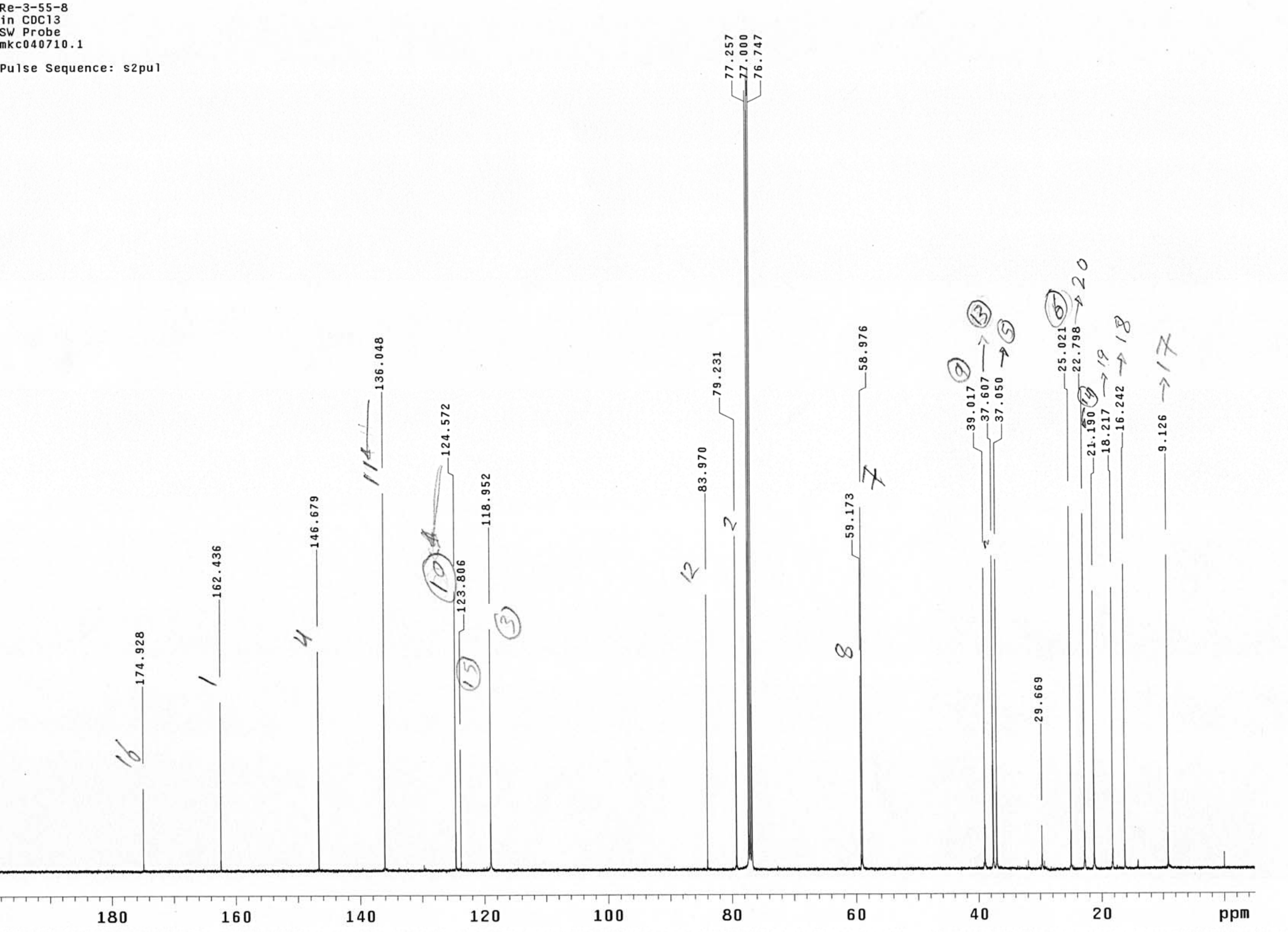
Re-3-55-8
in CDCl3
SW Probe
mkc040710.1
Pulse Sequence: s2pul
77.257
77.000
76.747
174.928
162.436
146.679
136.048
124.572
123.806
118.952
83.970
79.231
59.173
58.976
39.017
37.607
37.050
29.669
25.021
22.798
21.190
18.217
16.242
9.126
180
160
140
120
100
80
60
40
20
ppm

Re-3-55-8
in CDCl3
SW Probe
mkc040710.1

Pulse Sequence: s2pul

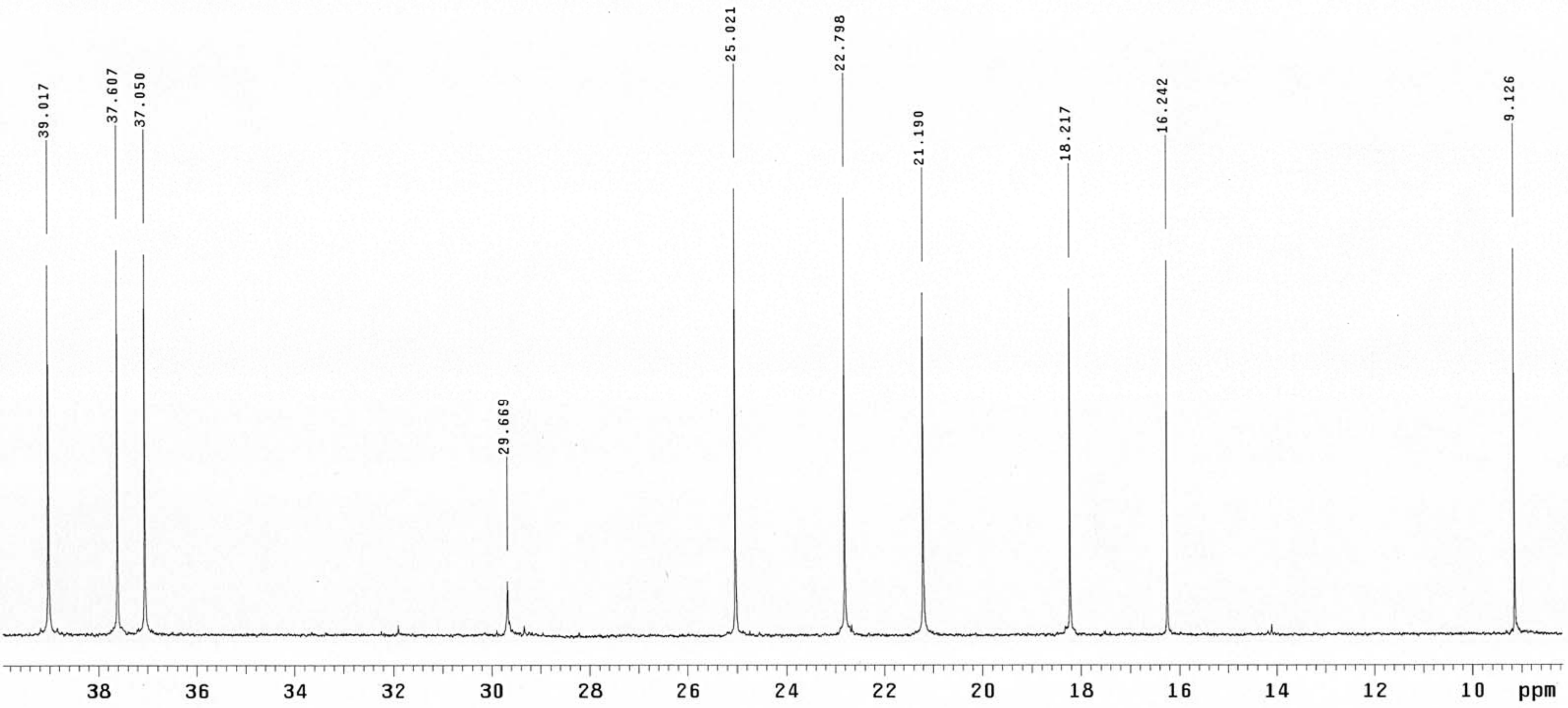

Re-3-55-8
Gradient COSY
in CDCl3
SW Probe
mkgcosy040710.1

Pulse Sequence: gCOSY

Solvent: CDCl3
Temp. 23.0 C / 296.1 K
INOVA-500 "inova500a"

Relax. delay 1.000 sec
Acq. time 0.818 sec
Width 5006.3 Hz
2D Width 5006.3 Hz
2 repetitions
256 increments
OBSERVE H1, 499.7081720 MHz
DATA PROCESSING
Sq. sine bell 0.102 sec
F1 DATA PROCESSING
Sq. sine bell 0.026 sec
FT size 8192 x 8192
Total time 16 min, 19 sec

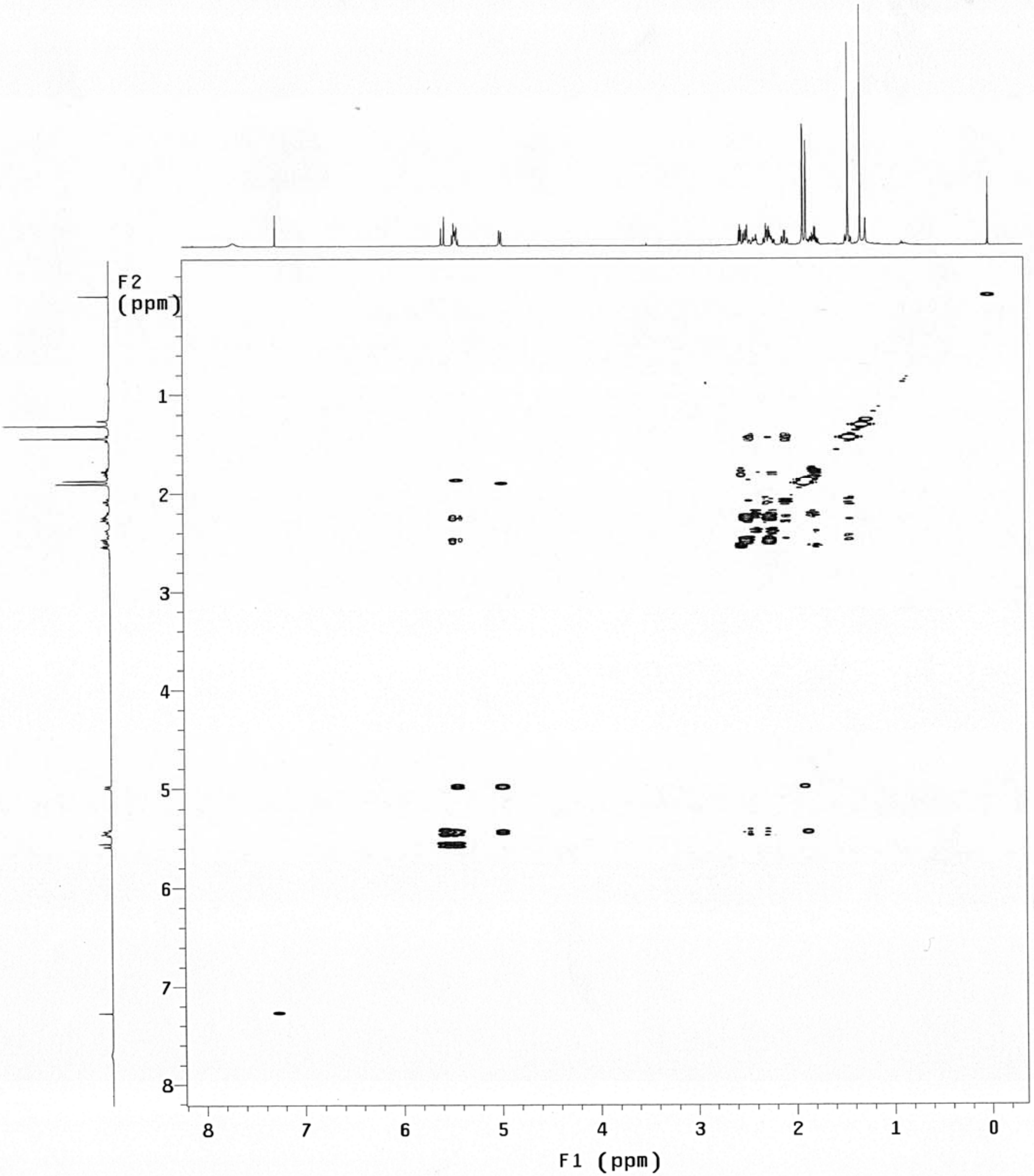

Re-3-55-8
Gradient HSQC
all peaks
in CDCl3
SW Probe
mkghsqc040710.1

Pulse Sequence: gHSQC

Solvent: CDCl3
Temp. 23.0 C / 296.1 K
User: 1-14-87
INOVA-500 "inova500a"

Relax. delay 1.000 sec
Acq. time 0.205 sec
Width 5006.3 Hz
2D Width 21361.8 Hz
24 repetitions
2 x 128 increments
OBSERVE H1, 499.7081720 MHz
DECOUPLE C13, 125.6611136 MHz
Power 43 dB
on during acquisition
off during delay
GARP-1 modulated
DATA PROCESSING
Gauss apodization 0.094 sec
F1 DATA PROCESSING
Gauss apodization 0.011 sec
FT size 2048 x 2048
Total time 2 hr, 11 min, 20 sec

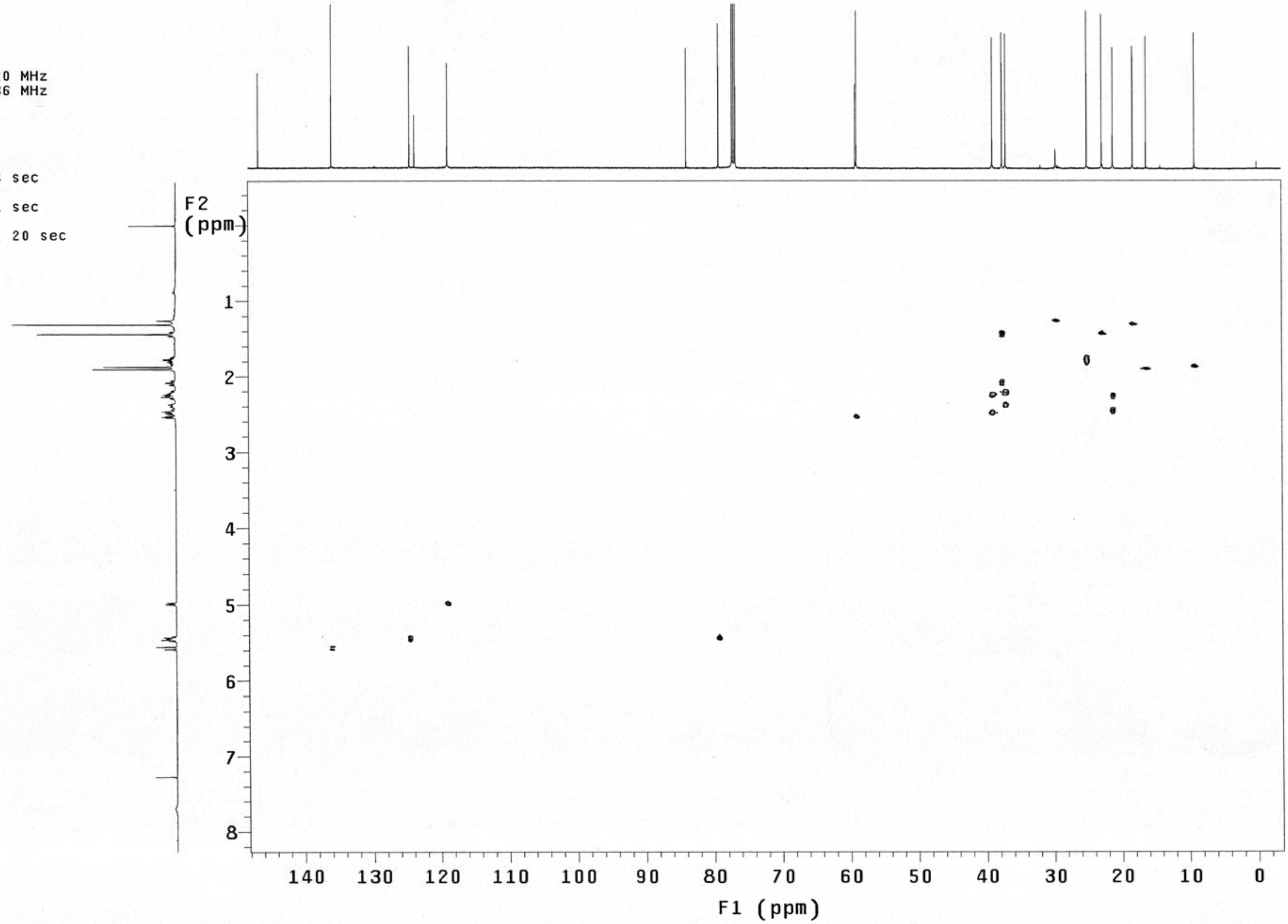

Re-3-55-8
Gradient HSQC
CH2 peaks only
in CDCl3
SW Probe
mkghsqc040710.1

Pulse Sequence: gHSQC

Solvent: CDCl3
Temp. 23.0 C / 296.1 K
User: 1-14-87
INOVA-500 "inova500a"

Relax. delay 1.000 sec
Acq. time 0.205 sec
Width 5006.3 Hz
2D Width 21361.8 Hz
24 repetitions
2 x 128 increments
OBSERVE H1, 499.7081720 MHz
DECOUPLE C13, 125.6611136 MHz
Power 43 dB
on during acquisition
off during delay
GARP-1 modulated
DATA PROCESSING
Gauss apodization 0.094 sec
F1 DATA PROCESSING
Gauss apodization 0.011 sec
FT size 2048 x 2048
Total time 2 hr, 11 min, 20 sec

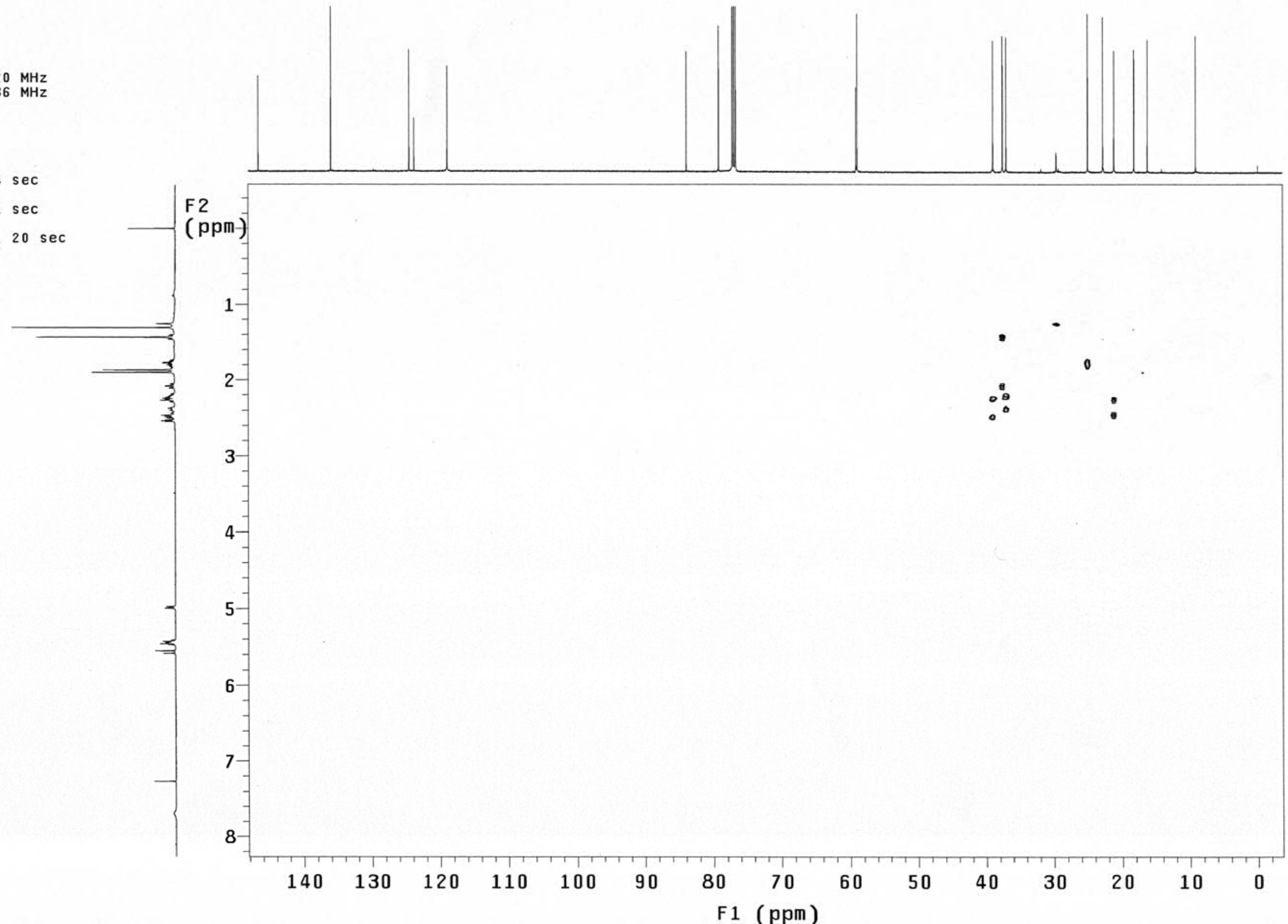

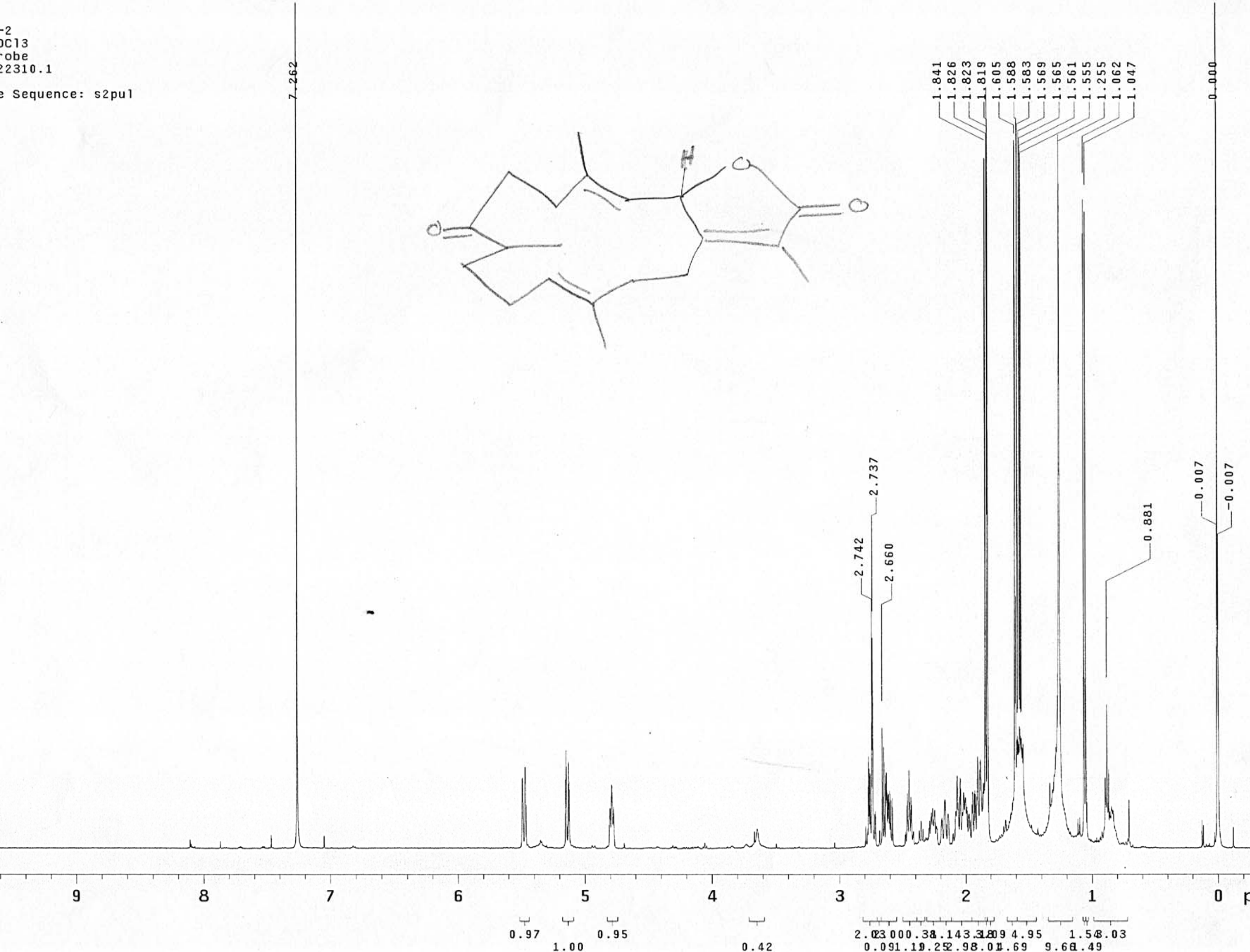
-2
DCl3
robe
22310.1
e Sequence: s2pul
7.262
1.841
1.826
1.823
1.819
1.605
1.588
1.583
1.569
1.565
1.561
1.555
1.255
1.062
1.047
0.000
2.737
2.742
2.660
0.881
0.007
-0.007
9
8
7
6
5
4
3
2
1
0
0.97
1.00
0.95
0.42
4.95
1.69

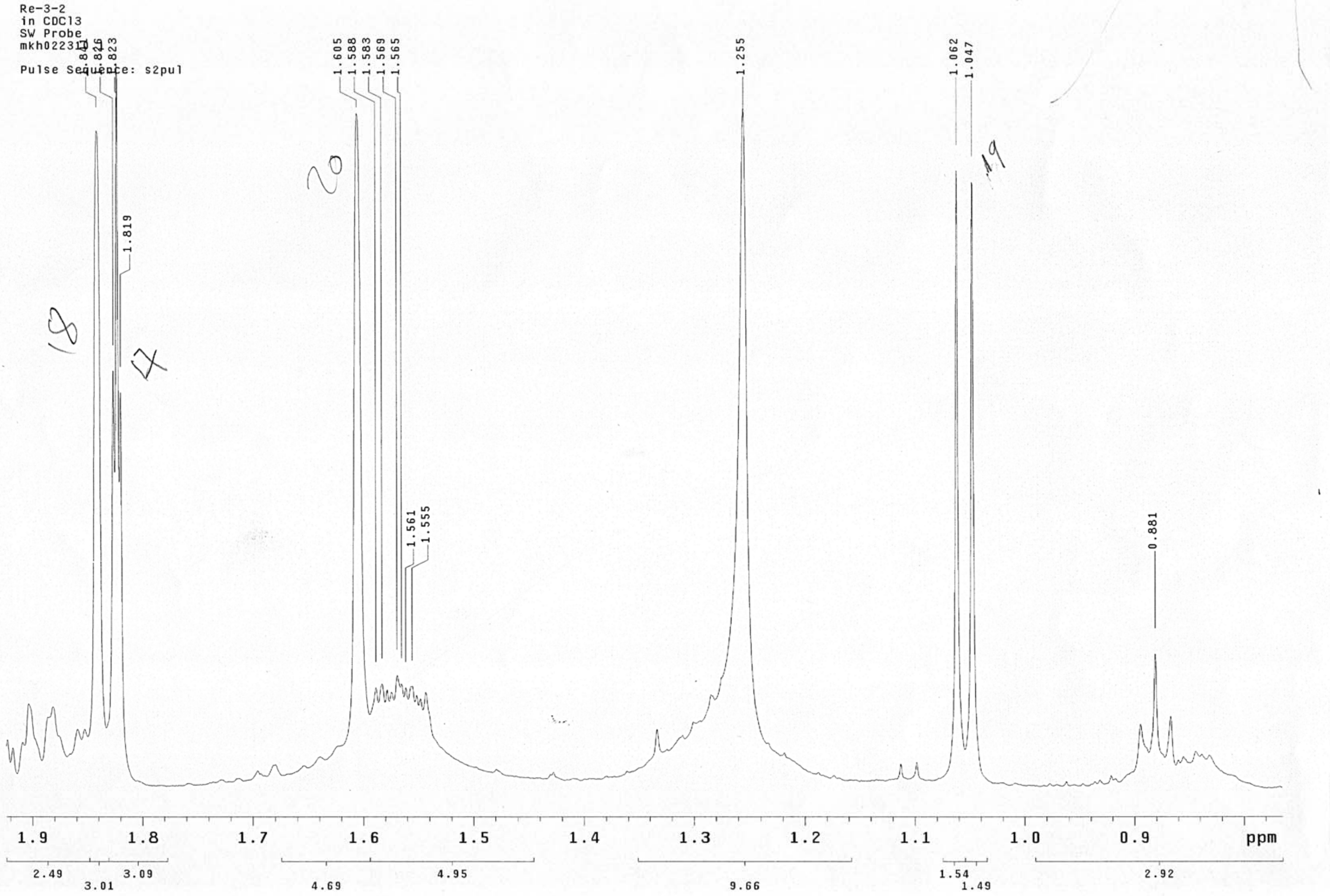
Re-3-2
in CDCl3
SW Probe
mkh022311
Pulse Sequence: s2pul
1.826
1.823
1.819
1.605
1.588
1.583
1.569
1.565
1.561
1.555
1.255
1.062
1.047
0.881
18
17
20
19
1.9
1.8
1.7
1.6
1.5
1.4
1.3
1.2
1.1
1.0
0.9
ppm
2.49
3.01
3.09
4.69
4.95
9.66
1.54
1.49
2.92

Re-3-2
in CDCl3
SW Probe
mkh022310.1

Pulse Sequence: s2pul

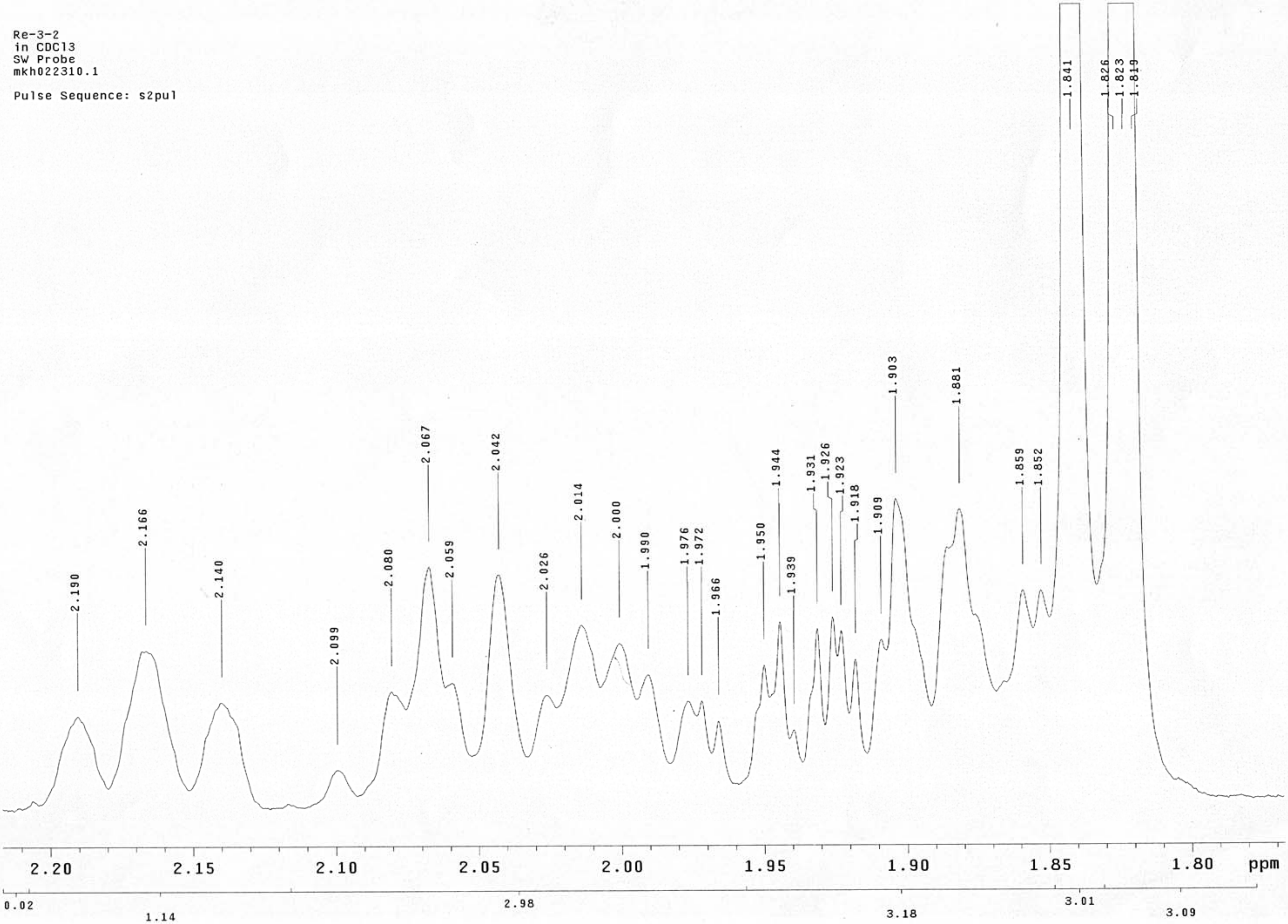

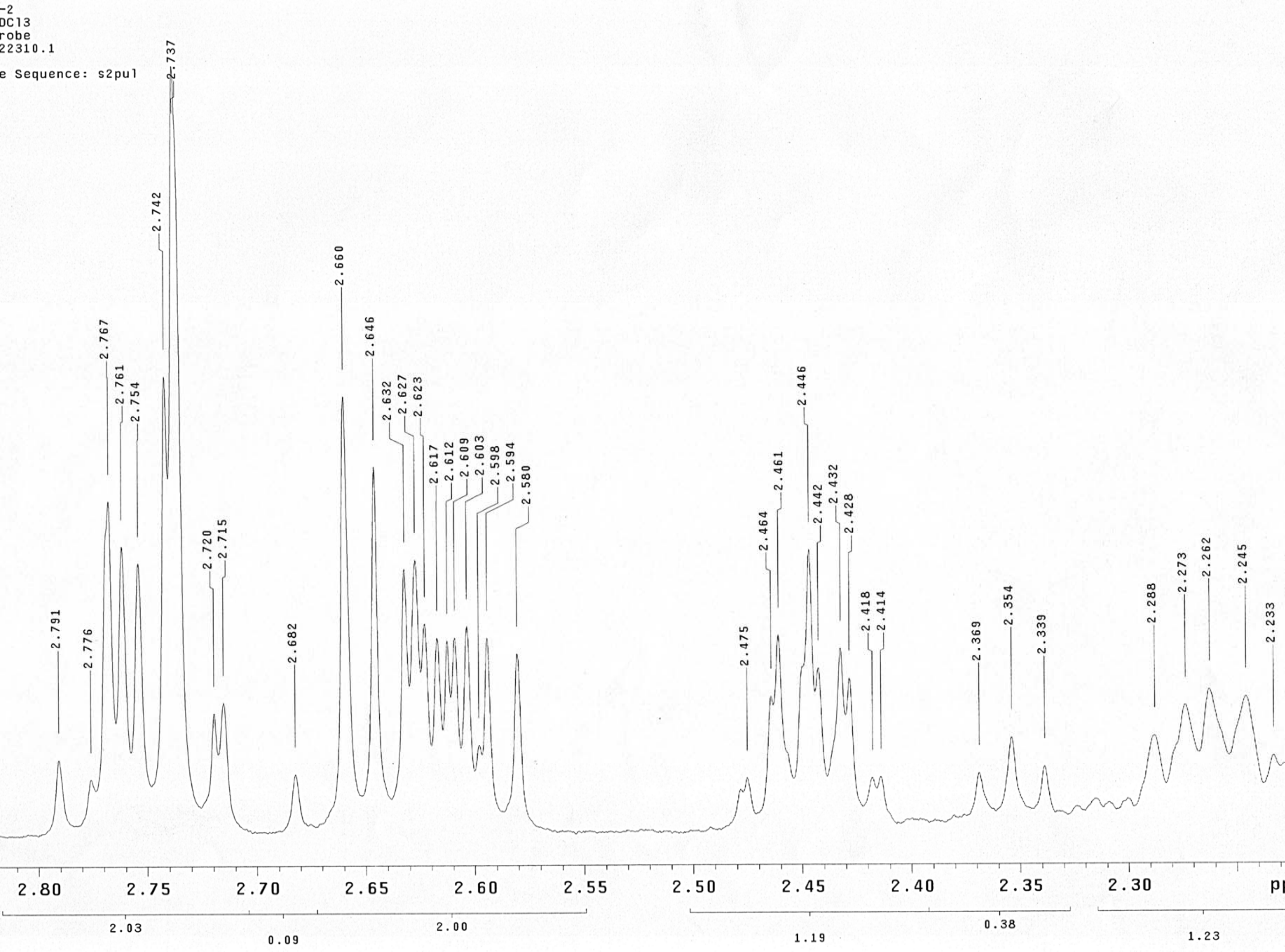

-2
DCl3
robe
22310.1
e Sequence: s2pul
2.791
2.776
2.767
2.761
2.754
2.742
2.737
2.720
2.715
2.682
2.660
2.646
2.632
2.627
2.623
2.617
2.612
2.609
2.603
2.598
2.594
2.580
2.475
2.464
2.461
2.446
2.442
2.432
2.428
2.418
2.414
2.369
2.354
2.339
2.288
2.273
2.262
2.245
2.233
2.80
2.75
2.70
2.65
2.60
2.55
2.50
2.45
2.40
2.35
2.30
pp
2.03
0.09
2.00
1.19
0.38
1.23

Re-3-2
in CDCl3
SW Probe
mkc022310.1

Pulse Sequence: s2pul

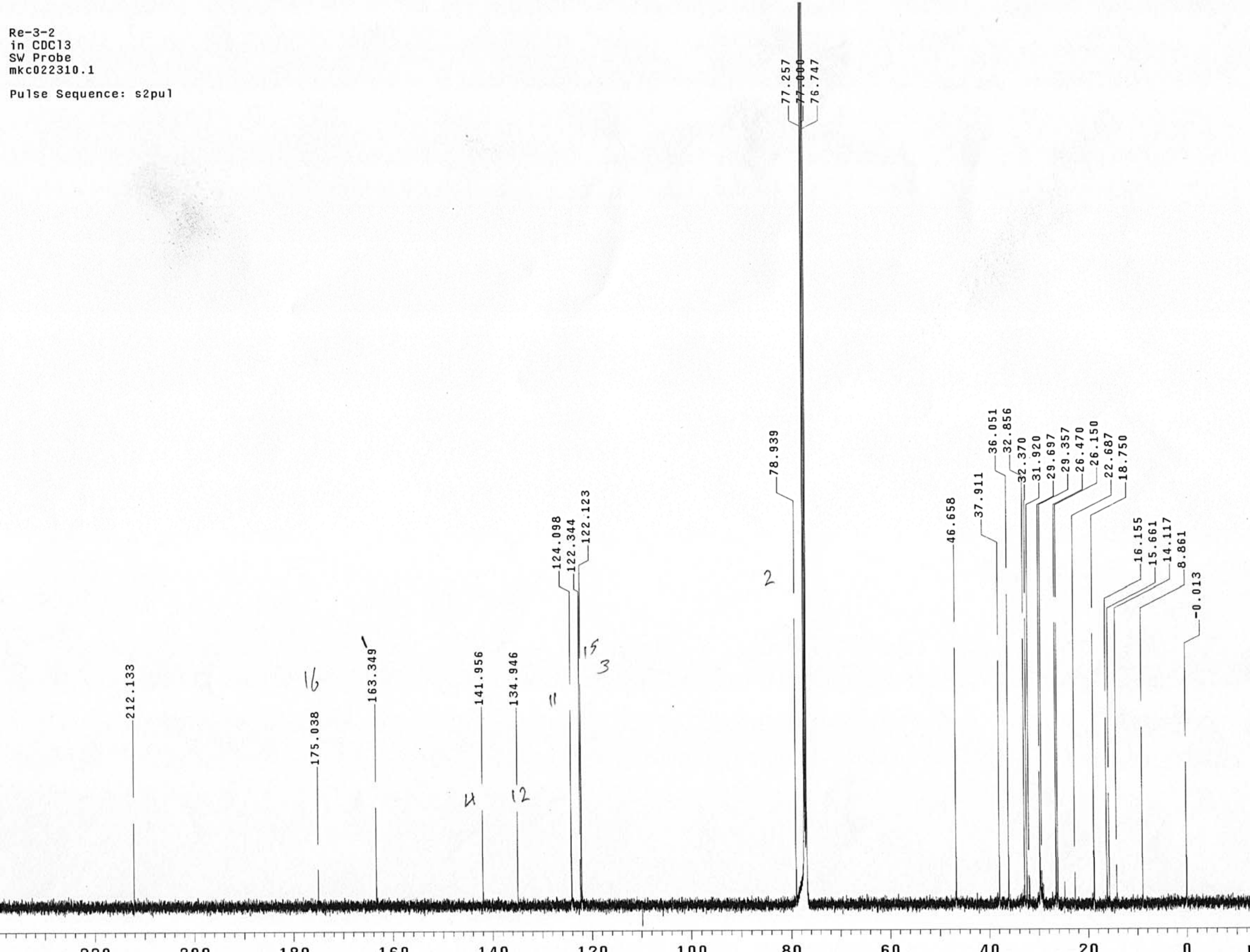

Re-3-2
in CDCl3
SW Probe
mkc022310.1

Pulse Sequence: s2pul

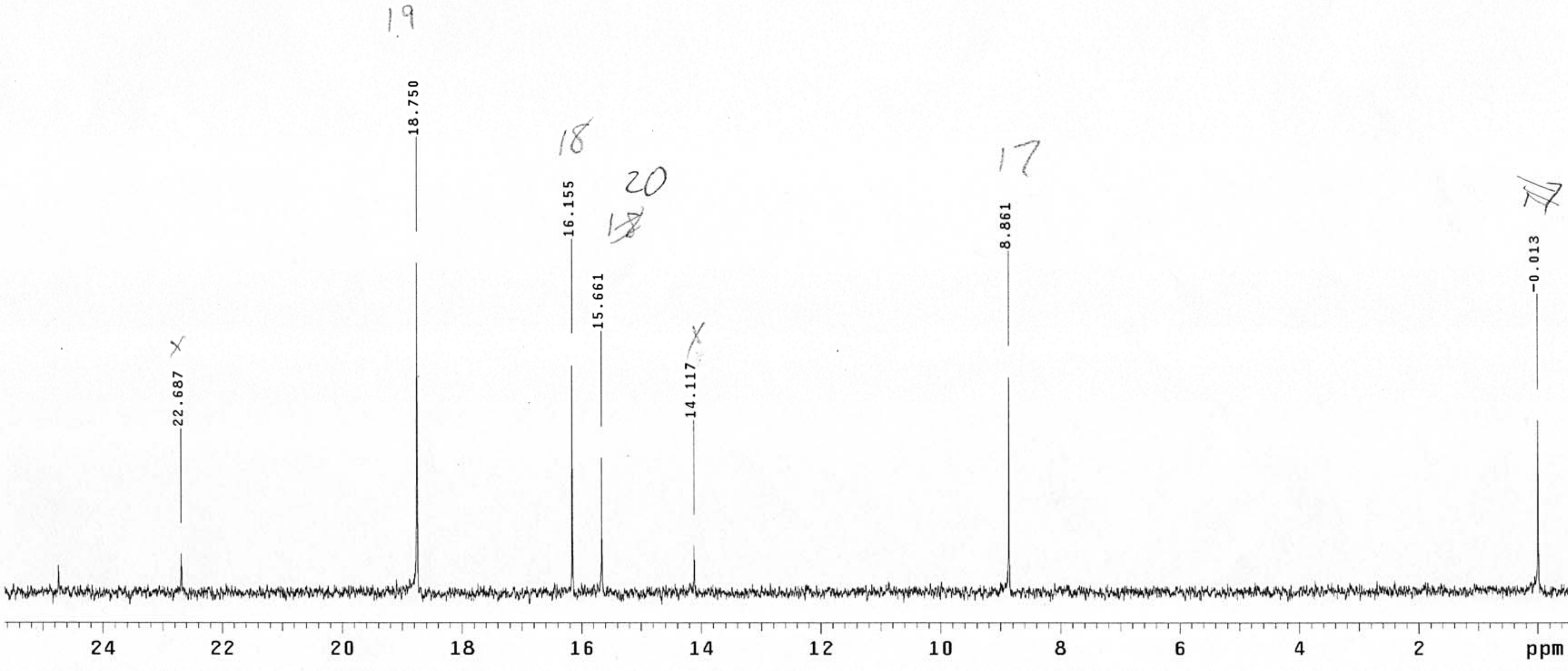

e-3-2
n CDCl3
W Probe
akc022310.1

Pulse Sequence: s2pul

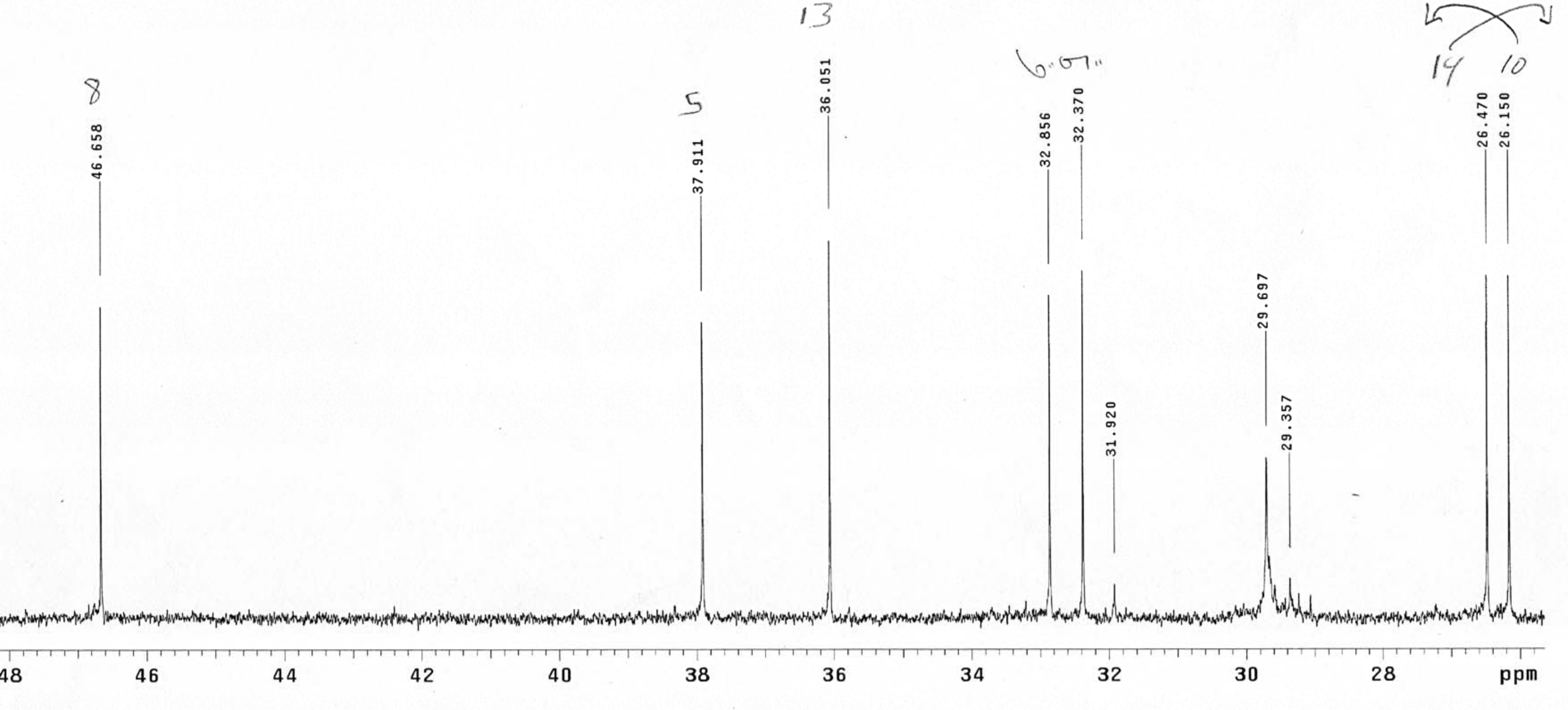

Re-3-2
Gradient COSY
in CDCl3
SW Probe
mkgcosy022310.1

Pulse Sequence: gCOSY

Solvent: CDCl3
Temp. 23.0 C / 296.1 K
INOVA-500 "inova500a"

Relax. delay 1.000 sec
Acq. time 0.818 sec
Width 5006.3 Hz
2D Width 5006.3 Hz
4 repetitions
256 increments
OBSERVE H1, 499.7081717 MHz
DATA PROCESSING
Sq. sine bell 0.102 sec
F1 DATA PROCESSING
Sq. sine bell 0.026 sec
FT size 8192 x 8192
Total time 32 min, 10 sec

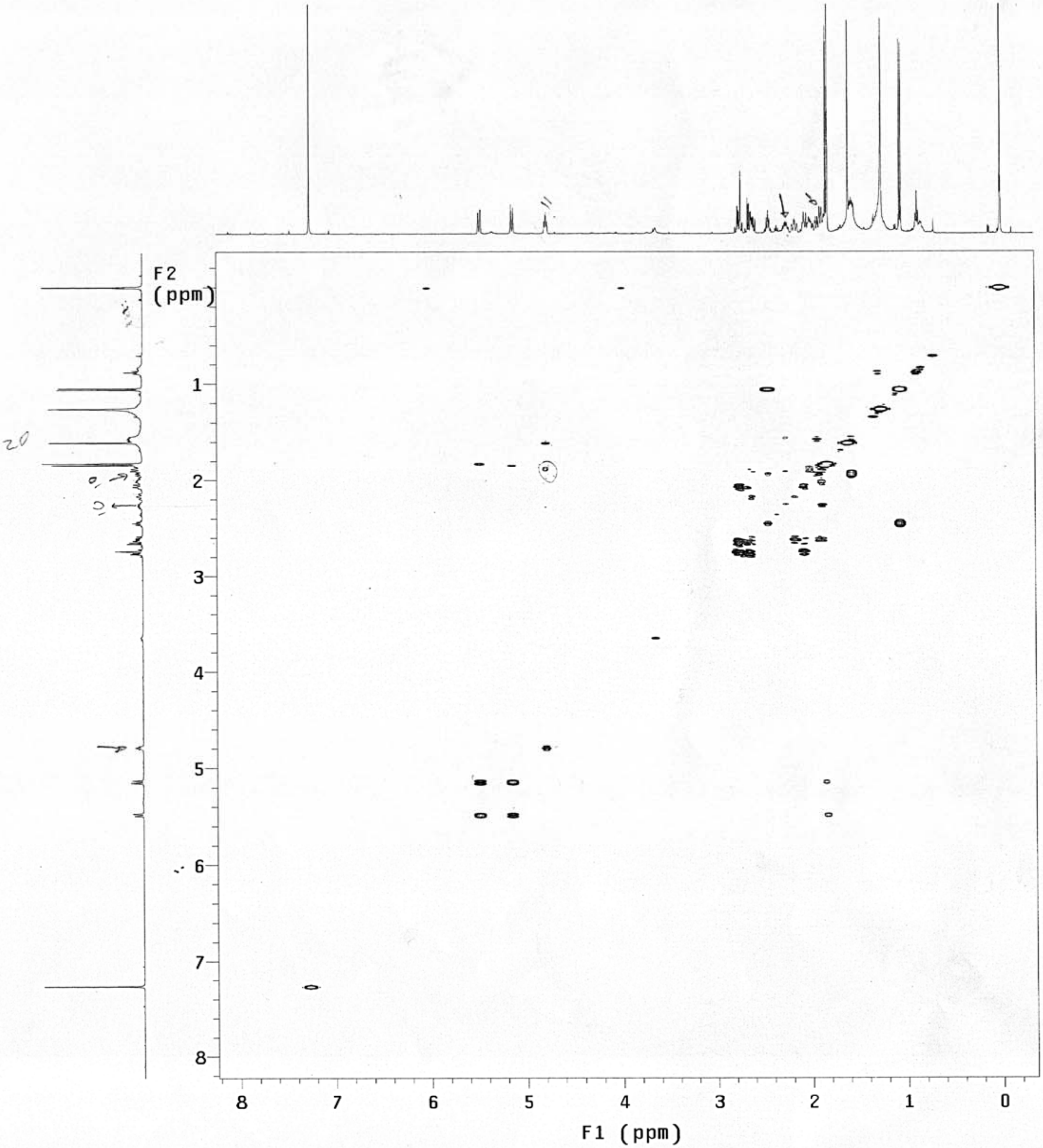

-3-2
adient HSQC
l peaks
CDCl3
Probe
ghsqc022310.1

lse Sequence: gHSQC

olvent: CDCl3
emp. 23.0 C / 296.1 K
er: 1-14-87
OVA-500 "inova500a"

elax. delay 1.000 sec
cq. time 0.205 sec
idth 5006.3 Hz
D Width 21361.8 Hz
28 repetitions
x 200 increments
SERVE H1, 499.7081755 MHz
COUPLE C13, 125.6611136 MHz
ower 43 dB
n during acquisition
ff during delay
ARP-1 modulated
TA PROCESSING
auss apodization 0.094 sec
DATA PROCESSING
auss apodization 0.011 sec
size 2048 x 2048
tal time 18 hr, 10 min, 57 sec

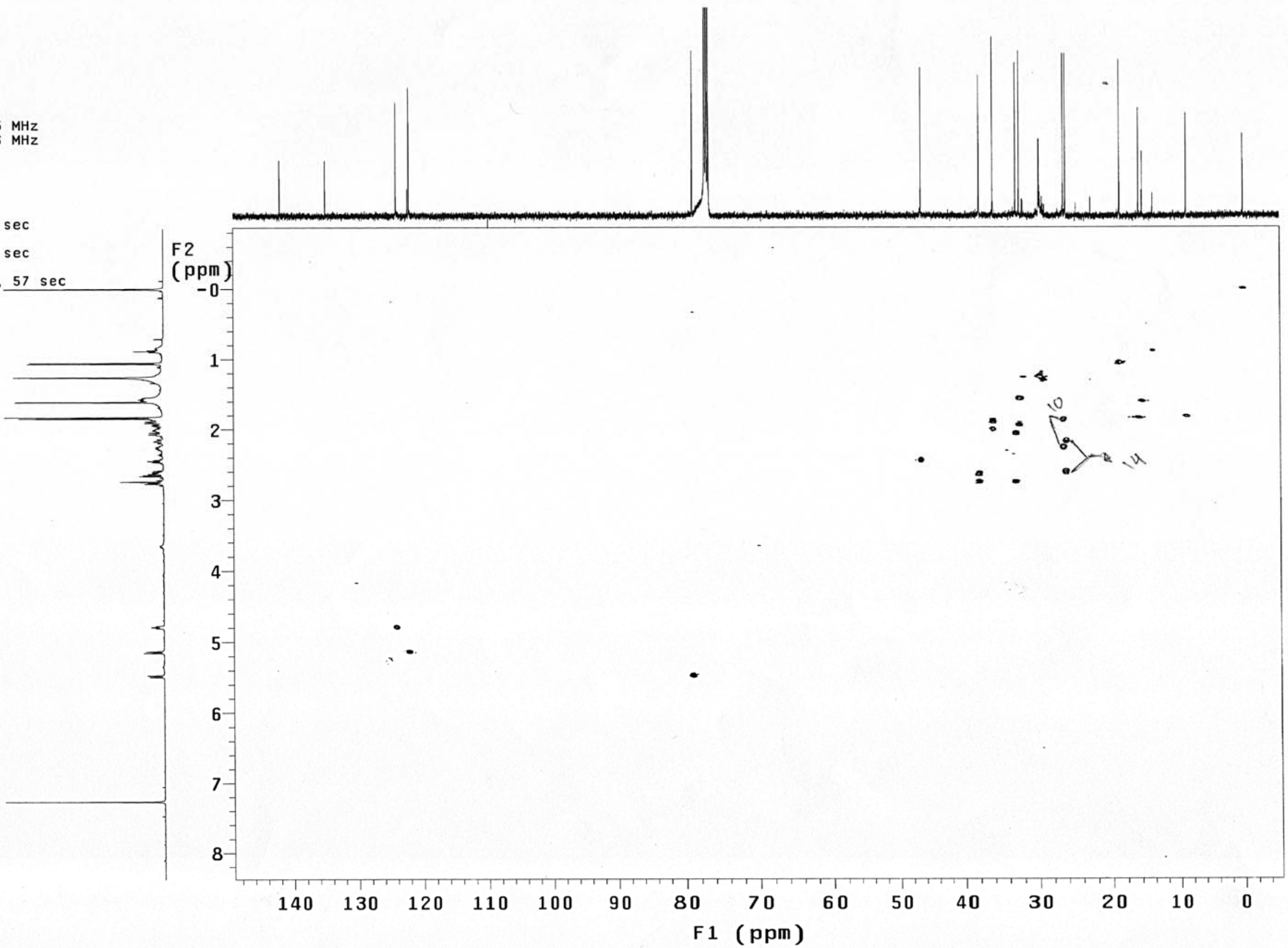

Re-3-2
Gradient HSQC
all peaks
in CDCl3
SW Probe
mkghsqc022310.1

Pulse Sequence: gHSQC

Solvent: CDCl3
Temp. 23.0 C / 296.1 K
User: 1-14-87
INOVA-500 "inova500a"

Relax. delay 1.000 sec
Acq. time 0.205 sec
Width 5006.3 Hz
2D Width 21361.8 Hz
128 repetitions
2 x 200 increments
OBSERVE H1, 499.7081755 MHz
DECOUPLE C13, 125.6611136 MHz
Power 43 dB
on during acquisition
off during delay
GARP-1 modulated
DATA PROCESSING
Gauss apodization 0.094 sec
F1 DATA PROCESSING
Gauss apodization 0.011 sec
FT size 2048 x 2048
Total time 18 hr, 10 min, 57 sec

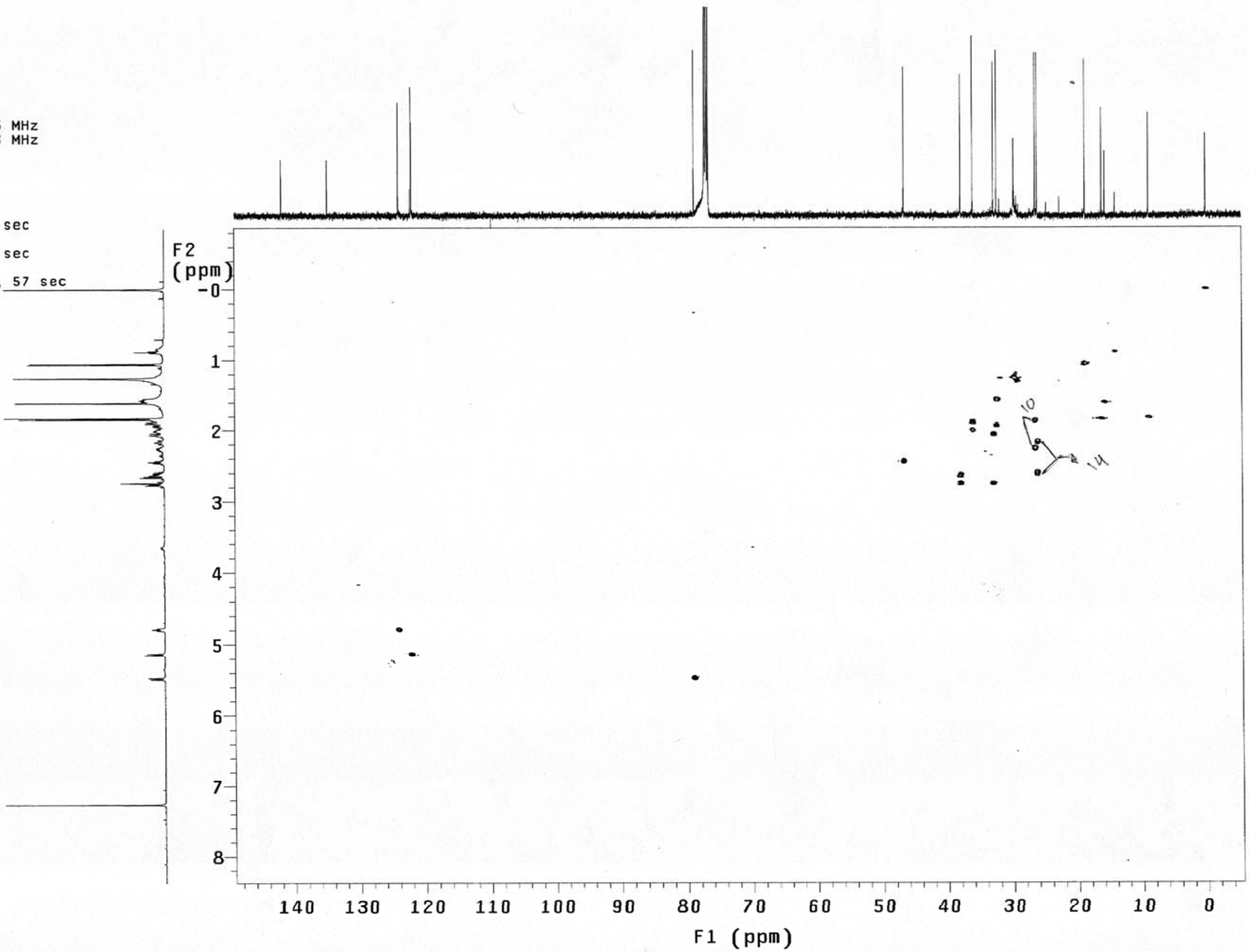

Re-3-2
Gradient HSQC
CH2 peaks only
in CDCl3
SW Probe
mkghsqc022310.1

Pulse Sequence: gHSQC

Solvent: CDCl3
Temp. 23.0 C / 296.1 K
User: 1-14-87
INOVA-500 "inova500a"

Relax. delay 1.000 sec
Acq. time 0.205 sec
Width 5006.3 Hz
2D Width 21361.8 Hz
128 repetitions
2 x 200 increments
OBSERVE H1, 499.7081755 MHz
DECOUPLE C13, 125.6611136 MHz
Power 43 dB
on during acquisition
off during delay
GARP-1 modulated
DATA PROCESSING
Gauss apodization 0.094 sec
F1 DATA PROCESSING
Gauss apodization 0.011 sec
FT size 2048 x 2048
Total time 18 hr, 10 min, 57 sec

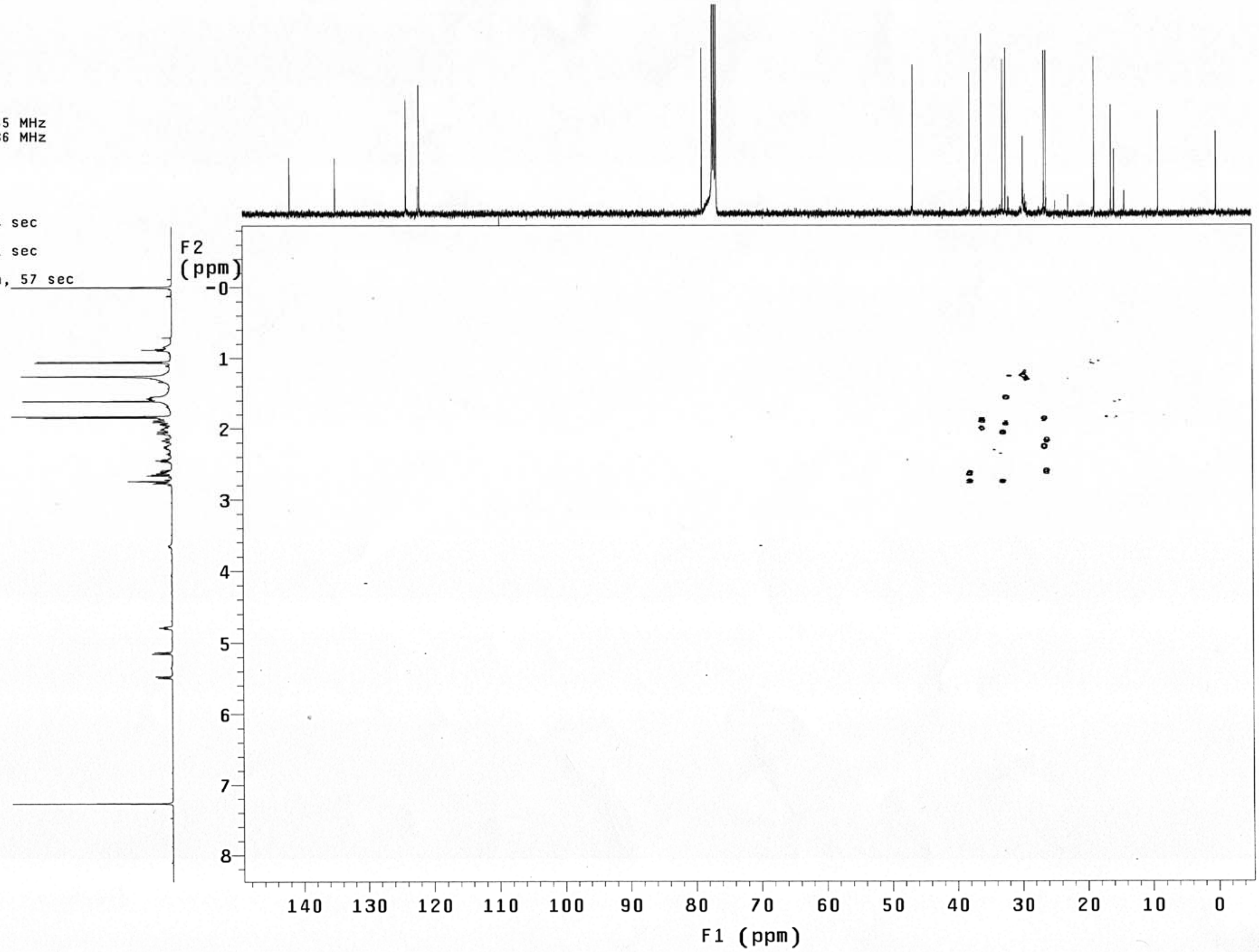

Re-3-2
Gradient HSQC
CH and CH3 peaks only
in CDCl3
SW Probe
mkghsqc022310.1

Pulse Sequence: gHSQC

Solvent: CDCl3
Temp. 23.0 C / 296.1 K
User: 1-14-87
INOVA-500 "inova500a"

Relax. delay 1.000 sec
Acq. time 0.205 sec
Width 5006.3 Hz
2D Width 21361.8 Hz
128 repetitions
2 x 200 increments
OBSERVE H1, 499.7081755 MHz
DECOUPLE C13, 125.6611136 MHz
Power 43 dB
on during acquisition
off during delay
GARP-1 modulated
DATA PROCESSING
Gauss apodization 0.094 sec
F1 DATA PROCESSING
Gauss apodization 0.011 sec
FT size 2048 x 2048
Total time 18 hr, 10 min, 57 sec

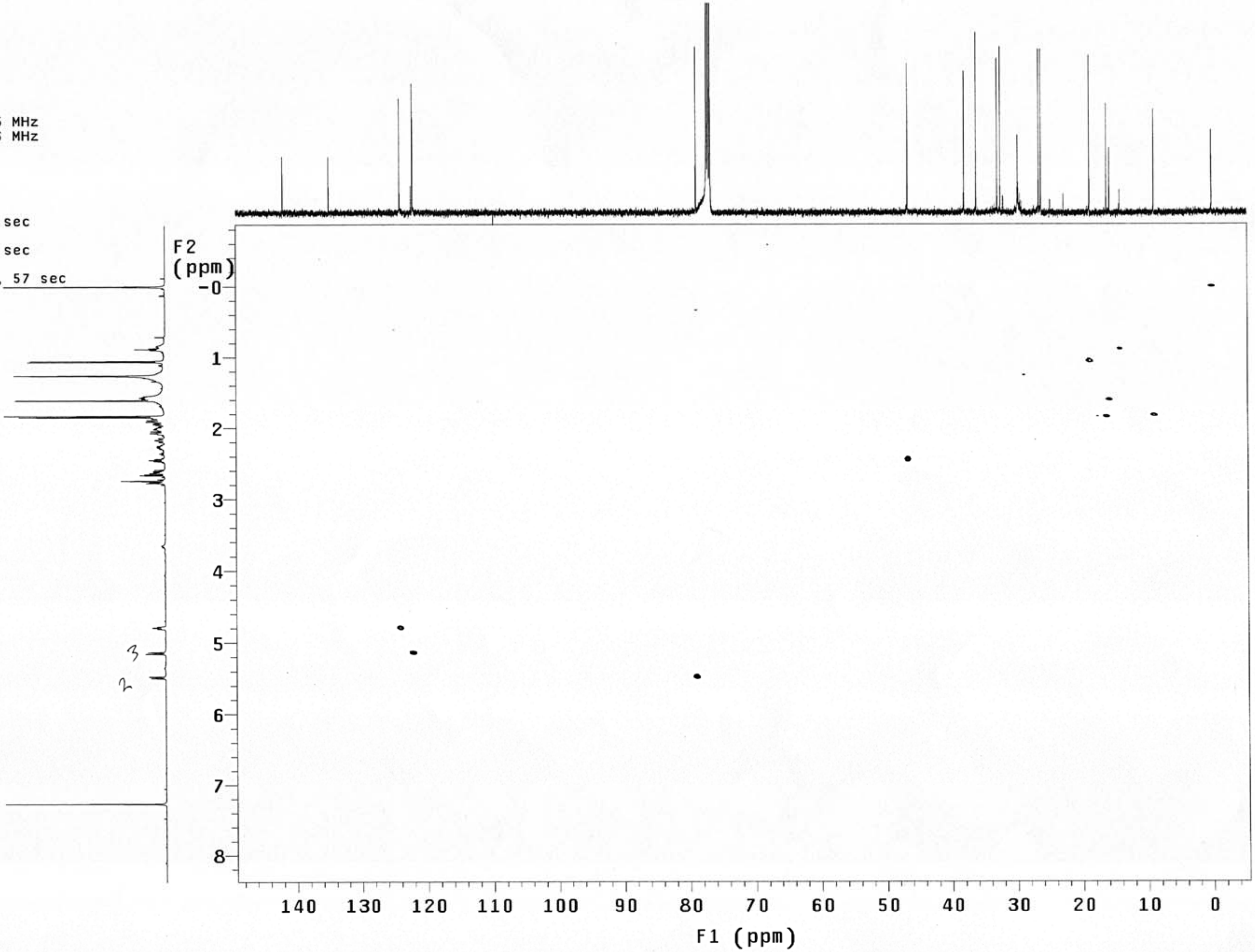

Re-3-2
Gradient HMBC
in CDCl3
SW Probe
mkghmbc022310.1

Pulse Sequence: gHMBC

Solvent: CDCl3
Temp. 23.0 C / 296.1 K
User: 1-14-87
INOVA-500 "inova500a"

Relax. delay 1.000 sec
Acq. time 0.205 sec
Width 5006.3 Hz
2D Width 30154.5 Hz
400 repetitions
400 increments
OBSERVE H1, 499.7081753 MHz
DATA PROCESSING
Sine bell 0.102 sec
F1 DATA PROCESSING
Sine bell 0.007 sec
FT size 2048 x 2048
Total time 57 hr, 28 min, 28 sec

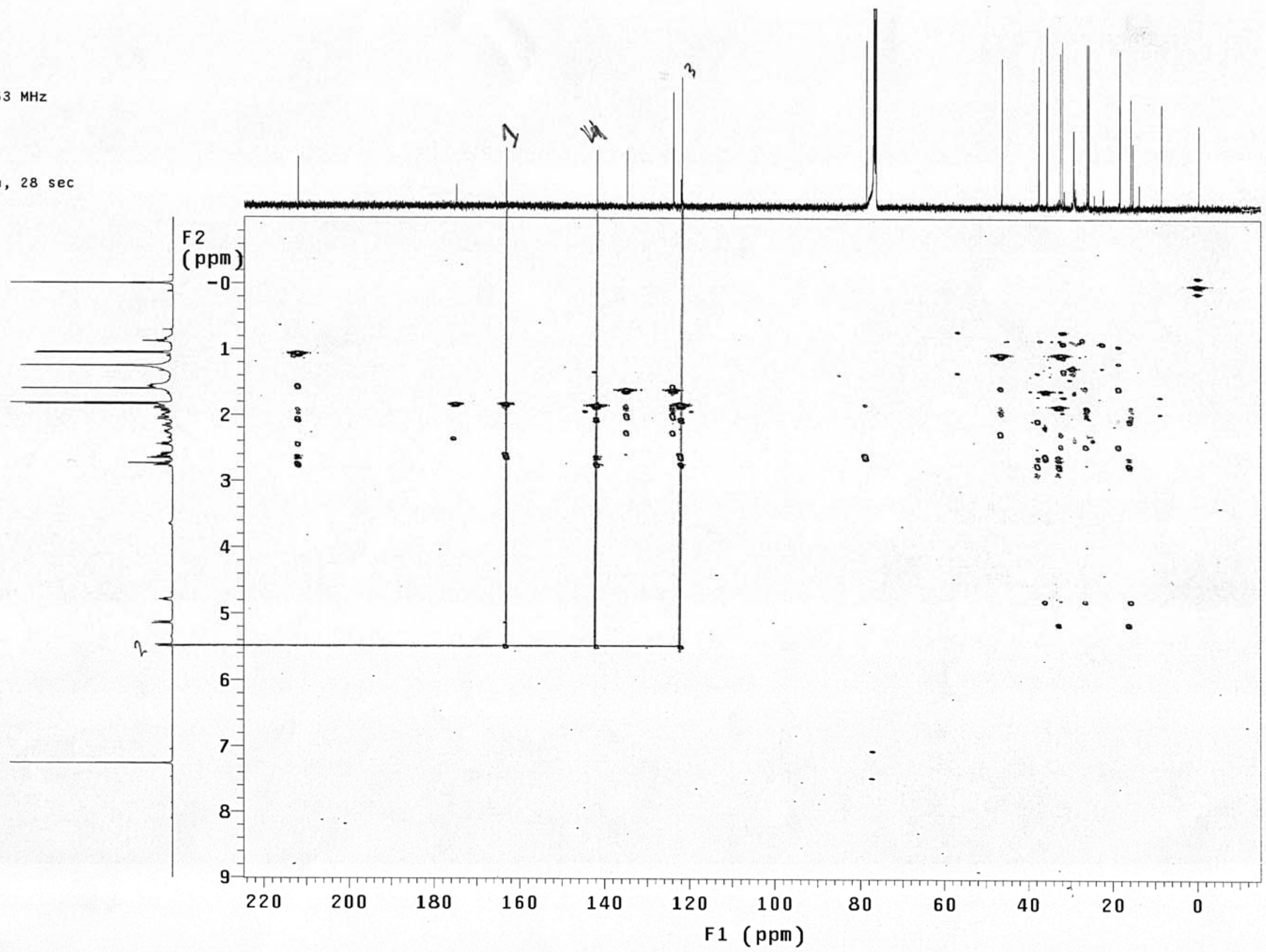

Re-3-2
NOESY
positive peaks only
d1=3 mix=1 nt=24
in CDCl3
SW Probe
mknoesy022310.1

Pulse Sequence: NOESY

Solvent: CDCl3
Temp. 23.0 C / 296.1 K
INOVA-500 "inova500a"

Relax. delay 3.000 sec
Mixing 1.000 sec
Acq. time 0.818 sec
Width 5006.3 Hz
2D Width 5006.3 Hz
24 repetitions
2 x 256 increments
OBSERVE H1, 499.7081754 MHz
DATA PROCESSING
Gauss apodization 0.094 sec
F1 DATA PROCESSING
Gauss apodization 0.037 sec
FT size 8192 x 8192
Total time 16 hr, 37 min, 22 sec

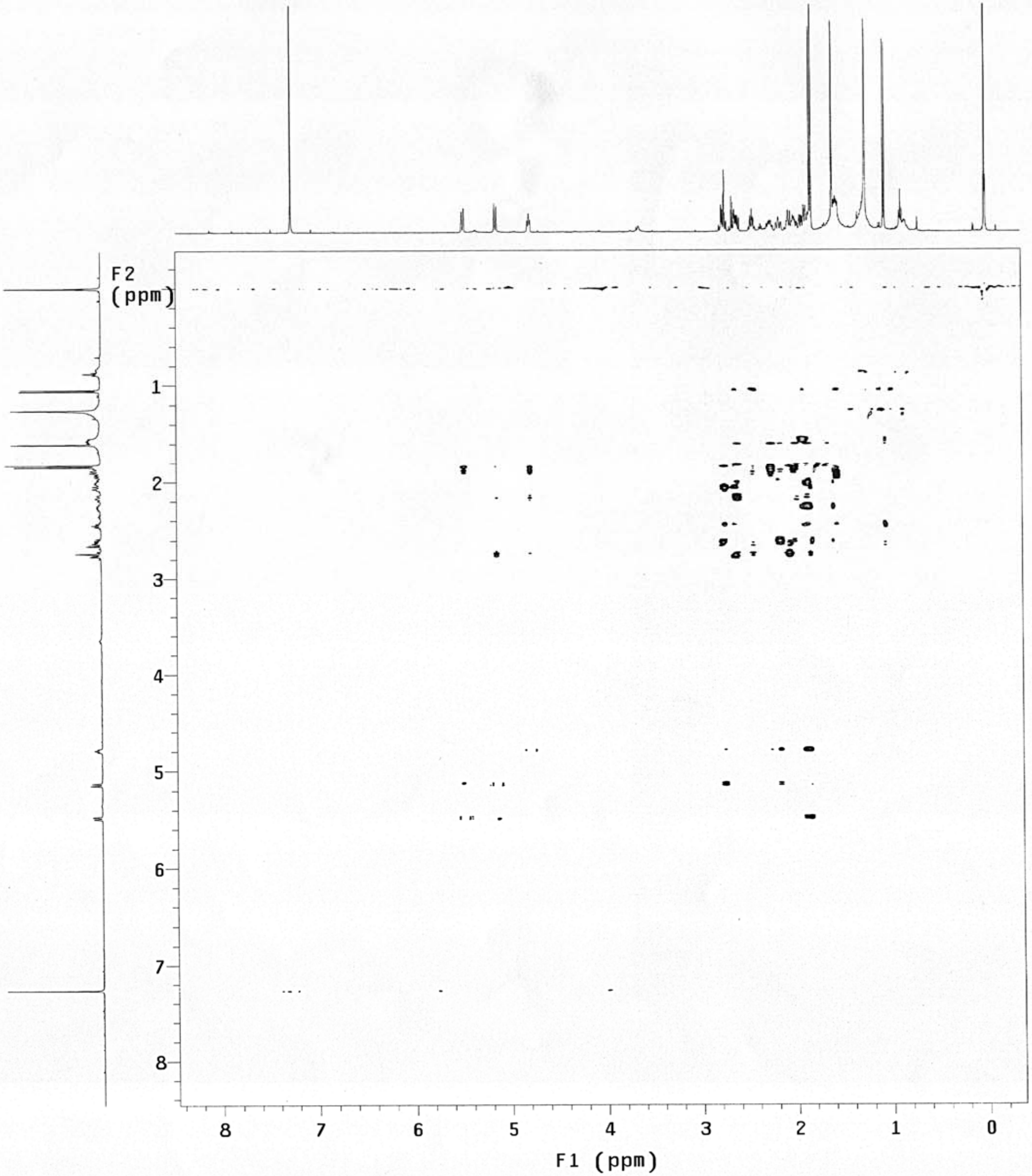

```
data_2010031601brwpare3555_0m

_audit_creation_method            SHELXL-97
_chemical_name_systematic
;
 ?
;
_chemical_name_common             ?
_chemical_melting_point           ?
_chemical_formula_moiety          ?
_chemical_formula_sum
 'C20 H28 O5'
_chemical_formula_weight          348.42

loop_
 _atom_type_symbol
 _atom_type_description
 _atom_type_scat_dispersion_real
 _atom_type_scat_dispersion_imag
 _atom_type_scat_source
 'C'  'C'   0.0033   0.0016
 'International Tables Vol C Tables 4.2.6.8 and 6.1.1.4'
 'H'  'H'   0.0000   0.0000
 'International Tables Vol C Tables 4.2.6.8 and 6.1.1.4'
 'O'  'O'   0.0106   0.0060
 'International Tables Vol C Tables 4.2.6.8 and 6.1.1.4'

_symmetry_cell_setting            ?
_symmetry_space_group_name_H-M    P 1 2(1) 1

loop_
 _symmetry_equiv_pos_as_xyz
 'x, y, z'
 '-x, y+1/2, -z'

_cell_length_a                    10.0622(18)
_cell_length_b                    5.5509(10)
_cell_length_c                    16.834(3)
_cell_angle_alpha                 90.00
_cell_angle_beta                  95.538(2)
_cell_angle_gamma                 90.00
_cell_volume                      935.9(3)
_cell_formula_units_Z             2
_cell_measurement_temperature     150(2)
_cell_measurement_reflns_used     56
_cell_measurement_theta_min       3.36
_cell_measurement_theta_max       11.74

_exptl_crystal_description        flat needle
_exptl_crystal_colour             colourless
_exptl_crystal_size_max           0.87
_exptl_crystal_size_mid           0.15
_exptl_crystal_size_min           0.02
```

```
_exptl_crystal_density_meas       ?
_exptl_crystal_density_diffrn     1.236
_exptl_crystal_density_method     'not measured'
_exptl_crystal_F_000              376
_exptl_absorpt_coefficient_mu     0.088
_exptl_absorpt_correction_type    empirical
_exptl_absorpt_correction_T_min   0.9274
_exptl_absorpt_correction_T_max   0.9982
_exptl_absorpt_process_details    ?

_exptl_special_details
;
 ?
;

_diffrn_ambient_temperature       150(2)
_diffrn_radiation_wavelength      0.71073
_diffrn_radiation_type            MoK\a
_diffrn_radiation_source          'fine-focus sealed tube'
_diffrn_radiation_monochromator   graphite
_diffrn_measurement_device_type   Bruker Smart Apex II
_diffrn_measurement_method        ?
_diffrn_detector_area_resol_mean  ?
_diffrn_reflns_number             4644
_diffrn_reflns_av_R_equivalents   0.0357
_diffrn_reflns_av_sigmaI/netI     0.0355
_diffrn_reflns_limit_h_min        -8
_diffrn_reflns_limit_h_max        8
_diffrn_reflns_limit_k_min        -4
_diffrn_reflns_limit_k_max        4
_diffrn_reflns_limit_l_min        -14
_diffrn_reflns_limit_l_max        14
_diffrn_reflns_theta_min          1.22
_diffrn_reflns_theta_max          18.45
_reflns_number_total              1364
_reflns_number_gt                 1257
_reflns_threshold_expression      >2sigma(I)

_computing_data_collection        ?
_computing_cell_refinement        ?
_computing_data_reduction         ?
_computing_structure_solution     'SHELXS-97 (Sheldrick, 2008)'
_computing_structure_refinement   'SHELXL-97 (Sheldrick, 2008)'
_computing_molecular_graphics     ?
_computing_publication_material   ?

_refine_special_details
;
 Refinement of F^2^ against ALL reflections.  The weighted R-factor wR
and
 goodness of fit S are based on F^2^, conventional R-factors R are based
 on F, with F set to zero for negative F^2^. The threshold expression of
 F^2^ > 2sigma(F^2^) is used only for calculating R-factors(gt) etc. and
is
```

```
 not relevant to the choice of reflections for refinement.  R-factors
based
 on F^2^ are statistically about twice as large as those based on F, and
R-
 factors based on ALL data will be even larger.
;

_refine_ls_structure_factor_coef  Fsqd
_refine_ls_matrix_type            full
_refine_ls_weighting_scheme       calc
_refine_ls_weighting_details
 'calc w=1/[\s^2^(Fo^2^)+(0.0209P)^2^+0.1056P] where P=(Fo^2^+2Fc^2^)/3'
_atom_sites_solution_primary      direct
_atom_sites_solution_secondary    difmap
_atom_sites_solution_hydrogens    geom
_refine_ls_hydrogen_treatment     mixed
_refine_ls_extinction_method      SHELXL
_refine_ls_extinction_coef        0.015(2)
_refine_ls_extinction_expression
 'Fc^*^=kFc[1+0.001xFc^2^\l^3^/sin(2\q)]^-1/4^'
_refine_ls_abs_structure_details
 'Flack H D (1983), Acta Cryst. A39, 876-881'
_refine_ls_abs_structure_Flack    -0.4(15)
_refine_ls_number_reflns          1364
_refine_ls_number_parameters      339
_refine_ls_number_restraints      1
_refine_ls_R_factor_all           0.0307
_refine_ls_R_factor_gt            0.0254
_refine_ls_wR_factor_ref          0.0513
_refine_ls_wR_factor_gt           0.0492
_refine_ls_goodness_of_fit_ref    1.075
_refine_ls_restrained_S_all       1.075
_refine_ls_shift/su_max           0.011
_refine_ls_shift/su_mean          0.001

loop_
 _atom_site_label
 _atom_site_type_symbol
 _atom_site_fract_x
 _atom_site_fract_y
 _atom_site_fract_z
 _atom_site_U_iso_or_equiv
 _atom_site_adp_type
 _atom_site_occupancy
 _atom_site_symmetry_multiplicity
 _atom_site_calc_flag
 _atom_site_refinement_flags
 _atom_site_disorder_assembly
 _atom_site_disorder_group
O1 O 0.3619(2) 0.2048(6) 0.77785(14) 0.0326(8) Uani 1 1 d . . .
O2 O 0.4353(2) 0.5048(5) 0.70604(15) 0.0293(8) Uani 1 1 d . . .
O3 O 0.9426(2) 1.4265(5) 0.63634(13) 0.0368(8) Uani 1 1 d . . .
O4 O 1.1459(3) 1.0112(5) 0.91293(13) 0.0338(8) Uani 1 1 d . . .
O5 O 1.2766(3) 0.9113(6) 0.89952(17) 0.0447(9) Uani 1 1 d . . .
```

```
C1 C 0.5647(4) 0.6974(8) 0.8095(2) 0.0224(10) Uani 1 1 d . . .
C2 C 0.5196(4) 0.7197(8) 0.7220(2) 0.0240(11) Uani 1 1 d . . .
C3 C 0.6244(4) 0.7328(9) 0.6655(2) 0.0238(11) Uani 1 1 d . . .
C4 C 0.6235(3) 0.8784(7) 0.6035(2) 0.0230(10) Uani 1 1 d . . .
C5 C 0.7380(5) 0.8887(9) 0.5521(3) 0.0283(12) Uani 1 1 d . . .
C6 C 0.8047(5) 1.1359(8) 0.5486(3) 0.0302(12) Uani 1 1 d . . .
C7 C 0.8606(4) 1.2139(8) 0.6295(3) 0.0243(11) Uani 1 1 d . . .
C8 C 1.0004(4) 1.1961(8) 0.6620(2) 0.0275(11) Uani 1 1 d . . .
C9 C 1.0323(5) 1.1732(9) 0.7504(2) 0.0285(13) Uani 1 1 d . . .
C10 C 1.0558(5) 0.9113(9) 0.7764(2) 0.0282(12) Uani 1 1 d . . .
C11 C 1.0477(4) 0.8686(9) 0.8655(2) 0.0249(11) Uani 1 1 d . . .
C12 C 0.9138(4) 0.9333(8) 0.8918(2) 0.0250(10) Uani 1 1 d . . .
C13 C 0.8043(4) 0.7618(9) 0.8611(3) 0.0330(12) Uani 1 1 d . . .
C14 C 0.6638(4) 0.8691(9) 0.8506(3) 0.0294(12) Uani 1 1 d . . .
C15 C 0.5051(4) 0.5130(8) 0.8404(2) 0.0222(10) Uani 1 1 d . . .
C16 C 0.4266(4) 0.3896(9) 0.7766(2) 0.0239(11) Uani 1 1 d . . .
C17 C 0.5048(6) 0.4316(10) 0.9248(3) 0.0332(12) Uani 1 1 d . . .
C18 C 0.5107(5) 1.0471(10) 0.5781(3) 0.0342(12) Uani 1 1 d . . .
C19 C 1.1051(5) 1.1029(13) 0.6126(3) 0.0486(17) Uani 1 1 d . . .
C20 C 0.8939(5) 1.1203(9) 0.9381(3) 0.0351(13) Uani 1 1 d . . .
H5 H 1.303(6) 1.005(12) 0.865(3) 0.16(3) Uiso 1 1 d . . .
H2A H 0.462(3) 0.853(6) 0.7144(16) 0.006(10) Uiso 1 1 d . . .
H3A H 0.700(3) 0.629(6) 0.6769(16) 0.011(11) Uiso 1 1 d . . .
H5A H 0.702(3) 0.837(5) 0.5002(18) 0.012(10) Uiso 1 1 d . . .
H6A H 0.879(3) 1.132(6) 0.511(2) 0.039(12) Uiso 1 1 d . . .
H7A H 0.797(3) 1.210(5) 0.6702(16) 0.007(9) Uiso 1 1 d . . .
H9A H 0.962(3) 1.228(6) 0.7752(15) 0.002(10) Uiso 1 1 d . . .
H10A H 1.151(4) 0.862(7) 0.7657(19) 0.049(13) Uiso 1 1 d . . .
H11A H 1.065(3) 0.686(7) 0.8768(16) 0.015(10) Uiso 1 1 d . . .
H13A H 0.831(3) 0.671(8) 0.811(2) 0.063(14) Uiso 1 1 d . . .
H14A H 0.661(3) 1.006(7) 0.8205(17) 0.008(11) Uiso 1 1 d . . .
H17A H 0.578(3) 0.491(6) 0.9578(17) 0.010(10) Uiso 1 1 d . . .
H18A H 0.426(4) 1.013(7) 0.603(2) 0.059(14) Uiso 1 1 d . . .
H19A H 1.080(3) 1.142(7) 0.561(2) 0.043(14) Uiso 1 1 d . . .
H20A H 0.968(3) 1.222(7) 0.9563(16) 0.028(12) Uiso 1 1 d . . .
H5B H 0.798(3) 0.778(7) 0.5719(18) 0.013(12) Uiso 1 1 d . . .
H6B H 0.740(3) 1.243(7) 0.5295(18) 0.023(13) Uiso 1 1 d . . .
H9B H 1.106(3) 1.274(6) 0.7667(17) 0.022(13) Uiso 1 1 d . . .
H10B H 0.992(3) 0.821(6) 0.7476(19) 0.034(14) Uiso 1 1 d . . .
H13B H 0.805(3) 0.611(8) 0.897(2) 0.045(13) Uiso 1 1 d . . .
H14B H 0.636(3) 0.902(7) 0.904(2) 0.037(11) Uiso 1 1 d . . .
H17B H 0.508(4) 0.253(10) 0.928(2) 0.068(17) Uiso 1 1 d . . .
H18B H 0.497(3) 1.062(6) 0.519(2) 0.042(13) Uiso 1 1 d . . .
H19B H 1.192(4) 1.188(7) 0.6301(19) 0.042(12) Uiso 1 1 d . . .
H20B H 0.808(3) 1.158(7) 0.9561(18) 0.037(13) Uiso 1 1 d . . .
H17C H 0.418(4) 0.496(8) 0.949(2) 0.079(15) Uiso 1 1 d . . .
H18C H 0.532(4) 1.205(9) 0.600(2) 0.057(16) Uiso 1 1 d . . .
H19C H 1.117(5) 0.935(10) 0.619(3) 0.09(3) Uiso 1 1 d . . .

loop_
 _atom_site_aniso_label
 _atom_site_aniso_U_11
 _atom_site_aniso_U_22
 _atom_site_aniso_U_33
```

```
 _atom_site_aniso_U_23
 _atom_site_aniso_U_13
 _atom_site_aniso_U_12
O1 0.0284(18) 0.0296(19) 0.0406(18) -0.0019(17) 0.0077(13) -0.0077(17)
O2 0.0287(16) 0.033(2) 0.0271(18) -0.0013(16) 0.0053(12) -0.0100(15)
O3 0.0412(17) 0.028(2) 0.0384(17) 0.0093(15) -0.0092(13) -0.0067(17)
O4 0.0254(19) 0.043(2) 0.0321(17) 0.0006(16) -0.0014(14) -0.0036(16)
O5 0.022(2) 0.059(2) 0.053(2) 0.015(2) -0.0015(14) 0.0001(18)
C1 0.017(3) 0.017(3) 0.033(3) -0.007(3) 0.002(2) -0.002(3)
C2 0.024(3) 0.014(3) 0.033(3) -0.003(2) 0.000(3) 0.005(3)
C3 0.021(3) 0.024(3) 0.027(3) -0.001(3) 0.004(3) 0.002(3)
C4 0.022(3) 0.023(3) 0.025(3) -0.003(3) 0.006(2) -0.006(3)
C5 0.034(3) 0.030(4) 0.020(3) 0.001(3) 0.000(3) 0.005(3)
C6 0.036(3) 0.025(4) 0.029(3) 0.005(3) 0.000(3) -0.003(3)
C7 0.026(3) 0.016(3) 0.031(3) 0.002(2) 0.005(3) -0.004(3)
C8 0.028(3) 0.028(3) 0.027(3) 0.008(2) 0.004(2) -0.005(2)
C9 0.027(4) 0.029(4) 0.030(4) -0.004(3) 0.006(3) 0.000(3)
C10 0.027(3) 0.024(4) 0.033(3) -0.005(3) -0.001(2) 0.002(3)
C11 0.030(3) 0.021(3) 0.022(3) -0.003(3) -0.004(2) -0.003(3)
C12 0.023(3) 0.026(3) 0.025(2) 0.001(3) 0.003(2) -0.001(3)
C13 0.020(3) 0.030(3) 0.049(3) -0.001(3) 0.000(2) 0.000(3)
C14 0.037(3) 0.027(3) 0.025(3) 0.007(3) 0.009(2) 0.001(3)
C15 0.018(2) 0.024(3) 0.025(3) -0.006(3) 0.000(2) 0.000(2)
C16 0.013(3) 0.024(3) 0.036(3) 0.000(3) 0.008(2) 0.000(3)
C17 0.038(3) 0.032(4) 0.029(3) -0.002(3) 0.000(3) -0.005(3)
C18 0.033(3) 0.047(4) 0.023(3) 0.003(3) 0.002(3) 0.006(3)
C19 0.035(4) 0.088(7) 0.024(4) 0.004(3) 0.006(3) 0.002(3)
C20 0.029(4) 0.043(4) 0.034(3) -0.006(3) 0.004(3) -0.006(3)

_geom_special_details
;
 All esds (except the esd in the dihedral angle between two l.s. planes)
 are estimated using the full covariance matrix.  The cell esds are taken
 into account individually in the estimation of esds in distances, angles
 and torsion angles; correlations between esds in cell parameters are
only
 used when they are defined by crystal symmetry.  An approximate
(isotropic)
 treatment of cell esds is used for estimating esds involving l.s.
planes.
;

loop_
 _geom_bond_atom_site_label_1
 _geom_bond_atom_site_label_2
 _geom_bond_distance
 _geom_bond_site_symmetry_2
 _geom_bond_publ_flag
O1 C16 1.216(4) . ?
O2 C16 1.360(4) . ?
O2 C2 1.474(4) . ?
O3 C7 1.439(5) . ?
O3 C8 1.453(5) . ?
O4 C11 1.445(4) . ?
```

```
O4 O5 1.465(3) . ?
C1 C15 1.318(5) . ?
C1 C14 1.499(5) . ?
C1 C2 1.504(5) . ?
C2 C3 1.488(5) . ?
C3 C4 1.320(5) . ?
C4 C18 1.502(5) . ?
C4 C5 1.508(5) . ?
C5 C6 1.531(6) . ?
C6 C7 1.487(5) . ?
C7 C8 1.462(5) . ?
C8 C19 1.497(6) . ?
C8 C9 1.498(5) . ?
C9 C10 1.530(6) . ?
C10 C11 1.528(5) . ?
C11 C12 1.502(5) . ?
C12 C20 1.325(5) . ?
C12 C13 1.509(5) . ?
C13 C14 1.529(5) . ?
C15 C16 1.444(5) . ?
C15 C17 1.492(5) . ?

loop_
 _geom_angle_atom_site_label_1
 _geom_angle_atom_site_label_2
 _geom_angle_atom_site_label_3
 _geom_angle
 _geom_angle_site_symmetry_1
 _geom_angle_site_symmetry_3
 _geom_angle_publ_flag
C16 O2 C2 107.8(3) . . ?
C7 O3 C8 60.8(2) . . ?
C11 O4 O5 106.5(3) . . ?
C15 C1 C14 128.2(4) . . ?
C15 C1 C2 110.2(3) . . ?
C14 C1 C2 121.6(4) . . ?
O2 C2 C3 110.7(3) . . ?
O2 C2 C1 103.1(3) . . ?
C3 C2 C1 117.7(4) . . ?
C4 C3 C2 125.7(4) . . ?
C3 C4 C18 123.7(4) . . ?
C3 C4 C5 122.0(4) . . ?
C18 C4 C5 114.3(4) . . ?
C4 C5 C6 114.7(4) . . ?
C7 C6 C5 110.7(4) . . ?
O3 C7 C8 60.1(2) . . ?
O3 C7 C6 118.4(3) . . ?
C8 C7 C6 126.0(4) . . ?
O3 C8 C7 59.1(2) . . ?
O3 C8 C19 114.9(4) . . ?
C7 C8 C19 121.2(4) . . ?
O3 C8 C9 114.3(4) . . ?
C7 C8 C9 119.0(4) . . ?
C19 C8 C9 115.3(4) . . ?
```

```
C8 C9 C10 112.2(4) . . ?
C11 C10 C9 114.0(4) . . ?
O4 C11 C12 106.9(3) . . ?
O4 C11 C10 111.0(3) . . ?
C12 C11 C10 112.7(3) . . ?
C20 C12 C11 123.5(4) . . ?
C20 C12 C13 123.3(4) . . ?
C11 C12 C13 113.2(4) . . ?
C12 C13 C14 115.5(4) . . ?
C1 C14 C13 111.8(4) . . ?
C1 C15 C16 108.2(3) . . ?
C1 C15 C17 130.9(4) . . ?
C16 C15 C17 120.9(4) . . ?
O1 C16 O2 119.4(4) . . ?
O1 C16 C15 130.0(4) . . ?
O2 C16 C15 110.5(4) . . ?

_diffrn_measured_fraction_theta_max    0.999
_diffrn_reflns_theta_full              18.45
_diffrn_measured_fraction_theta_full   0.999
_refine_diff_density_max    0.071
_refine_diff_density_min   -0.079
_refine_diff_density_rms    0.020
```

```
data_2010031501brwparere35561_0m

_audit_creation_method            SHELXL-97
_chemical_name_systematic
;
 ?
;
_chemical_name_common             ?
_chemical_melting_point           ?
_chemical_formula_moiety          ?
_chemical_formula_sum
 'C20 H28 O5'
_chemical_formula_weight          348.42

loop_
 _atom_type_symbol
 _atom_type_description
 _atom_type_scat_dispersion_real
 _atom_type_scat_dispersion_imag
 _atom_type_scat_source
 'C'  'C'   0.0033   0.0016
 'International Tables Vol C Tables 4.2.6.8 and 6.1.1.4'
 'H'  'H'   0.0000   0.0000
 'International Tables Vol C Tables 4.2.6.8 and 6.1.1.4'
 'O'  'O'   0.0106   0.0060
 'International Tables Vol C Tables 4.2.6.8 and 6.1.1.4'

_symmetry_cell_setting            ?
_symmetry_space_group_name_H-M    P2(1)2(1)2(1)

loop_
 _symmetry_equiv_pos_as_xyz
 'x, y, z'
 'x+1/2, -y+1/2, -z'
 '-x, y+1/2, -z+1/2'
 '-x+1/2, -y, z+1/2'

_cell_length_a                    9.542(3)
_cell_length_b                    10.416(3)
_cell_length_c                    19.612(6)
_cell_angle_alpha                 90.00
_cell_angle_beta                  90.00
_cell_angle_gamma                 90.00
_cell_volume                      1949.1(10)
_cell_formula_units_Z             4
_cell_measurement_temperature     150(2)
_cell_measurement_reflns_used     105
_cell_measurement_theta_min       2.08
_cell_measurement_theta_max       18.05

_exptl_crystal_description        plate
_exptl_crystal_colour             colourless
_exptl_crystal_size_max           0.5
```

```
_exptl_crystal_size_mid           0.3
_exptl_crystal_size_min           0.1
_exptl_crystal_density_meas       ?
_exptl_crystal_density_diffrn     1.187
_exptl_crystal_density_method     'not measured'
_exptl_crystal_F_000              752
_exptl_absorpt_coefficient_mu     0.084
_exptl_absorpt_correction_type    multiscan
_exptl_absorpt_correction_T_min   0.6689
_exptl_absorpt_correction_T_max   0.7442
_exptl_absorpt_process_details    ?

_exptl_special_details
;
 ?
;

_diffrn_ambient_temperature       150(2)
_diffrn_radiation_wavelength      0.71073
_diffrn_radiation_type            MoK\a
_diffrn_radiation_source          'fine-focus sealed tube'
_diffrn_radiation_monochromator   graphite
_diffrn_measurement_device_type   Bruker Smart Apex II
_diffrn_measurement_method        ?
_diffrn_detector_area_resol_mean  ?
_diffrn_reflns_number             9176
_diffrn_reflns_av_R_equivalents   0.0416
_diffrn_reflns_av_sigmaI/netI     0.0243
_diffrn_reflns_limit_h_min        -8
_diffrn_reflns_limit_h_max        8
_diffrn_reflns_limit_k_min        -9
_diffrn_reflns_limit_k_max        9
_diffrn_reflns_limit_l_min        -17
_diffrn_reflns_limit_l_max        16
_diffrn_reflns_theta_min          2.08
_diffrn_reflns_theta_max          17.97
_reflns_number_total              1332
_reflns_number_gt                 1255
_reflns_threshold_expression      >2sigma(I)

_computing_data_collection        ?
_computing_cell_refinement        ?
_computing_data_reduction         ?
_computing_structure_solution     'SHELXS-97 (Sheldrick, 2008)'
_computing_structure_refinement   'SHELXL-97 (Sheldrick, 2008)'
_computing_molecular_graphics     ?
_computing_publication_material   ?

_refine_special_details
;
 Refinement of F^2^ against ALL reflections.  The weighted R-factor wR
and
 goodness of fit S are based on F^2^, conventional R-factors R are based
 on F, with F set to zero for negative F^2^. The threshold expression of
```

```
 F^2^ > 2sigma(F^2^) is used only for calculating R-factors(gt) etc. and
is
 not relevant to the choice of reflections for refinement.  R-factors
based
 on F^2^ are statistically about twice as large as those based on F, and
R-
 factors based on ALL data will be even larger.
;

_refine_ls_structure_factor_coef  Fsqd
_refine_ls_matrix_type            full
_refine_ls_weighting_scheme       calc
_refine_ls_weighting_details
 'calc w=1/[\s^2^(Fo^2^)+(0.0136P)^2^+0.5000P] where P=(Fo^2^+2Fc^2^)/3'
_atom_sites_solution_primary      direct
_atom_sites_solution_secondary    difmap
_atom_sites_solution_hydrogens    geom
_refine_ls_hydrogen_treatment     mixed
_refine_ls_extinction_method      SHELXL
_refine_ls_extinction_coef        0.0027(5)
_refine_ls_extinction_expression
 'Fc^*^=kFc[1+0.001xFc^2^\l^3^/sin(2\q)]^-1/4^'
_refine_ls_abs_structure_details
 'Flack H D (1983), Acta Cryst. A39, 876-881'
_refine_ls_abs_structure_Flack    0.8(14)
_refine_ls_number_reflns          1332
_refine_ls_number_parameters      235
_refine_ls_number_restraints      0
_refine_ls_R_factor_all           0.0243
_refine_ls_R_factor_gt            0.0215
_refine_ls_wR_factor_ref          0.0466
_refine_ls_wR_factor_gt           0.0451
_refine_ls_goodness_of_fit_ref    1.042
_refine_ls_restrained_S_all       1.042
_refine_ls_shift/su_max           0.001
_refine_ls_shift/su_mean          0.000

loop_
 _atom_site_label
 _atom_site_type_symbol
 _atom_site_fract_x
 _atom_site_fract_y
 _atom_site_fract_z
 _atom_site_U_iso_or_equiv
 _atom_site_adp_type
 _atom_site_occupancy
 _atom_site_symmetry_multiplicity
 _atom_site_calc_flag
 _atom_site_refinement_flags
 _atom_site_disorder_assembly
 _atom_site_disorder_group
O1 O -0.1321(2) 1.1529(2) 0.29732(10) 0.0436(6) Uani 1 1 d . . .
O2 O 0.0121(2) 1.09969(19) 0.38258(12) 0.0389(6) Uani 1 1 d . . .
O3 O 0.5509(2) 0.86676(19) 0.46394(9) 0.0437(6) Uani 1 1 d . . .
```

```
O4 O 0.45788(18) 0.73560(18) 0.20531(10) 0.0364(5) Uani 1 1 d . . .
O5 O 0.3656(2) 0.76444(18) 0.14722(9) 0.0417(6) Uani 1 1 d . . .
C1 C 0.1565(3) 0.9622(3) 0.32053(16) 0.0255(8) Uani 1 1 d . . .
C2 C 0.1366(3) 1.0181(3) 0.39010(15) 0.0305(8) Uani 1 1 d . . .
H2A H 0.1158 0.9475 0.4231 0.037 Uiso 1 1 calc R . .
C3 C 0.2565(3) 1.0959(3) 0.41599(18) 0.0322(8) Uani 1 1 d . . .
H3A H 0.2976 1.1542 0.3846 0.039 Uiso 1 1 calc R . .
C4 C 0.3120(3) 1.0928(3) 0.47763(18) 0.0352(9) Uani 1 1 d . . .
C5 C 0.4379(3) 1.1730(3) 0.49402(15) 0.0425(9) Uani 1 1 d . . .
H5A H 0.4174 1.2257 0.5348 0.051 Uiso 1 1 calc R . .
H5B H 0.4560 1.2323 0.4555 0.051 Uiso 1 1 calc R . .
C6 C 0.5702(3) 1.0934(3) 0.50744(15) 0.0447(9) Uani 1 1 d . . .
H6A H 0.6519 1.1516 0.5113 0.054 Uiso 1 1 calc R . .
H6B H 0.5600 1.0474 0.5513 0.054 Uiso 1 1 calc R . .
C7 C 0.5968(3) 0.9975(3) 0.45152(15) 0.0370(8) Uani 1 1 d . . .
H7A H 0.5783 1.0300 0.4044 0.044 Uiso 1 1 calc R . .
C8 C 0.6987(3) 0.8926(3) 0.45549(17) 0.0382(9) Uani 1 1 d . . .
C9 C 0.7587(3) 0.8336(3) 0.39118(16) 0.0429(9) Uani 1 1 d . . .
H9A H 0.8590 0.8572 0.3880 0.051 Uiso 1 1 calc R . .
H9B H 0.7535 0.7390 0.3953 0.051 Uiso 1 1 calc R . .
C10 C 0.6873(3) 0.8728(3) 0.32604(17) 0.0405(9) Uani 1 1 d . . .
H10A H 0.7179 0.9505 0.3054 0.049 Uiso 1 1 calc R . .
C11 C 0.5873(3) 0.8101(3) 0.29519(17) 0.0350(9) Uani 1 1 d . . .
H11A H 0.5638 0.7278 0.3126 0.042 Uiso 1 1 calc R . .
C12 C 0.5062(3) 0.8569(3) 0.23456(14) 0.0303(8) Uani 1 1 d . . .
C13 C 0.3821(3) 0.9399(2) 0.25696(13) 0.0289(8) Uani 1 1 d . . .
H13A H 0.4192 1.0185 0.2788 0.035 Uiso 1 1 calc R . .
H13B H 0.3299 0.9668 0.2157 0.035 Uiso 1 1 calc R . .
C14 C 0.2791(3) 0.8762(3) 0.30618(14) 0.0292(8) Uani 1 1 d . . .
H14A H 0.2452 0.7946 0.2862 0.035 Uiso 1 1 calc R . .
H14B H 0.3277 0.8559 0.3494 0.035 Uiso 1 1 calc R . .
C15 C 0.0582(3) 1.0064(3) 0.27873(16) 0.0276(8) Uani 1 1 d . . .
C16 C -0.0302(4) 1.0924(3) 0.31691(18) 0.0318(8) Uani 1 1 d . . .
C17 C 0.0317(3) 0.9813(3) 0.20479(14) 0.0454(9) Uani 1 1 d . . .
H17A H 0.0879 0.9077 0.1899 0.068 Uiso 1 1 calc R . .
H17B H 0.0578 1.0572 0.1781 0.068 Uiso 1 1 calc R . .
H17C H -0.0679 0.9625 0.1979 0.068 Uiso 1 1 calc R . .
C18 C 0.2593(3) 1.0096(3) 0.53504(15) 0.0527(10) Uani 1 1 d . . .
H18A H 0.1804 0.9576 0.5189 0.079 Uiso 1 1 calc R . .
H18B H 0.2285 1.0641 0.5729 0.079 Uiso 1 1 calc R . .
H18C H 0.3349 0.9530 0.5506 0.079 Uiso 1 1 calc R . .
C19 C 0.7876(4) 0.8734(3) 0.51807(15) 0.0642(11) Uani 1 1 d . . .
H19A H 0.8174 0.7834 0.5207 0.096 Uiso 1 1 calc R . .
H19B H 0.7330 0.8952 0.5588 0.096 Uiso 1 1 calc R . .
H19C H 0.8704 0.9289 0.5155 0.096 Uiso 1 1 calc R . .
C20 C 0.5947(3) 0.9288(3) 0.18257(14) 0.0391(9) Uani 1 1 d . . .
H20A H 0.5378 0.9479 0.1422 0.059 Uiso 1 1 calc R . .
H20B H 0.6750 0.8757 0.1693 0.059 Uiso 1 1 calc R . .
H20C H 0.6284 1.0093 0.2027 0.059 Uiso 1 1 calc R . .
H5 H 0.258(5) 0.712(4) 0.168(2) 0.165(18) Uiso 1 1 d . . .

loop_
 _atom_site_aniso_label
 _atom_site_aniso_U_11
```

```
 _atom_site_aniso_U_22
 _atom_site_aniso_U_33
 _atom_site_aniso_U_23
 _atom_site_aniso_U_13
 _atom_site_aniso_U_12
O1 0.0320(13) 0.0425(14) 0.0563(15) 0.0035(12) 0.0045(14) 0.0121(13)
O2 0.0389(15) 0.0431(14) 0.0347(16) -0.0104(12) 0.0015(12) 0.0101(13)
O3 0.0451(15) 0.0370(16) 0.0490(14) -0.0003(11) 0.0013(12) -0.0086(12)
O4 0.0354(12) 0.0284(14) 0.0455(14) -0.0075(12) -0.0004(11) 0.0021(12)
O5 0.0446(13) 0.0477(14) 0.0330(13) -0.0037(12) -0.0074(13) -0.0036(12)
C1 0.027(2) 0.023(2) 0.026(2) -0.0043(18) 0.006(2) 0.0003(18)
C2 0.031(2) 0.030(2) 0.031(2) 0.0030(18) -0.0011(18) 0.010(2)
C3 0.038(2) 0.028(2) 0.031(2) -0.0046(18) 0.0034(18) -0.003(2)
C4 0.043(2) 0.032(2) 0.031(2) -0.0084(19) -0.002(2) 0.001(2)
C5 0.054(2) 0.036(2) 0.038(2) -0.0091(17) -0.005(2) -0.002(2)
C6 0.044(2) 0.046(2) 0.044(2) -0.005(2) -0.0109(18) -0.008(2)
C7 0.037(2) 0.037(2) 0.036(2) 0.000(2) -0.0021(17) -0.011(2)
C8 0.030(2) 0.041(2) 0.044(2) 0.002(2) -0.009(2) -0.003(2)
C9 0.035(2) 0.042(2) 0.051(2) 0.003(2) -0.005(2) -0.0004(17)
C10 0.037(2) 0.043(2) 0.041(2) 0.0019(19) 0.0015(19) 0.003(2)
C11 0.030(2) 0.029(2) 0.045(2) -0.0038(19) 0.001(2) 0.0029(18)
C12 0.0295(19) 0.026(2) 0.035(2) -0.0045(19) -0.002(2) -0.0006(19)
C13 0.0276(19) 0.0243(19) 0.035(2) -0.0030(16) -0.0010(17) 0.0012(18)
C14 0.0332(19) 0.0253(19) 0.0291(19) -0.0024(17) 0.0011(17) -0.0032(19)
C15 0.023(2) 0.032(2) 0.027(2) -0.0020(19) -0.001(2) 0.003(2)
C16 0.023(2) 0.030(2) 0.042(3) 0.000(2) 0.001(2) 0.003(2)
C17 0.040(2) 0.061(2) 0.035(2) -0.0036(18) -0.0036(18) 0.0081(19)
C18 0.059(2) 0.066(2) 0.033(2) -0.006(2) 0.0000(18) -0.008(2)
C19 0.068(3) 0.071(3) 0.053(2) -0.005(2) -0.026(2) 0.019(2)
C20 0.037(2) 0.041(2) 0.039(2) 0.0007(17) 0.0058(18) -0.0064(18)

_geom_special_details
;
 All esds (except the esd in the dihedral angle between two l.s. planes)
 are estimated using the full covariance matrix.  The cell esds are taken
 into account individually in the estimation of esds in distances, angles
 and torsion angles; correlations between esds in cell parameters are
only
 used when they are defined by crystal symmetry.  An approximate
(isotropic)
 treatment of cell esds is used for estimating esds involving l.s.
planes.
;

loop_
 _geom_bond_atom_site_label_1
 _geom_bond_atom_site_label_2
 _geom_bond_distance
 _geom_bond_site_symmetry_2
 _geom_bond_publ_flag
O1 C16 1.221(3) . ?
O2 C16 1.352(3) . ?
O2 C2 1.468(3) . ?
O3 C8 1.445(3) . ?
```

```
O3 C7 1.451(3) . ?
O4 C12 1.462(3) . ?
O4 O5 1.471(2) . ?
O5 H5 1.23(5) . ?
C1 C15 1.328(3) . ?
C1 C2 1.495(4) . ?
C1 C14 1.500(4) . ?
C2 C3 1.492(4) . ?
C2 H2A 1.0000 . ?
C3 C4 1.320(4) . ?
C3 H3A 0.9500 . ?
C4 C5 1.498(4) . ?
C4 C18 1.507(4) . ?
C5 C6 1.533(4) . ?
C5 H5A 0.9900 . ?
C5 H5B 0.9900 . ?
C6 C7 1.505(4) . ?
C6 H6A 0.9900 . ?
C6 H6B 0.9900 . ?
C7 C8 1.464(4) . ?
C7 H7A 1.0000 . ?
C8 C19 1.506(4) . ?
C8 C9 1.515(4) . ?
C9 C10 1.504(4) . ?
C9 H9A 0.9900 . ?
C9 H9B 0.9900 . ?
C10 C11 1.306(4) . ?
C10 H10A 0.9500 . ?
C11 C12 1.500(4) . ?
C11 H11A 0.9500 . ?
C12 C20 1.521(4) . ?
C12 C13 1.531(4) . ?
C13 C14 1.529(3) . ?
C13 H13A 0.9900 . ?
C13 H13B 0.9900 . ?
C14 H14A 0.9900 . ?
C14 H14B 0.9900 . ?
C15 C16 1.440(4) . ?
C15 C17 1.495(4) . ?
C17 H17A 0.9800 . ?
C17 H17B 0.9800 . ?
C17 H17C 0.9800 . ?
C18 H18A 0.9800 . ?
C18 H18B 0.9800 . ?
C18 H18C 0.9800 . ?
C19 H19A 0.9800 . ?
C19 H19B 0.9800 . ?
C19 H19C 0.9800 . ?
C20 H20A 0.9800 . ?
C20 H20B 0.9800 . ?
C20 H20C 0.9800 . ?

loop_
 _geom_angle_atom_site_label_1
```

```
 _geom_angle_atom_site_label_2
 _geom_angle_atom_site_label_3
 _geom_angle
 _geom_angle_site_symmetry_1
 _geom_angle_site_symmetry_3
 _geom_angle_publ_flag
C16 O2 C2 107.7(2) . . ?
C8 O3 C7 60.73(19) . . ?
C12 O4 O5 108.44(18) . . ?
O4 O5 H5 99.0(19) . . ?
C15 C1 C2 109.8(2) . . ?
C15 C1 C14 129.9(3) . . ?
C2 C1 C14 120.2(3) . . ?
O2 C2 C3 109.9(2) . . ?
O2 C2 C1 103.7(2) . . ?
C3 C2 C1 115.1(3) . . ?
O2 C2 H2A 109.3 . . ?
C3 C2 H2A 109.3 . . ?
C1 C2 H2A 109.3 . . ?
C4 C3 C2 127.3(3) . . ?
C4 C3 H3A 116.3 . . ?
C2 C3 H3A 116.3 . . ?
C3 C4 C5 120.3(3) . . ?
C3 C4 C18 124.3(3) . . ?
C5 C4 C18 115.3(3) . . ?
C4 C5 C6 113.3(2) . . ?
C4 C5 H5A 108.9 . . ?
C6 C5 H5A 108.9 . . ?
C4 C5 H5B 108.9 . . ?
C6 C5 H5B 108.9 . . ?
H5A C5 H5B 107.7 . . ?
C7 C6 C5 111.9(2) . . ?
C7 C6 H6A 109.2 . . ?
C5 C6 H6A 109.2 . . ?
C7 C6 H6B 109.2 . . ?
C5 C6 H6B 109.2 . . ?
H6A C6 H6B 107.9 . . ?
O3 C7 C8 59.44(19) . . ?
O3 C7 C6 116.7(2) . . ?
C8 C7 C6 124.6(3) . . ?
O3 C7 H7A 114.8 . . ?
C8 C7 H7A 114.8 . . ?
C6 C7 H7A 114.8 . . ?
O3 C8 C7 59.83(18) . . ?
O3 C8 C19 115.6(3) . . ?
C7 C8 C19 121.1(3) . . ?
O3 C8 C9 112.8(2) . . ?
C7 C8 C9 120.6(3) . . ?
C19 C8 C9 114.3(3) . . ?
C10 C9 C8 115.2(2) . . ?
C10 C9 H9A 108.5 . . ?
C8 C9 H9A 108.5 . . ?
C10 C9 H9B 108.5 . . ?
C8 C9 H9B 108.5 . . ?
```

H9A C9 H9B 107.5 . . ?
C11 C10 C9 126.1(3) . . ?
C11 C10 H10A 117.0 . . ?
C9 C10 H10A 117.0 . . ?
C10 C11 C12 125.6(3) . . ?
C10 C11 H11A 117.2 . . ?
C12 C11 H11A 117.2 . . ?
O4 C12 C11 101.1(2) . . ?
O4 C12 C20 109.7(2) . . ?
C11 C12 C20 113.9(2) . . ?
O4 C12 C13 110.9(2) . . ?
C11 C12 C13 110.8(2) . . ?
C20 C12 C13 110.1(2) . . ?
C14 C13 C12 115.7(2) . . ?
C14 C13 H13A 108.4 . . ?
C12 C13 H13A 108.4 . . ?
C14 C13 H13B 108.4 . . ?
C12 C13 H13B 108.4 . . ?
H13A C13 H13B 107.4 . . ?
C1 C14 C13 111.1(2) . . ?
C1 C14 H14A 109.4 . . ?
C13 C14 H14A 109.4 . . ?
C1 C14 H14B 109.4 . . ?
C13 C14 H14B 109.4 . . ?
H14A C14 H14B 108.0 . . ?
C1 C15 C16 107.9(3) . . ?
C1 C15 C17 131.1(3) . . ?
C16 C15 C17 120.9(3) . . ?
O1 C16 O2 120.5(3) . . ?
O1 C16 C15 128.6(3) . . ?
O2 C16 C15 110.9(3) . . ?
C15 C17 H17A 109.5 . . ?
C15 C17 H17B 109.5 . . ?
H17A C17 H17B 109.5 . . ?
C15 C17 H17C 109.5 . . ?
H17A C17 H17C 109.5 . . ?
H17B C17 H17C 109.5 . . ?
C4 C18 H18A 109.5 . . ?
C4 C18 H18B 109.5 . . ?
H18A C18 H18B 109.5 . . ?
C4 C18 H18C 109.5 . . ?
H18A C18 H18C 109.5 . . ?
H18B C18 H18C 109.5 . . ?
C8 C19 H19A 109.5 . . ?
C8 C19 H19B 109.5 . . ?
H19A C19 H19B 109.5 . . ?
C8 C19 H19C 109.5 . . ?
H19A C19 H19C 109.5 . . ?
H19B C19 H19C 109.5 . . ?
C12 C20 H20A 109.5 . . ?
C12 C20 H20B 109.5 . . ?
H20A C20 H20B 109.5 . . ?
C12 C20 H20C 109.5 . . ?
H20A C20 H20C 109.5 . . ?

```
H20B C20 H20C 109.5 . . ?

_diffrn_measured_fraction_theta_max    1.000
_diffrn_reflns_theta_full              17.97
_diffrn_measured_fraction_theta_full   1.000
_refine_diff_density_max    0.069
_refine_diff_density_min   -0.079
_refine_diff_density_rms    0.017
```
